# A Stopped‐Flow Instrument for Millisecond Timescale Reaction Monitoring on a Standard NMR Spectrometer

**DOI:** 10.1002/mrc.70114

**Published:** 2026-05-05

**Authors:** Andrew M. R. Hall, Edward J. King, Lloyd A. L. Mitchell, George A. Steedman, Stuart Johnstone, Clark Landis, Guy C. Lloyd‐Jones

**Affiliations:** ^1^ School of Chemistry the University of Edinburgh Edinburgh UK; ^2^ TgK Scientific Wiltshire UK; ^3^ Department of Chemistry University of Wisconsin Madison Wisconsin USA

**Keywords:** ^19^F, ^1^H, hardware, instrumentation, kinetics, line‐shape, mixing, NMR, stopped‐flow

## Abstract

We report a new stopped‐flow instrument designed for initiation and analysis of rapid reactions using standard NMR spectrometers and probes. The instrument is capable of kinetic measurements on mixing‐initiated reactions with half‐lives as short as 5 milliseconds. Design features of the instrument are discussed, with high‐speed video footage, pressure and NMR line‐shape data used to characterise mixing and stopping of reagent flows. The reactants are thermally and magnetically equilibrated prior to initiation of mixing and transport of the nascent reaction to the active volume for application of the NMR pulse sequence, ensuring near‐quantitative results. NMR data acquisition and processing require special considerations for fast reactions, and simulations were carried out to investigate the effect of composite instrument dead times on the observed rate constant. The kinetics of base‐mediated hydrolysis of methyl formate (^1^H) and pentafluorophenyl boronic acid (^19^F) are employed to demonstrate the capability of the stopped‐flow instrument for the reproducible generation of NMR data from fast reactions. Full details of the design and construction of the instrument, together with engineering and electronics drawings, are provided as supporting information. The system requires no permanent modifications to the spectrometer or console.

## Graphical abstract

A new stopped‐flow instrument allows kinetic measurements of reactions 10 milliseconds after initiating them by mixing in a flow cell inserted into a standard NMR probe and spectrometer. High‐speed video, pressure and NMR line‐shape data are used to characterise mixing and stopping, and a protodeboronation reaction with a 5‐millisecond half‐life used to test the efficiency of the system. The NMR data acquisition and processing require special considerations and simulations are used to investigate the effect of composite dead times.

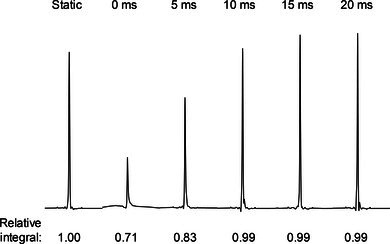

## 1 Introduction

Stopped‐flow instruments automate the process of mixing reagents to initiate a reaction and start data collection. They offer a key advantage of allowing the spectroscopic analysis of the kinetics of fast reactions in a highly reproducible manner. Stopped‐flow instruments typically consist of two or more syringes containing reagent solutions that are simultaneously driven, at high flow rates, through a mixer to initiate the reaction of interest and then on to an observation cell. Once a steady‐state of the nascent reaction mixture has been achieved in the dynamic flow in the observation cell, the flow is rapidly stopped, often by use of a ‘stop syringe’ that hits a physical barrier. The section of the previously flowing nascent reaction mixture that is trapped in the observation cell is then interrogated spectroscopically over a period of time to gain the required kinetic information [1, 2, 3, 4, 5]. The period of time between initiation of the reaction by mixing and the beginning of the spectroscopic measurement is referred to as the ‘dead time.’ The longer this dead time, the greater the initial period of the reaction evolution that is absent in the acquired spectroscopic dataset.

A number of designs for stopped‐flow NMR (SF‐NMR) instruments have been published [1, 2, 4, 6, 7, 8, 9, 10, 11]. The designs can broadly be grouped into those based on a custom stopped‐flow NMR probe [4, 7, 8, 10], and those based on auxiliary equipment applied to standard NMR probes and hardware [1, 2, 6, 9, 11]. Custom‐built stopped‐flow probes typically offer smaller sample volumes and shorter dead times, because the probe can be designed to minimise the distance travelled by the nascent reaction mixture to the active volume. An early example of a SF‐NMR probe, reported by Ernst, achieved a dead time of 3 milliseconds between initiating the reaction and acquisition of the first spectrum [7]; however, the probe is incompatible with modern shielded‐magnet spectrometers. Conversely, designs intended to fit standard spectrometer probes are more versatile and better suited to NMR facilities with multiple users. They also offer the possibility of using cryoprobes to increase the sensitivity of the NMR measurement. The limited space within the sample transit and probe regions of a standard bore magnet, together with the fixed sample volume of the probe, pose major challenges for reducing the time that it takes to mix the reagent solutions and deliver nascent reaction to the NMR active volume. Accordingly, the dead times for stopped‐flow measurements using auxiliary equipment inserted into a standard spectrometer probe are typically between one and two orders of magnitude greater than for custom flow probes.

**FIGURE 1:**
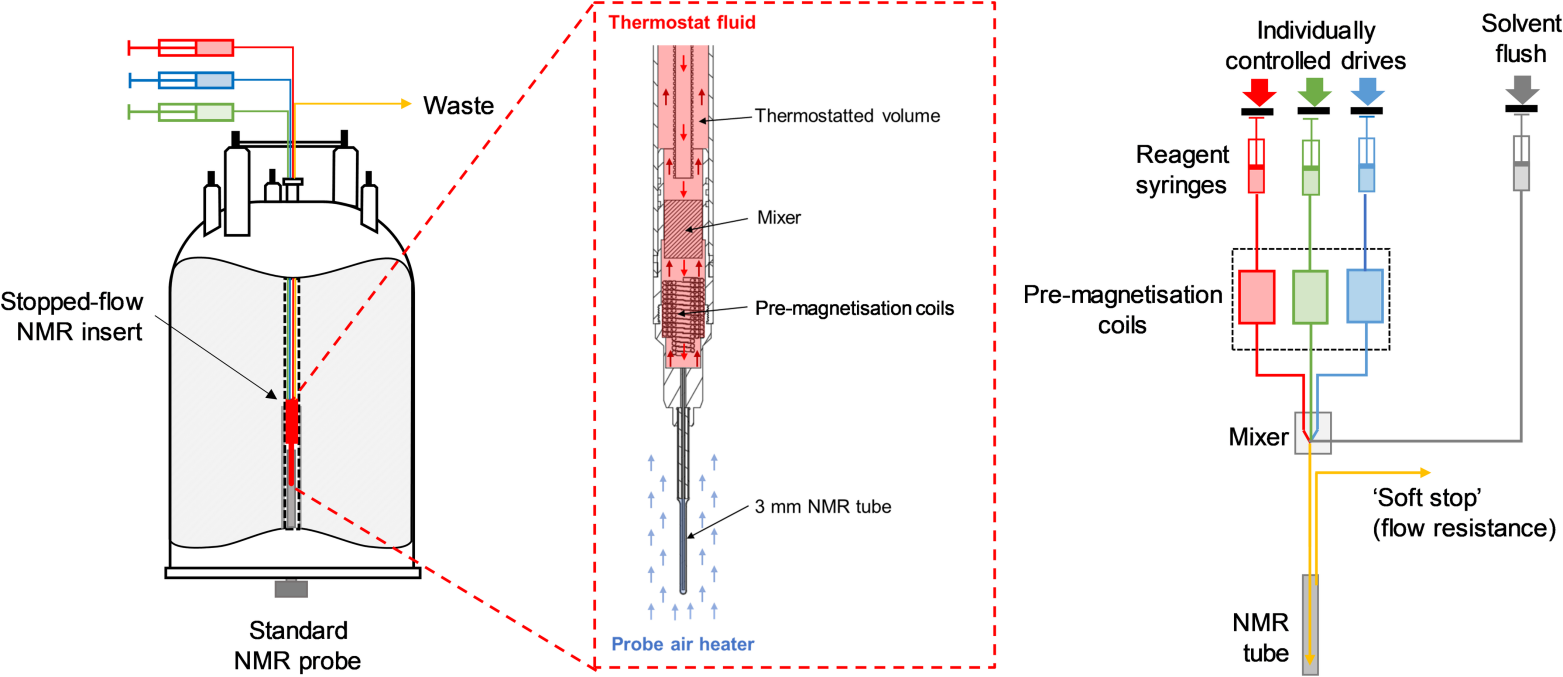
Schematic of the design and features of the 2018 SF‐NMR‐V1 system [1, 18, 19]. The system uses three independently controlled syringe drives and a resistance enabled soft‐stop. The dead time is approximately 150 milliseconds [1, 2]; see Figure 2.

In 2018, we reported the design of a new SF‐NMR insert with three individually controllable syringe drives, allowing precise and automated dosing of reagent solutions, via a rapid mixer, into a 3‐mm OD NMR tube located within the spectrometer probe (Figure 1) [1]. Reagents are stored within thermostatically controlled premagnetisation coils located in close proximity to the magnet isocentre, ensuring that near equilibrium levels of polarisation are reached before initiating the reaction. Activation of the three syringe drives pushes reagent solutions out from the premagnetisation coils through a high‐efficiency mixer to initiate the reaction. The nascent reaction mixture then flows from the mixer, through a capillary held concentrically in the NMR tube, to enter at the base. The previous contents of the NMR tube are displaced by the incoming flow of nascent reaction mixture, and the waste vented via tubing that eventually exits the magnet. When the syringe drives are halted, flow resistance within the system brings the fluid to a stop within approximately 30 milliseconds, trapping a portion of the reaction mixture within the NMR active volume. The reaction is then monitored by a series of NMR acquisitions, with carefully chosen prescan and interscan delays. An advantage of a three‐syringe drive design is that the volume of each reagent delivered can be independently controlled, allowing a range of reagent ratios to be studied from a single set of stock solutions. If the third syringe is filled with solvent only, then the system can be used to automatically and reproducibly dilute the contents of the other two stock solutions. We designed and built this first version instrument [1, 2], termed herein as SF‐NMR‐V1, to study a range of processes, including organoboron speciation [12, 13], organosilicon anion transfers [1, 14, 15], Lewis base catalysis [16], and reactions of titanocene sulfides [17]. Our SF‐NMR‐V1 system was subsequently commercialised by Bruker, as InsightXpress, and has been applied in a range of academic and industrial settings [18, 19].

While SF‐NMR‐V1 enabled us to investigate relatively rapid and thermally sensitive chemistries in detail, it also revealed processes that had proceeded to completion within the 150‐millisecond dead time of the system and had thus evaded detailed analysis. This factor was the impetus for the construction of a new instrument, SF‐NMR‐V2, again for use on standard NMR spectrometer probes, but with a considerably reduced dead time. Herein, we report on the design and testing of this system.

## 2 Materials and Methods

### 2.1 Dead Time

As discussed above, stopped‐flow instruments are typically characterised by their dead time, the delay between mixing the reagents to initiate the reaction and the earliest point at which a measurement can be taken of the evolving reaction mixture. The dead time determines the age of the sample when it reaches the detection cell. A shorter dead time allows reactions with faster rates to be observed, because less of the reaction has taken place prior to acquisition of the first spectrum.

**FIGURE 2:**
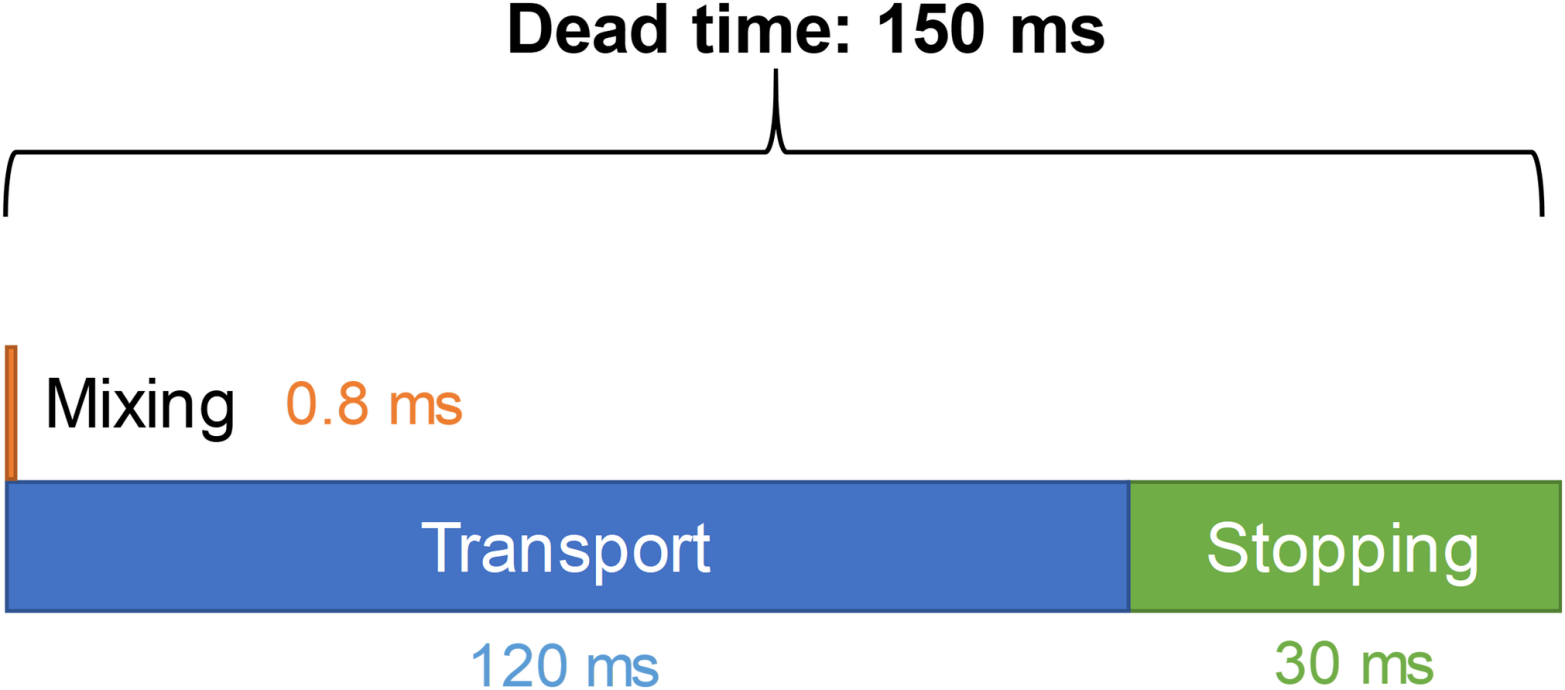
Breakdown of the three components contributing to the dead time of a stopped‐flow device. In systems using a passive flow‐through mixer, mixing occurs concurrently with sample transport. The times indicated are those determined for SF‐NMR‐V1; see Figure 1.

The dead time has the following three components: (1) the time taken to physically mix the reagents (mixing time), (2) the time to transport the nascent reaction to the observation cell (transport time) and (3) the time taken to stop the flow, trapping a portion of the reaction mixture in the observation cell (stopping time). These components are illustrated in Figure 2 using values from SF‐NMR‐V1, which has an overall dead time of 150 milliseconds.

For passive flow‐through mixers, as used in SF‐NMR‐V1, the mixing is concurrent with the transport. To reduce the dead time, it is the transport and stopping times that must therefore be addressed, while maintaining rapid mixing within the transport time. Minimising the distance between the initiation of mixing and the detection volume is an obvious method to ensure short transport times, and this is exploited in classic stopped flow systems using spectroscopic techniques such as UV–Vis and IR. However, the limited space available within superconducting actively shielded magnet bores makes short transport times much more challenging for NMR spectroscopy. In SF‐NMR‐V1, the mixer is located 165 mm above the detection region of the NMR probe (Figure 1), with the nascent reaction flowing down a 0.5‐mm diameter capillary into the NMR tube. This position was selected to be as close as possible to the NMR tube; however, geometry restrictions and the need to locate the premagnetisation coils within the strongest magnetic field prevented the mixer from being any closer to the NMR active volume. At the 1 mL/s flow rate delivered by the three stepper‐motor syringe drives employed for SF‐NMR‐V1, the nascent reaction takes 120 milliseconds to reach the detection volume.

### 2.2 Mixer Position and Design

**FIGURE 3:**
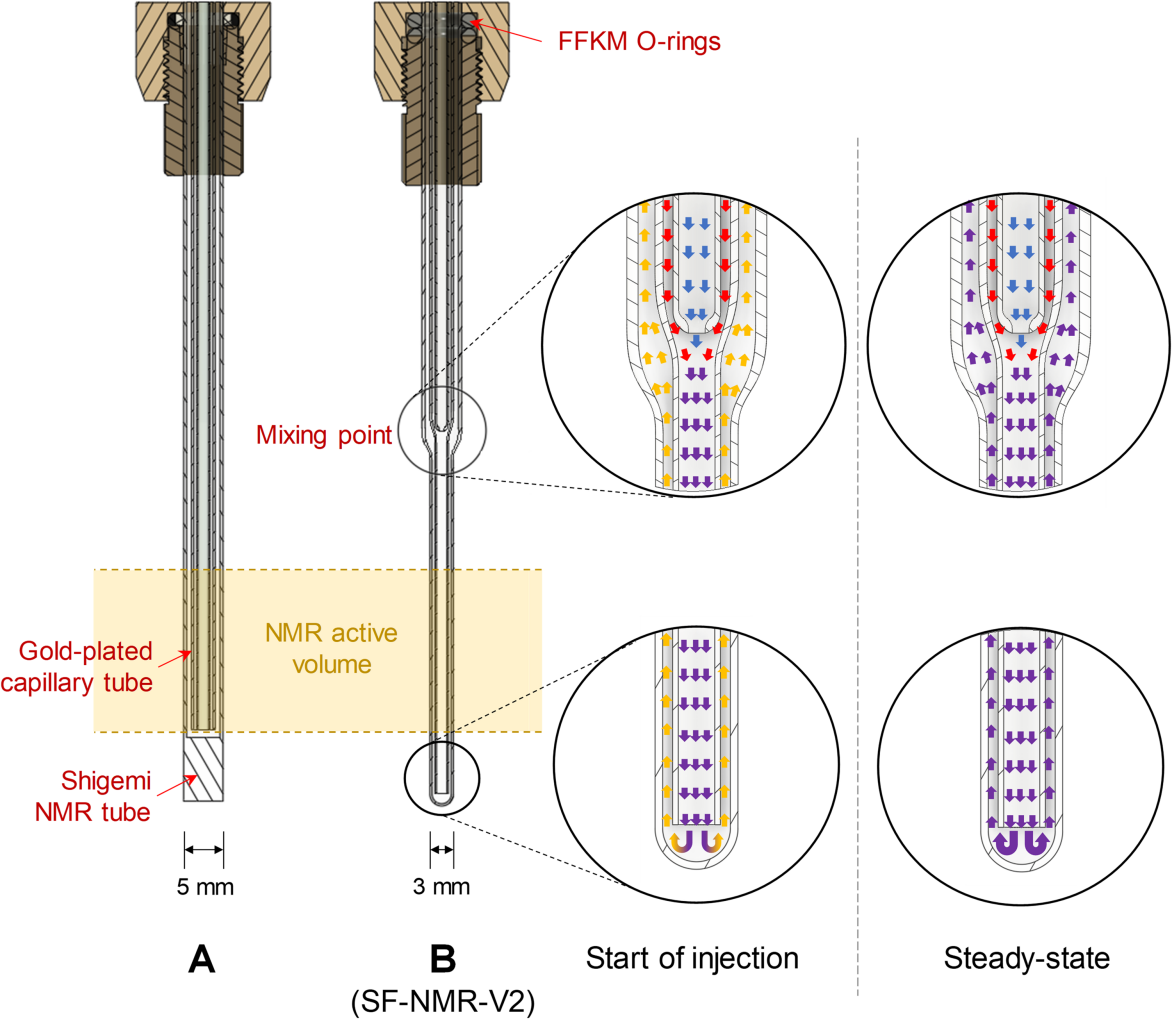
Comparison of NMR flow cell designs: (A) an RF‐shielded prototype using a magnetically matched Shigemi tube; (B) the final system for SF‐NMR‐V2, reported herein. The initial prototype for a faster instrument used a gold‐plated capillary tube to shield unmixed reagents from detection (see Supporting Information for details). The final design, SF‐NMR‐V2, uses a passive mixer constructed from two concentric capillary tubes with constricted ends. Reagents (red and blue arrows) meet at a point 18 mm above the NMR active volume. Flow‐induced turbulent mixing (Reynolds number 1.2 × 10^4^) of the reagents initiates the reaction. This fills the NMR cell, first through the central capillary (purple arrows), and then upwards through the annular space between the capillary and NMR tube to exit at the top of the NMR tube. After a sufficient period of steady‐state flow, the previous contents of the cell (yellow arrows) is fully replaced by nascent reaction (purple). The flow is then rapidly arrested, leaving a solution of the nascent reaction in the active volume region of both the central capillary and the annular space between the capillary and NMR tube. See text for full discussion.

In SF‐NMR‐V1, rapid mixing is achieved by collisional confluence of the three reagent streams in a custom‐built PEEK micromixer block located in a separate zone above the NMR flow cell (Figure 1). For SF‐NMR‐V2, we have developed a passive mixer that is small enough to locate *within* the NMR flow cell. This substantially reduces the distance that the nascent reaction travels before detection. Our initial attempts to achieve this used concentric glass capillary tubes to deliver the reagents to the bottom of a Shigemi NMR tube (Figure 3A) The solutions rebound from the flat inner surface of the glass base to pass up and through the annular gap and out of the system, resulting in moderately effective passive mixing. To avoid the unmixed reagents inside the capillary tubes from being observed in the NMR measurement, the outer capillary tube was coated in gold foil, shielding the contents from the radiofrequency pulses. However, this design proved challenging in practice, as the gold foil resulted in significant changes to the RF matching of the NMR probe, and perfect alignment of the glass capillaries was required to achieve proper mixing (see Supporting Information for further details).

The final design of the passive mixer used in SF‐NMR‐V2 is shown in Figure 3B. The system again uses two concentric glass capillary tubes. The inner tube contains the first reagent and terminates approximately 18 mm above the NMR active volume. The internal diameter at the end of this inner capillary is reduced to form a constriction, which results in a high‐speed jet of reagent. The outer capillary contains the second reagent and has a reduction in diameter just below the point at which the inner capillary terminates. This has the effect of funnelling the two reagents together at high speed, leading to turbulence and efficient mixing. The mixed reagents flow down the capillary through the active volume to the bottom of the NMR tube, with the flow then returning upwards via the annular space between the capillary and the outer tube, passing once again through the active volume before exiting to waste.

**FIGURE 4:**
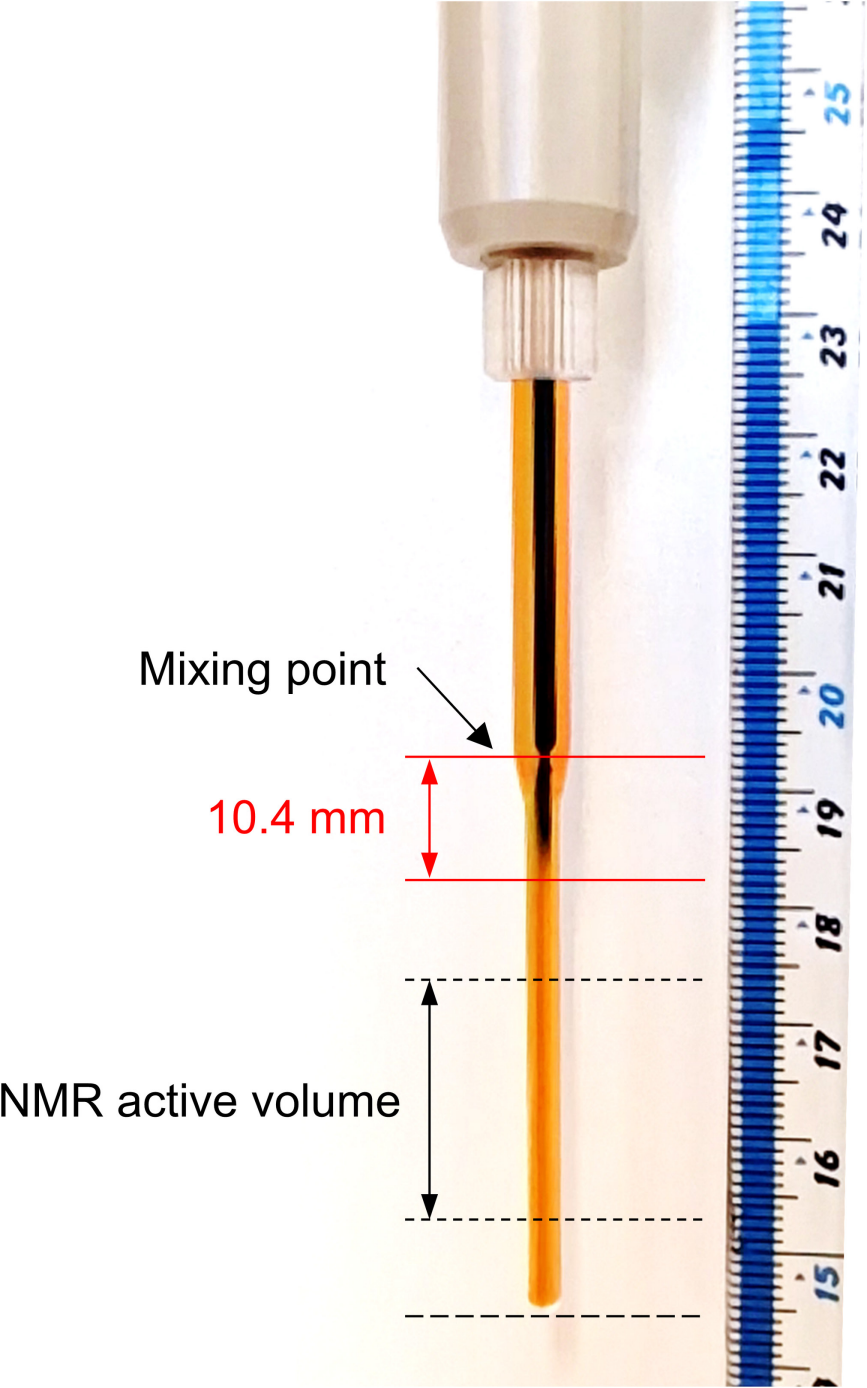
Still image from high‐speed video footage (960 frames per second) recorded during the mixing of and ethanol solution of bromothymol blue (inner capillary) and an ethanolic solution of hydrochloric acid (outer capillary) at a combined flowrate of 10 mL/s. Mixing is visually complete (orange colour of the acidic form of bromothymol blue) within a 10.4 ± 0.6‐mm region (average of 6 repeats) after combining the reagents, corresponding to a mixing time of 1.4 ± 0.08 milliseconds. Video footage of the mixing process is available in the Supporting Information. The flow at the mixing point is highly turbulent (the Reynolds number 1.2 × 10^4^, substantially above the threshold for complete transition, 2.9 × 10^3^).

The efficiency of the mixing can be analysed visually using bromothymol blue, which immediately changes from deep blue to yellow on micromixing with excess acid. Figure 4 shows the effect of mixing an ethanol solution of bromothymol blue (inner capillary) with dilute hydrochloric acid in ethanol (outer capillary) at a flow rate of 10 mL/s. A conical jet of blue dye can be seen entering the mixing zone, with complete mixing occurring at a distance corresponding to 1.4 ± 0.08 milliseconds. The yellow solution of the acid form of the indicator can be seen passing down the central capillary to vent at the tube base and then return up the annular space between the central capillary and the outer tube, before exiting the flow cell. The mixing behaviour is very reproducible and consistent throughout the entire reagent injection (see video provided as Supporting Information).

### 2.3 Transport Time

Although the newly designed mixer (Figures 3B and 4) is highly efficient and substantially reduces the distance between the mixing point and detection, it is only effective at combined flow rates in excess of 6 mL/s. Moreover, it assumes equal flow rates of both reagents and is therefore unsuitable for application in any adaptation of the syringe drives used in the SF‐NMR‐V1 instrument. Instead, SF‐NMR‐V2 uses a substantially more powerful unit in which a single stepper motor drives two syringes simultaneously and is capable of delivering combined flow rates of up to 12 mL/s. Increasing the flow rate of the reagent solutions and the nascent reaction obtained after mixing results in an increased back‐pressure resulting from friction of the fluid with the tubing wall. According to the Darcy–Weisbach relationship, Equation (1), the back‐pressure that is generated is inversely proportional to the tubing diameter and proportional to the square of the flow velocity.

(1) $$∆P=L\times f_{D}\times \frac{\rho }{2}\times \frac{{\left\langle v\right\rangle }^{2}}{D_{h}}$$

$$\begin{array}{c} ∆P=\text{back pressure},L=\text{length},\cdot f_{D}=\text{wall friction coefficient},\\ \;\rho =\text{fluid density},\left\langle v\right\rangle =\text{mean flow velocity},\text{and}\;D_{h}=\text{diameter} \end{array}$$

**FIGURE 5:**
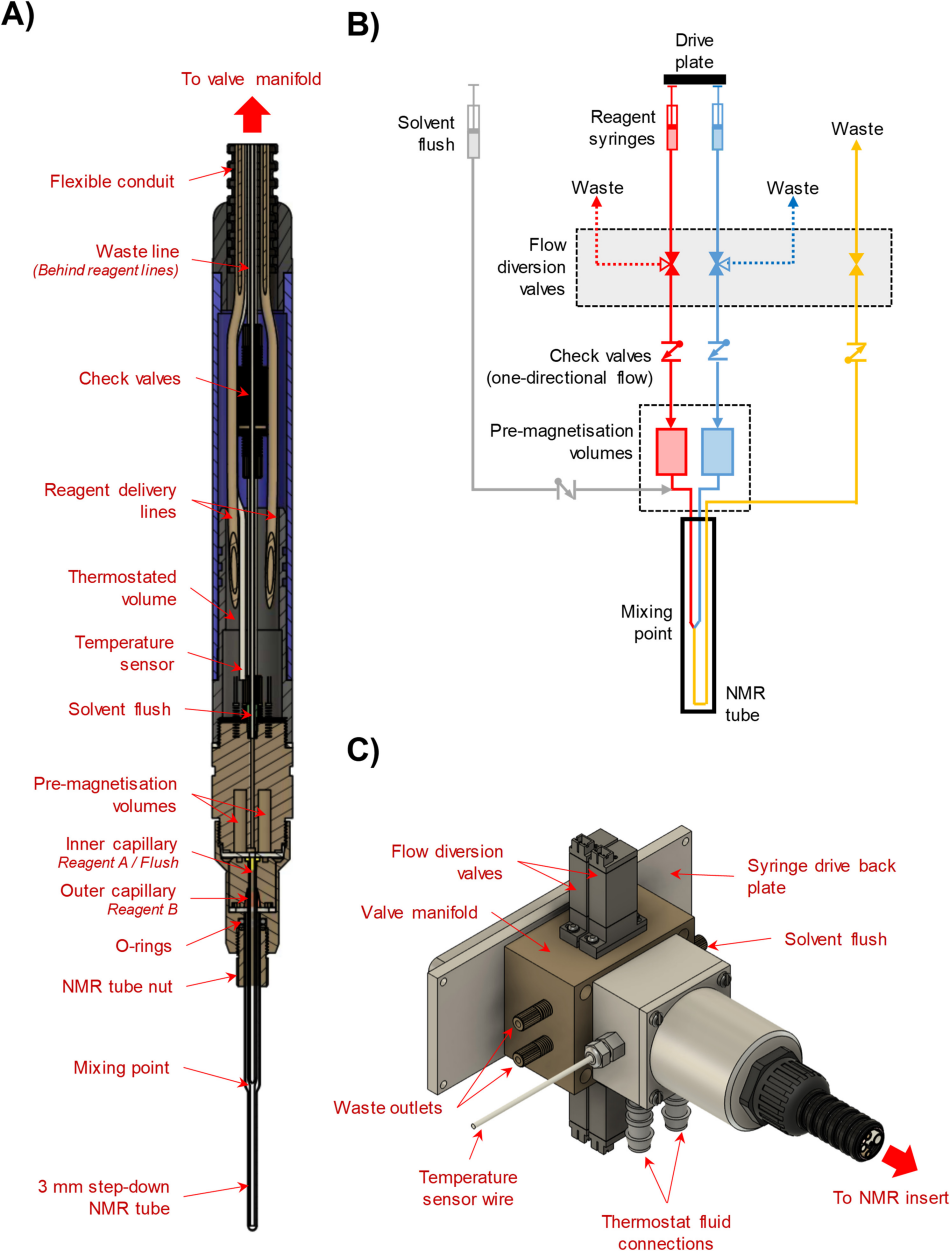
(A) Cross‐section of the stopped‐flow NMR insert, showing key components and features. (B) Schematic showing reagent flow paths during normal operation. When the flow diversion valves are activated, flow from the reagent syringes is diverted to waste (red and blue dashed lines), and the valve on the waste line from the NMR tube (yellow line) is closed. (C) Valve manifold mounted on the rear of the syringe drive, showing flow diversion valves and connections. For further details and engineering drawings of all components, see the Supporting Information.

The increase in the flow rate thus requires an increase in tubing diameter to avoid generating excessive back‐pressure that may overload the syringe drive or rupture tubing. However, increasing tubing diameter has the undesirable effect of increasing transport time, due to the larger volume of reagent that must be displaced, along with requiring a larger overall reagent volume, increasing reagent costs. There is therefore an optimum flow rate and tubing inner diameter and length, balancing the requirements for high velocity without generating excessive back‐pressure. For SF‐NMR‐V2 (Figure 5), this was determined by simulations and experimental measurements to be 1200‐mm long tubing with 2.0‐mm inner diameter for the reagent delivery lines, and 2.4‐mm diameter for the waste line, at a total flow rate of 10 mL/s. The length is the minimum required to connect the syringe drive to the SF‐NMR insert within the NMR probe, and the different diameters accommodate the flow rate in the waste line being twice that of the reagent delivery lines.

### 2.4 Stopping Time

A consequence of increasing the flow rate of the reagents solutions and reaction mixture passing from the drive through the NMR flow tube and ultimately exiting at the waste stream terminus is that the fluid has higher net momentum. This increases the time required for the flow to stop after the syringe drive stops pushing. Conventional pneumatically driven stopped‐flow instruments utilise a ‘hard stop,’ whereby the waste stream exiting the detection cell fills a ‘stopping syringe’. A rigid physical barrier is used to stop the plunger that emerges from the stopping syringe after it has filled to a preselected volume, rapidly arresting the flow within the whole system. The hard stop results in a pressure spike that propagates back through the system (‘water hammer’ effect) to the pneumatic‐drive. In these systems the fluid flow is ballistic, limited only by the gas pressure available to the pneumatic‐drive and the resistance to flow, with the pressure spikes on stopping being rapidly and safely damped by the gas‐spring effect of the pneumatic drive. In contrast, high torque stepper‐motor drives enable very controlled constant or ramped flow fluid delivery but must be stopped smoothly due to inertia in the motor. Consequently, a stepper motor driven system connected to a stopping syringe will induce acute overpressurisation and damage to the system. For this reason, the SF‐NMR‐V1 (InsightXpress) instrument [1, 2, 18, 19] was designed around use of a ‘soft stop,’ with an open vented waste stream, and deceleration of the fluid occurring smoothly through the movement‐induced friction within the flow path once the drive syringes stopped pushing.

**FIGURE 6:**
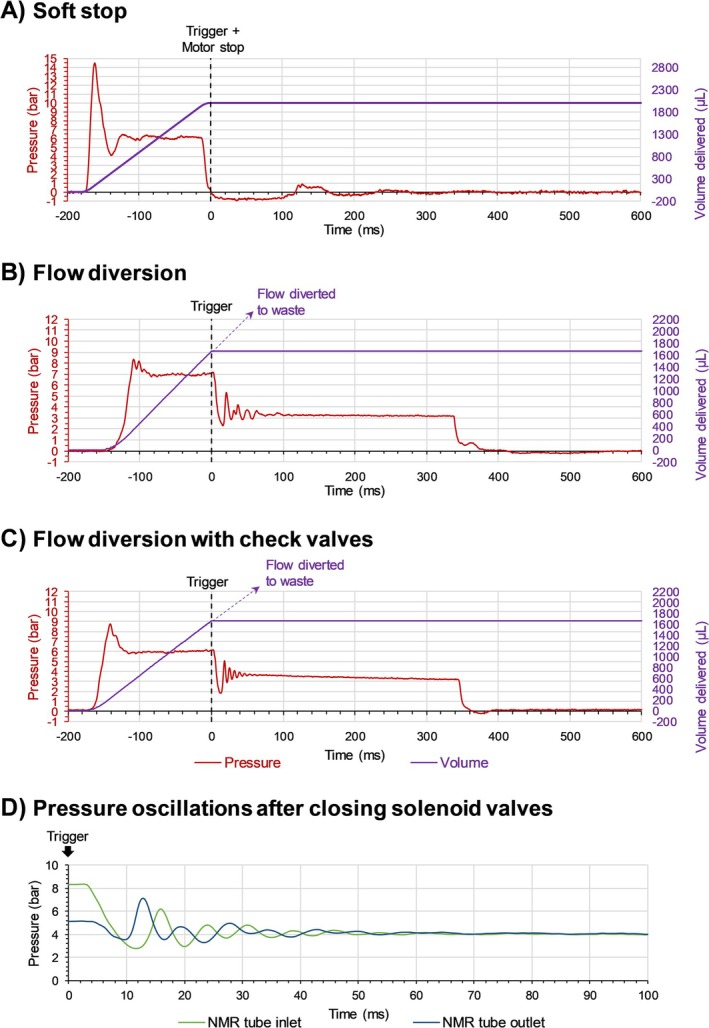
Experiments to explore the variations in the pressure, y‐axis, inside the SF‐NMR‐V2 flow tube during the injection of reagents at a combined flow rate of 12 mL/s, to deliver the total volume indicated on the right hand y‐axis. (A) Using a ‘soft‐stop,’ where the syringe drive motor is decelerated, and the flow of reagents in the NMR cell slows naturally due to friction with the tubing walls. (B) Using a ‘hard‐stop’ where solenoid valves are used to divert the incoming reagent flow and simultaneously close the waste outlet from the NMR tube. The motor is then smoothly decelerated, independently from the flow in the NMR tube. (C) Using the same ‘hard‐stop’ method, but with the addition of one‐way check valves to the inlet and outlet flow paths of the NMR tube, to damp out pressure oscillations in the tube more rapidly. The one‐way check valves increase the back‐pressure, and the flowrate was reduced to 10 mL/s to ensure the pressure limits of the flow diversion valves were not exceeded. (D) The pressure oscillations, measured at the inlet and outlet of the flow cell, after flow diversion with check valves. The maxima and minima are out of phase by approximately 3 milliseconds.

Experimentally, the ‘soft stop’ applied in SF‐NMR‐V1 is complete in around 30 milliseconds, resulting in an overall dead time of 150 milliseconds when combined with the preceding 120 millisecond transport time (see Figure 2). Applying the same soft‐stop approach with the NMR flow cell system developed for SF‐NMR‐V2 (Figure 3C), it takes a minimum of 16 milliseconds to stop the stepper motor drive from a flow rate of 12 mL/s. This is then followed by a period of flow instability with slow oscillations lasting up to 500 milliseconds (Figure 6A). A more controlled ‘hard stop’ is clearly desirable; however, it is challenging to achieve because of the need to decelerate the stepper motor drive. A flow diversion scheme was devised to chronologically separate the ‘hard stop’ and the stepper motor deceleration. Once the flow in the NMR tube has reached steady‐state, four fast‐acting (3‐millisecond) solenoid valves, simultaneously activated by a voltage trigger, divert the two inlet flow streams away from the NMR tube and close the outlet that vents the waste from the flow cell. The flow in the NMR tube is thus arrested abruptly (Figure 6B) while also allowing the stepper motor drive to be gently and independently decelerated. This approach decouples the time point at which the flow in the NMR tube is abruptly stopped from a slower and controlled deceleration of the stepper motor drive with the residual flow going to waste. The same voltage trigger is also used to initiate the NMR data acquisition pulse sequence, with predefined delays; see hereinafter.

Because of the strong magnetic field, it was not possible to house the four solenoid flow diversion valves within the magnet bore, close to the flow cell. Instead, they were located at the manifold connecting the inlet and outlet tubes to the stepper motor syringe drive. During the design phase, rapidly responding pressure transducers were also temporarily installed in the flow path at each of the valve locations to monitor the effects of the new hard stop method. As anticipated, the relatively long path length between the inlet and outlet solenoid valves results in some oscillating pressure waves rebounding between the solenoid valves and thus travelling up and down through the NMR flow cell (Figure 6B).

**FIGURE 7:**
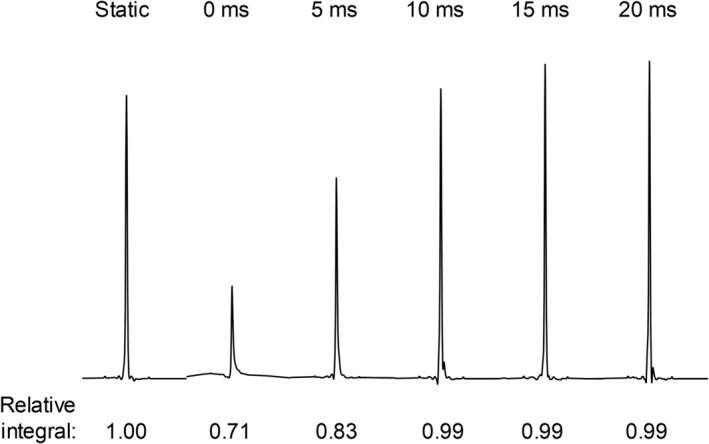
Variation in signal intensity of the CH_3_ peak of methanol injected into the SF‐NMR‐V2 flow cell (600 μL injection volume, 8 mL/s) across a series of delay times after the voltage trigger to initiate a hard stop (Figure 6C). Distortions in the baseline with delays < 10 milliseconds are attributed to residual sample movement into and out of the active volume. Relative peak integrals measured with an integration region of 3.1 ± 0.2 ppm.

Achieving a completely stationary sample is particularly important for NMR measurements, since movement of the sample within the detection volume results in corruption of the FID through Doppler and other flow effects [20, 21, 22]. For example, if the NMR measurement is triggered at the same instant as the valves close, the fluid is still moving at close to the maximum velocity, resulting in severe broadening of the peak (Figure 7, 0‐millisecond delay). Similar issues were encountered in early stopped‐flow NMR instruments that apply a classic stopping syringe, with the oscillations being deliberately damped by using stainless‐steel tubing [7]. Whilst effective in a custom‐built flow probe, this approach makes inserting a SF‐NMR instrument into a standard spectrometer very challenging. An alternative solution was thus sought. The addition of nonmagnetic one‐way check valves to the flow paths immediately before and after the NMR flow cell results in an increased oscillation frequency and decreased intensity of the pressure waves (Figure 6C,D), thus reducing the time taken for sample stabilisation.

In the experiments shown in Figure 6B,C, the trigger is sent after approximately 1600 μL of solution has been delivered, with the diversion valves receiving a second trigger 0.3 s after the stepper‐motor stops. This resets the diversion valves to their original positions and releases the 3–4 bar pressure contained in the system. In the final implementation of the system to SF‐NMR, the first trigger is sent after approximately 600 μL of nascent reaction has been delivered to ensure maximum premagnetisation; see hereinafter. The second trigger to release the pressure is sent 10 s after the flow diversion to ensure that the FID acquisition is complete before depressurisation, as the latter restores unwanted sample movement within the cell. Although the check valves do not damp out the pressure oscillations entirely (Figure 6C,D), they do sufficiently reduce the residual flow from pressure equalisation for the NMR signal observed from a pulse applied after a short delay being only slightly broader than a static sample (Figure 7). It should be noted that the addition of the stepper motor drive to the top of the shielded magnet causes a substantial change in the field homogeneity, see the Supplementary Information. However, once the probe has been reshimmed, using standard spectrometer automated processes, the field homogeneity is restored and is stable throughout repeated stopped‐flow runs. In other words, changes in position of the syringe‐drive push plate, the syringes, and the flow diversion solenoids have negligible impact on the line‐shape, see the Supplementary Information. Overall, with the flow‐diversion hard stop and nonreturn valves, the flow in the NMR tube is arrested within approximately 10 milliseconds and NMR spectra can be acquired with signal intensities and resolution fully suitable for concentration measurements and thus kinetic studies (Figure 7).

### 2.5 Premagnetisation

For an NMR signal to be detected, the sample must be in the magnetic field for long enough to achieve partial net polarisation of the nuclear spins being excited by the pulse. For quantitative measurements, it is therefore necessary to wait until near‐equilibrium populations of spins in each energy level are achieved. Typically, a premagnetisation period of five‐fold *T*_1_^max^, the longest longitudinal relaxation time constant, is accepted as being sufficient. In a standard NMR measurement, this condition is usually fulfilled passively: The sample typically sits within the magnetic field for several tens of seconds before the first measurement. However, the rapid transport to the active volume of the NMR flow cell during a stopped‐flow experiment necessitates that the nuclei in the two reagent solutions are premagnetised before they are mixed to initiate the rapid reaction.

**FIGURE 8:**
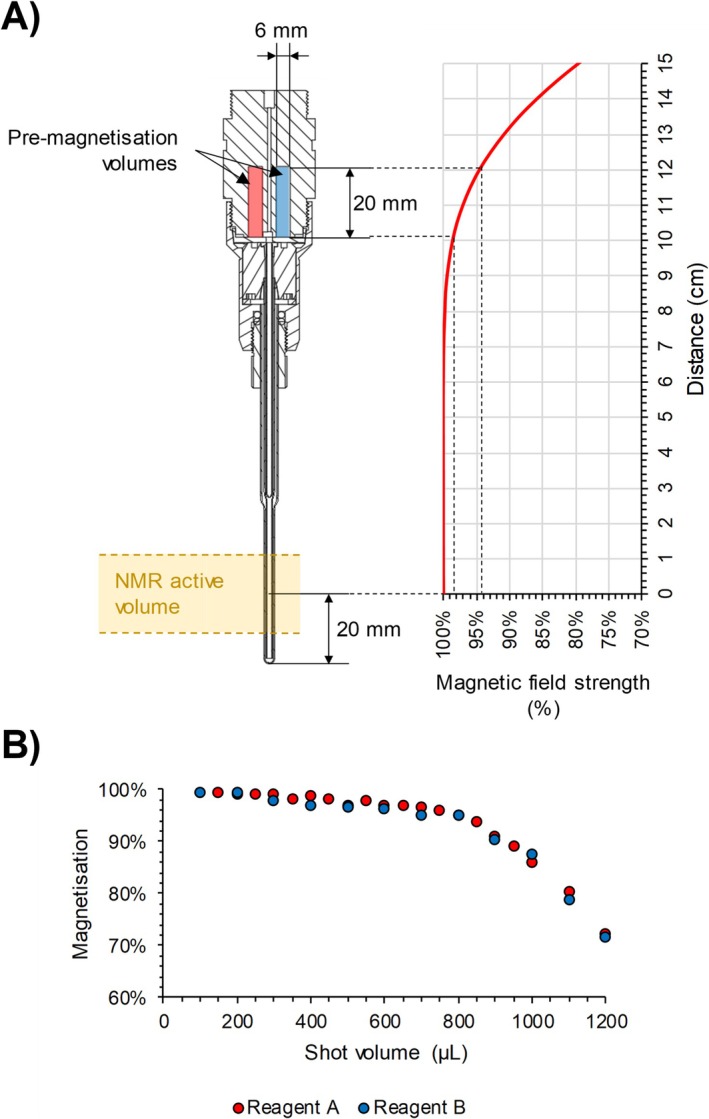
(A) Schematic showing how the magnetic field strength varies with position within the SF‐NMR‐V2 body and flow cell. (B) The change in premagnetisation as determined by the ^1^H NMR signal intensity of the CH_3_ signal of methanol when injected into the flow cell using different total shot volumes with the methanol delivered, in separate experiments, from the magnetisation volumes for reagent A (capacity 660 μL) and for reagent B (capacity 710 μL). The samples are stored in the premagnetisation volumes under static, that is, no‐flow, conditions for a time period of ≥ 5 *T*_1_^max^ before initiation of stepper‐motor syringe drive and stopped‐flow injection.

In the first‐generation SF‐NMR‐V1 instrument, premagnetisation is achieved by storing reagents in 0.5‐ to 0.7‐mL volume coils located within the body of the ‘spinner,’ immediately above the NMR tube, and within the stray field of the magnet. This results in > 90% magnetisation of the reagents before entering the NMR tube. The new design for SF‐NMR‐V2 replaces the premagnetisation coils with cylindrical volumes built into the body of the spinner (Figure 8A), this not only achieves greater premagnetisation but also has the advantage of simplifying assembly of the device. The degree of premagnetisation is determined by the residence time and position in the premagnetisation cylinder, the *T*_1_ of the nuclei, the time taken to transport the nuclei to the active volume, and the delay time, *τ*, at full field before the 90° pulse. The reagent solutions are static (motor drive off) during the premagnetisation stage. The duration of this period is set to be substantially greater than five‐fold *T*_1_ of the slowest polarising nuclei. The reagents solutions therefore reach > 99% of the maximum premagnetisation available at the positions they are located in the premagnetisation cylinders. On activation of the motor drive, the flow rates through both cylinders are equal, and the times taken to transport A and B to the active volume near‐identical. For most nuclei, the transport and stopped‐flow NMR experiment occur over a much shorter timescale than longitudinal relaxation. The system thus gives near‐quantitative signals for the most commonly monitored nuclei (^1^H, ^13^C, ^19^F, ^31^P, etc.) irrespective of their individual *T*_1_ values. Nuclei with very short *T*_1_, for example, ^11^B, may undergo some further polarisation during transport and the prepulse delay, *τ*. Any additional polarisation effects can easily be measured by control experiments and corrected for.

Because there is a longitudinal magnetic field gradient over the premagnetisation volume, the level of polarisation of the sample entering the active volume varies inversely with the volume of sample delivered. Reagents at the bottom of the premagnetisation volume (closer to the centre of the magnet) are exposed to > 98% of the magnetic field, while those at the top of the premagnetisation volume only experience 94% of the field [23]. Because the reagents at the bottom of the premagnetisation volumes enter the NMR tube first, higher levels of premagnetisation are observed for smaller injection volumes. The total volume of premagnetised sample (including unmixed reagents in the capillary tubes) is around 660 μL for reagent A and 710 μL for reagent B. Once the premagnetisation volume is depleted, the incoming nuclei have not been exposed to the full magnetic field for long enough to develop the required levels of polarisation and a progressive decrease in signal is observed (Figure 8B). The volume of the NMR flow cell is 208 μL, and a combined injection volume of 600 μL is therefore sufficient to three‐fold flush out the previous cell contents. This ultimately results in a steady‐state flow of nascent reaction through the active volume with > 96% premagnetisation, immediately before the hard stop.

### 2.6 Temperature Regulation

Accurate and stable temperature control is important for kinetic measurements, especially those with a large positive or negative enthalpy of activation where small temperature variations result in significant changes in reaction rate. Temperature regulation in SF‐NMR‐V2 is achieved by a thermostat fluid circulated through the umbilical containing the tubing for reagent delivery, solvent flush, waste flow and nonreturn valves. A low viscosity and low heat‐capacity silicone oil was found to be ideal, allowing much faster change of the system to a new temperature than the ethylene glycol/water mixture used in SF‐NMR‐V1 [1, 2]. The circulating silicone oil is actively controlled by the thermostat system. This uses a Pt100 probe located close to the terminus of inlet flow stream of silicone oil and immediately above the premagnetisation block (Figure 5A) as the reference point for the target temperature. The temperature and flow rate of the silicone oil exiting the thermostat reservoir to enter the umbilical at the valve manifold is continuously optimised to maintain the Pt100 probe at the temperature set point. The reagent solutions within the premagnetisation volumes reach thermostability in significantly less time than is taken for shimming prior to making the SF‐NMR measurements. This ensures that the solutions held in the premagnetisation volumes are at the same temperature (±0.5 K) as the NMR flow cell and its contents. The temperature of the NMR flow cell is regulated by a flow of heated/cooled nitrogen gas, controlled by the spectrometer. Our previous studies have demonstrated that the small sample volumes and large surface‐contact inherent with this method provides sufficient temperature control for most reactions, unless there is a strong endo‐ or exo‐therm [2].

### 2.7 Data Acquisition

For the NMR study of fast irreversible reactions, that is, those taking place on a 10‐ to 500‐millisecond timescale, the choice of pulse sequence and mode of data acquisition is important. Pulse sequence elements with long durations such as shaped pulses (tens of millisecond duration) or mixing times (hundreds of milliseconds) cannot be used, because the reaction will have progressed significantly during this time. This restricts the pulse sequence to short, hard pulses on the microsecond timescale. Moreover, the majority of the NMR‐active nuclei employed for reaction monitoring (^1^H, ^13^C, ^19^F, ^31^P, etc.) will not recover any significant longitudinal repolarisation before the reaction reaches completion, and the collection of multiscan FIDs, or multiple single scan FIDs is not possible for the sample present in the active volume. Instead, a single 90° pulse is employed for read‐out to maximise the observed signal [24].

**FIGURE 9:**
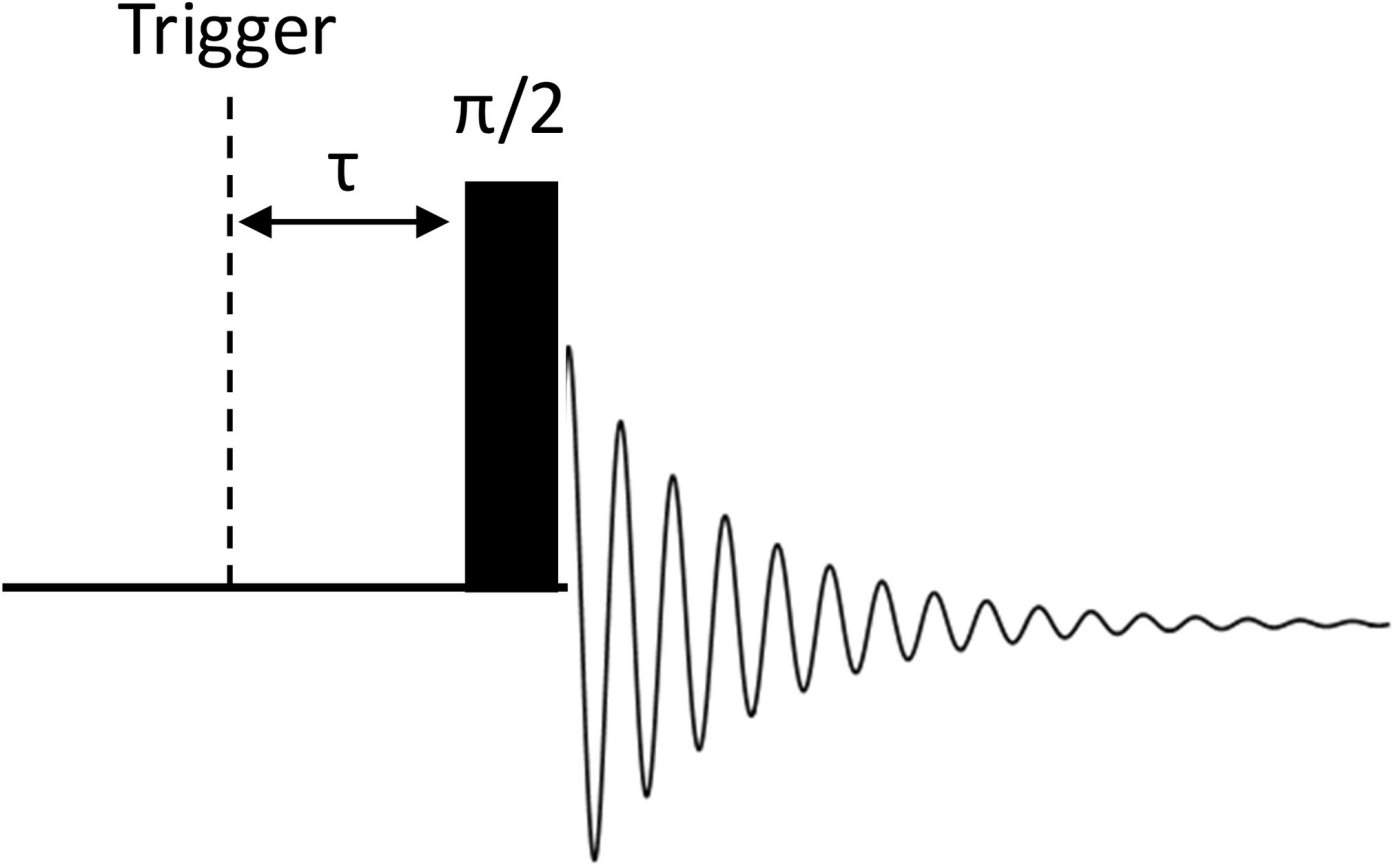
Pulse sequence used for SF‐NMR‐V2 measurements. A variable delay, *τ*, is used to offset the start of the acquisition from the point at which the trigger signal is received from the SF‐NMR‐V2 instrument. The pulse and FID acquisition duration are those of a conventional 1D NMR experiment, and the spectrum generated by Fourier transform of the entire FID, with apodisation, phasing, baseline correction and integration performed by standard procedures.

Because the reaction reaches completion in less time than it takes to acquire the FID, care must be taken to minimise any undesired delays prior to the acquisition. For example, standard Bruker pulse sequences include a 30‐millisecond delay to allow for the preceding data to be written to disk. With each SF‐NMR FID being acquired from a single scan, this additional interscan delay is not necessary as there is time for the data to be written to disk after the signal decay is complete. The default 30‐millisecond delay is therefore removed and replaced with a variable delay, *τ*, inserted between the spectrometer receiving a trigger from the SF‐NMR‐V2 instrument and the 90° pulse. This allows the start of the FID acquisition to be offset from the trigger time in a systematic manner (Figure 9). The acquisition is performed using a conventional length single scan FID collected after a 90° pulse, with the sequence triggered by the same +5 V signal from the SF‐NMR‐V2 instrument that actuates the flow‐diversion valves (Figures 5 and 6). As the reagents are premagnetised prior to injection into the NMR tube, there is no need for an interscan relaxation delay in the conventional sense, provided that sufficient time is allowed for polarisation build‐up in the premagnetisation volumes prior to the next injection.

Although the reaction reaches completion in less time than it takes to acquire the FID, the concentrations of all species are encoded into the first data point. The speciation is deconvoluted by Fourier transform of the whole FID, which is thus acquired over a sufficient period to capture the full decay of signal during *T*_2_* relaxation. The integrals in the standard Fourier transformed spectrum thus represent only the concentrations of species present at the point of application of the 90° pulse and the time‐point corresponding to the start of the FID acquisition [25]. The chemical reaction(s) causing the temporal decay of the concentration of the reactant(s) results in their NMR emission(s) decaying faster than their *T*_2_* relaxation. This results in broadening of reactant peaks in the spectrum obtained after FT. Conversely, the product peaks remain sharp, even though theoretical simulations of the spin‐system evolution suggest that a phase distortion may be expected for certain combinations of peak position and reaction rates, resulting from the product peaks inheriting the phase of the reagents [7, 25, 26, 27]. These phase distortions are not present in the NMR spectra acquired using SF‐NMR‐V2 on a 400‐MHz spectrometer, because reactions that are fast enough to induce these phase distortions are largely complete within the dead time.

**FIGURE 10:**
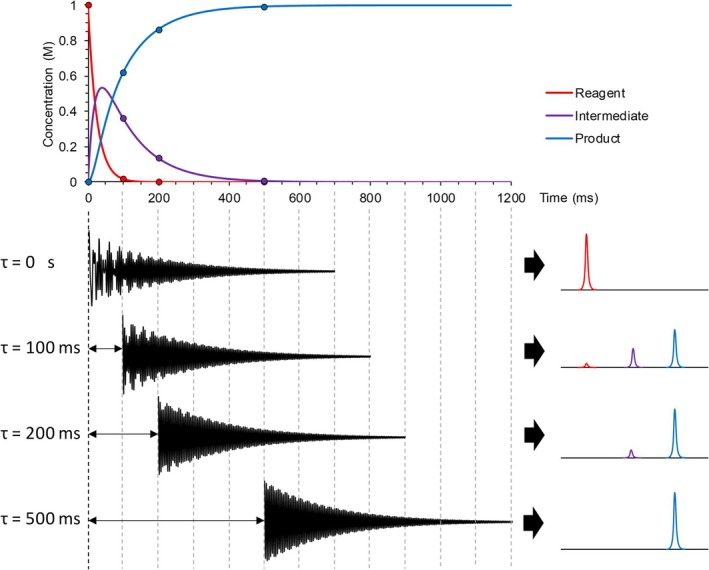
Simulated reaction kinetics and free induction decays for a first‐order reaction, proceeding via an intermediate, at different time offsets (*τ*, see Figure 9) between initiating the reaction and the start of the FID acquisition. Initial concentration = 1 M, *k*_1_ = 40 s^−1^, *k*_2_ = 10 s^−1^, *T*_2_ = 0.2 s. The integrals in the Fourier transformed spectra represent the concentrations at the first point in the single scan FID acquisition, that is, the time of application of the 90° pulse.

Because the reaction proceeds to completion during the NMR acquisition, it is possible to extract kinetic information from individual FIDs [28]. However, the kinetics employed for validation of the SF‐NMR‐V2 system are derived from a series of 1D NMR spectra obtained in the conventional manner by Fourier transform of FIDs acquired across a distribution of time points in the reaction [25]. To achieve this, the reaction is repeated, using fresh injections of reagents in the stopped flow instrument, with the delay, *τ*, between the spectrometer receiving the trigger signal and the 90° pulse being systematically incremented. This results in a series of single scan spectra acquired at increasing time offsets from the start of the reaction (Figure 10). The temporal concentration profile used for extraction of kinetic relationships for the reaction can then be derived by integration of the series of NMR spectra [29], using (*τ* + dead time) to provide a pseudo‐time axis.

**FIGURE 11:**
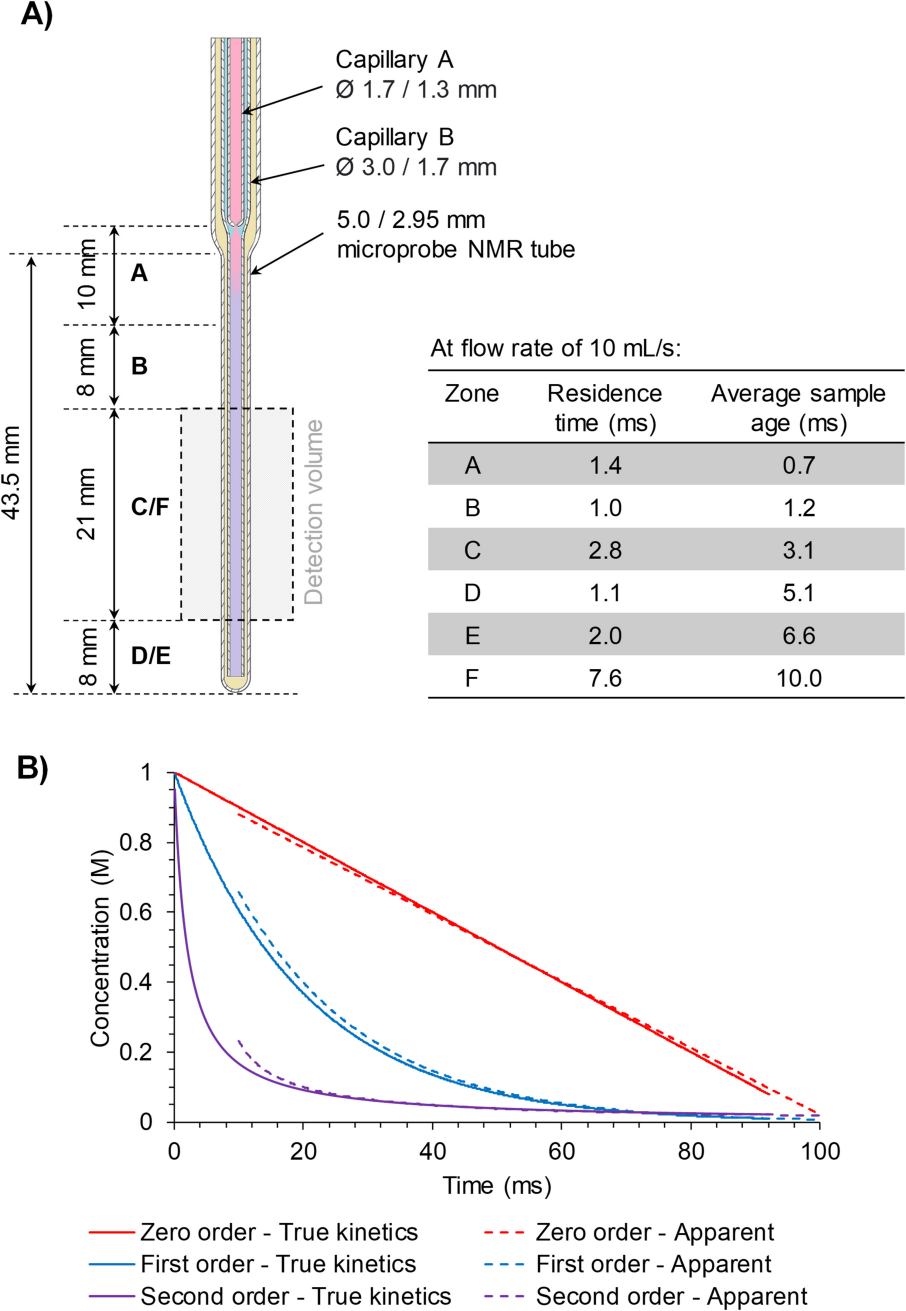
(A) Zones used for the calculation of sample age at different locations in the NMR tube. (B) Simulated reaction kinetics for zero‐, first‐ and second‐order reactions occurring in the SF‐NMR‐V2 flow cell. 1 M starting concentration, zero‐order rate constant = 10 M s^−1^, first‐order rate constant = 50 s^−1^, second‐order rate constant = 500 M^−1^ s^−1^. Kinetics simulated for the volume‐weighted combination of regions C (28 μL) and F (48 μL), with initial ages spanning 1.7–4.5 and 7.6–12.5 milliseconds, respectively, and each subdivided into eight temporal sections.

The age of the sample varies not only with time after the trigger signal, but also as a function of its position within the NMR flow cell, due to the path and the time taken for the nascent reaction to flow through the system [22]. The impact of this on the acquired spectra can be considered by theoretical division of the flow into six zones (Figure 11A). The reagents meet at the top of zone A, with fully homogenous mixing then completed by flowing 10.4 mm in 1.4 milliseconds. The nascent reaction then passes through zone B, before entering the NMR coil detection volume for the first time (zone C, 28 μL volume). The flow then continues through zone D to rebound from the bottom of the tube and pass upwards through the annular space between the capillary and NMR tube wall, zone E. The nascent reaction then passes through the NMR coil detection volume for a second time, this time on the outer side of the capillary (zone F, 48 μL volume) before exiting the top of the NMR flow cell. The signals observed in the NMR spectrum are thus a volume‐weighted average of zones C and F, which have average sample ages of 3.1 and 10.0 milliseconds, respectively.

For reactions that take place on the tens of milliseconds timescale, the difference in sample age across the detection volume has the potential to alter the observed kinetics, because the reaction will have progressed to two different extents when it receives the 90° pulse. To study this effect, kinetic simulations were performed for zero‐, first‐ and second‐order reactions. Zones C and F were each subdivided vertical into eight identical sections, with reaction progress calculated for each section separately, before a weighted average of the 16 sections (in C plus F) was taken to estimate the overall speciation that will be observed in the NMR spectrum. Each simulation started at 1 M concentration, reacting via zero‐, first‐ and second‐order kinetics with rate coefficients selected so that the reactions reach 98%–100% conversion over a period of 100 milliseconds (Figure 11B). Small deviations from the true kinetics were observed in the simulations in all cases. The observed zero‐order rate coefficient is *k*_obs_ = 9.5 M s^−1^, 5.0% below the true value of 10 M s^−1^. The observed first‐order rate coefficient, *k*_obs_ = 50 s^−1^, is identical to the true value; however, the kinetics are offset by a time constant, more easily visualised in the linearised semilog plot (see Supporting Information). The observed second order rate coefficient, *k*_obs_ = 528 M^−1^ s^−1^, is 5.6% above the true value of 500 M^−1^ s^−1^. The effect of the sample age diminishes for slower reactions. However, for fast reactions, the deviations between true and observed rate coefficient(s) for proposed mechanism(s) should be explored. This can be readily achieved for more complex process than the simple zero‐, first‐ and second order ones noted above, by numerical methods simulations [29] of the kinetics of the process (es) occurring concurrently in zones C and F.

## 3 Results and Discussion

With the design and construction of SF‐NMR‐V2 complete, and following the validation of flow, mixing, stopping, pressure oscillation, premagnetisation, NMR line‐shape, temperature control and kinetic methodology, as described in the Materials and Methods section above, we used two examples of rapid, homogenous, mixing‐induced, chemical reactions to probe the scope and limitations of the system.

### 3.1 Rapid Hydrolysis of Methyl Formate

In 1979, Ernst reported the study of transient reaction kinetics of the base‐mediated deuterolysis of methyl formate in D_2_O solution using a custom built stopped‐flow NMR probe and spectrometer [7]. This reaction proceeds rapidly at room temperature, reaching completion in under 100 milliseconds. Ernst estimated the second‐order rate coefficient to be 80 M^−1^ s^−1^ using lineshape fitting of single ^1^H NMR spectra acquired via a pulse applied 3 milliseconds after initiating the reaction [7].

**SCHEME 1:**
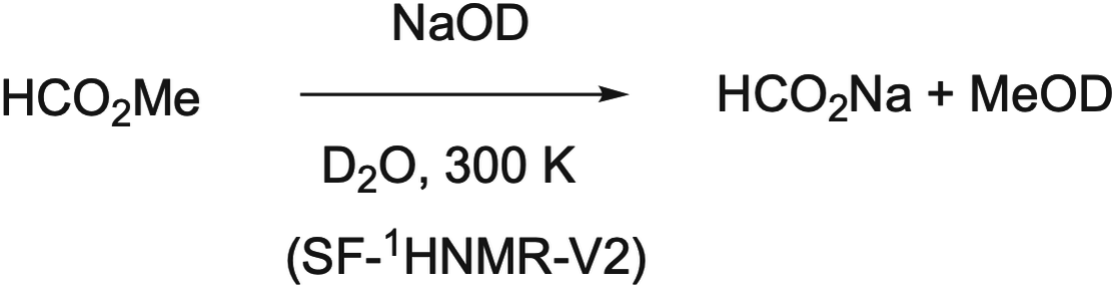
The base‐mediated deuterolysis of methyl formate, used to test the capability and reproducibility of the SF‐NMR‐V2 instrument by ^1^H NMR spectroscopy.

**FIGURE 12:**
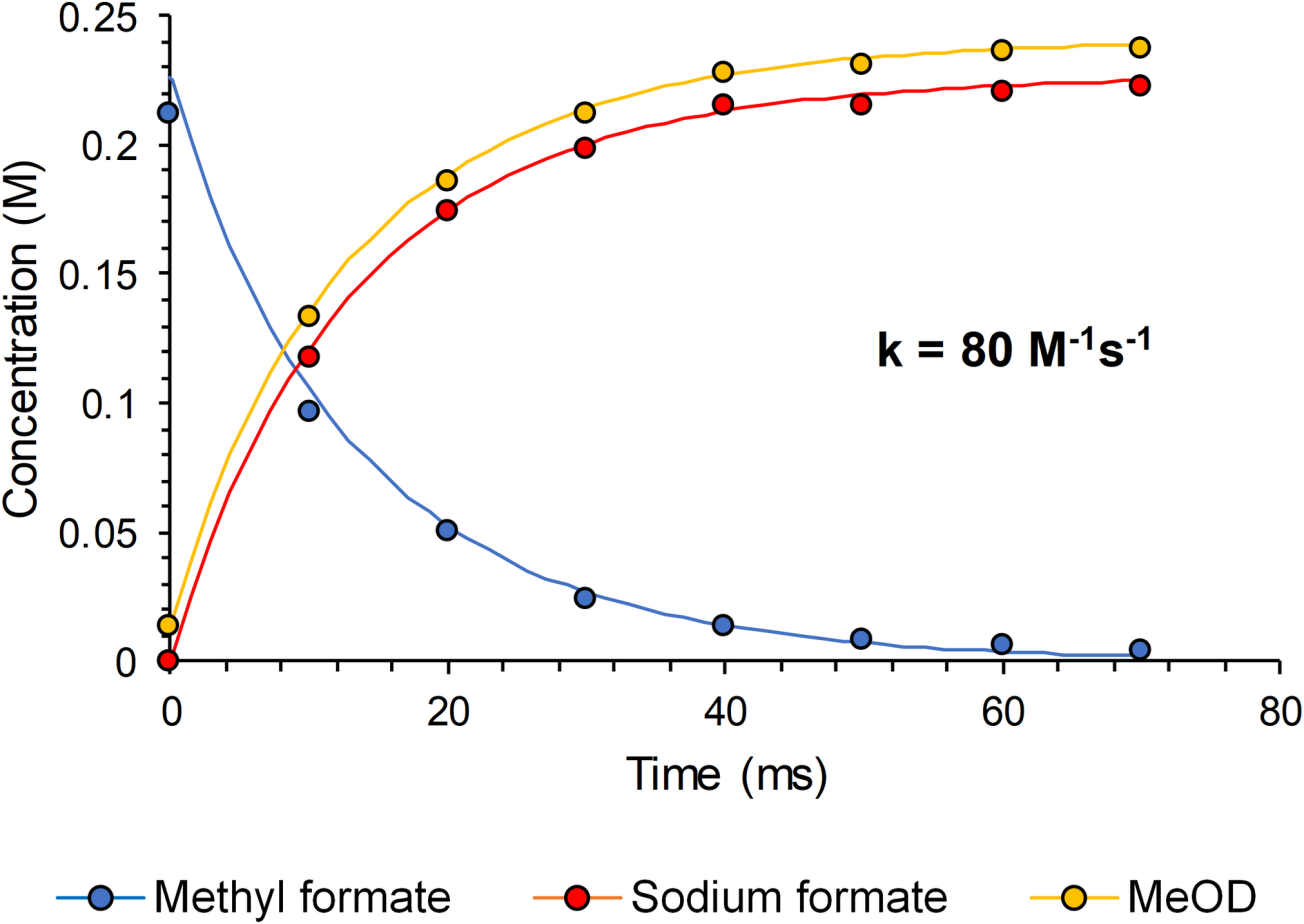
Reaction kinetics for the hydrolysis of methyl formate determined by ^1^H NMR spectroscopy using SF‐NMR‐V2, and integrating the methyl signals in MeOD and HCO_2_Me. Conditions: 0.25 M methyl formate, 1.03 M NaOD in D_2_O, 27°C, 600 μL injection volume, 10 mL/s. The x‐axis is a pseudo time‐scale with each data point obtained from a separate injection of reagents and a cumulative 10‐millisecond time increment prior to application of the 90° pulse/FID acquisition. The simulation (solid lines) employs a phenomenological dead time of 10 milliseconds between the trigger signal and the cessation of flow in the active volume (zones C and F, Figure 9). The stock solution of methyl formate in D_2_O undergoes slow neutral hydrolysis on storage, leading to some MeOD being present at *t* = 0.

We have employed the same pseudo first‐order reaction conditions (Scheme 1, excess base) to test the SF‐NMR‐V2 instrument (Figure 12). With the longer dead time and higher field spectrometer (10 milliseconds, 400 MHz) than the custom stopped‐flow probe used by Ernst (3 milliseconds, 90 MHz) [7], there are no phase distortions observed in the product ^1^H NMR signals [25]. A series of data points were acquired, each with a fresh injection of reagents, and the delay before the start of the acquisition extended each time in 10 millisecond increments. Concentrations of MeOD and methyl formate were calculated at each time point, using the integrals of the well‐resolved methyl signals, and plotted against sample age (dead‐time plus delay, *τ*, before acquisition). Repeat measurements were carried out for 10‐, 20‐ and 30‐millisecond time points and averaged. There was less than 6% difference between repeat measurements in all cases except for the sodium formate ^1^H signal in the data collected at 10 milliseconds, where overlap with the signal from the formyl proton in the methyl formate results in integration errors. Fitting of the data using a standard numerical kinetic model gave a second‐order rate coefficient of 80 M^−1^ s^−1^.

### 3.2 Rapid Protodeboronation of Pentafluorophenyl Trihydroxy Boronate

Protodeboronation results in the decomposition of organoboron reagents, R‐BX_2_ to form R‐H and HO‐BX_2_, and in most cases [30], is base catalysed. For arylboronic acids, the protodeboronation rate is strongly accelerated by electron‐withdrawing aryl ring substituents [31, 32].

Pentafluorophenyl boronic acid is one of the most base‐sensitive examples, and at high pH, we have previously estimated the half‐life of the corresponding boronate to be in the range of 3 milliseconds at 70°C [32]. The latter measurement required the application of a rapid quench‐flow method where the reagents pass through two sequential passive mixers, with a short transport time between them. The first mixer combines the reactants to initiate the reaction, and the second quenches the reaction by introduction of a powerful inhibitor. The halted reaction sample is then analysed by off‐line NMR spectroscopy. The technique is laborious, the quench‐flow instrument requires frequent recalibration and consumes large volumes of reactant solutions.

**SCHEME 2:**
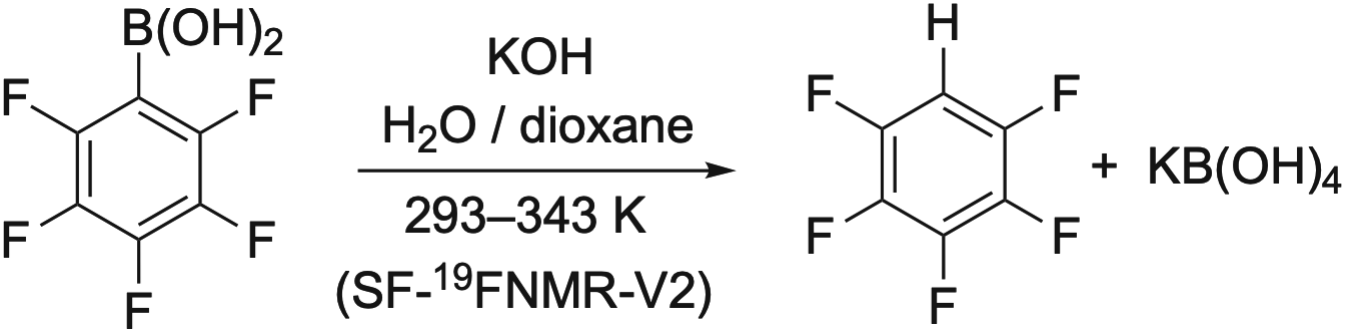
Base‐mediated protodeboronation of pentafluorophenyl boronic acid, used to test the capability and reproducibility of the SF‐NMR‐V2 instrument by ^19^F NMR spectroscopy.

**FIGURE 13:**
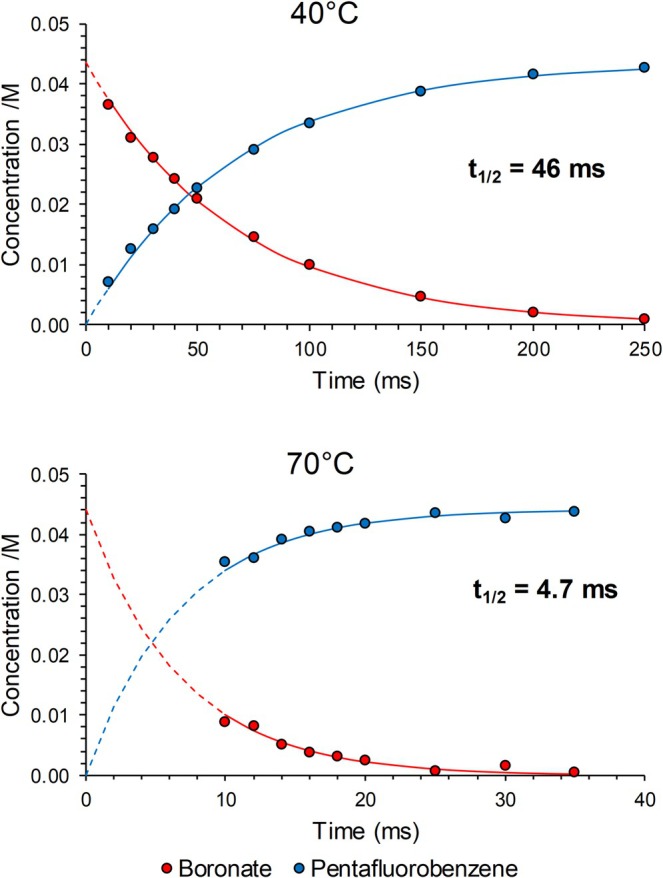
Kinetics of the protodeboronation of potassium pentafluorophenyltrihydroxy boronate at 40°C and at 70°C, determined by ^19^F NMR spectroscopy using the SF‐NMR‐V2 instrument. Conditions: 0.05 M boronic acid, 0.16 M potassium hydroxide in 1:1 dioxane:water mixture, 600 μL injection volume, 10 mL/s. The x‐axis is a pseudo time‐scale with each data point obtained from a separate injection of reagents with cumulative 10‐, 25‐ and 50‐ (at 40°C) and 2‐ and 5‐millisecond (at 70°C) time increments prior to application of the 90° pulse/FID acquisition. The averaged data for the ortho, meta and para signals were normalised for constant mass balance prior to fitting.

The protodeboronation reaction, Scheme 2, was used to test our first‐generation stopped flow NMR instrument, SF‐NMR‐V1, with reaction half‐lives found to be 117 milliseconds at 27°C, and 45 milliseconds at 40°C [2]. The 150 millisecond dead time of the SF‐NMR‐V1 instrument meant that at 40°C the reaction had already reached 90% completion before the first ^19^F NMR spectrum was acquired, reducing the accuracy of the kinetic modelling. With a phenomenological dead time of approximately 10 milliseconds, the temporal span and data density that can be acquired are substantially enhanced with SF‐NMR‐V2 (Figure 13), with each time point acquired using a separate reaction to generate a pseudo time axis.

Increasing the temperature to 70°C probes the limits of SF‐NMR‐V2, with the protodeboronation reaction having an estimated half‐life of 4.7 milliseconds (Figure 13). The reaction reaches > 99.7% completion in approximately 35 milliseconds, and the substrate would not be detected in any previous generation SF‐NMR instrument designed for use on a standard NMR spectrometer and probe. There is relatively little scatter in the data, highlighting the reproducibility of the mixing and timing of the reagent injections, flow diversions, efficacy of nonreturn valve damping of pressure oscillations and the accuracy of the incremented delay times before application of the 90° pulses.

**FIGURE 14:**
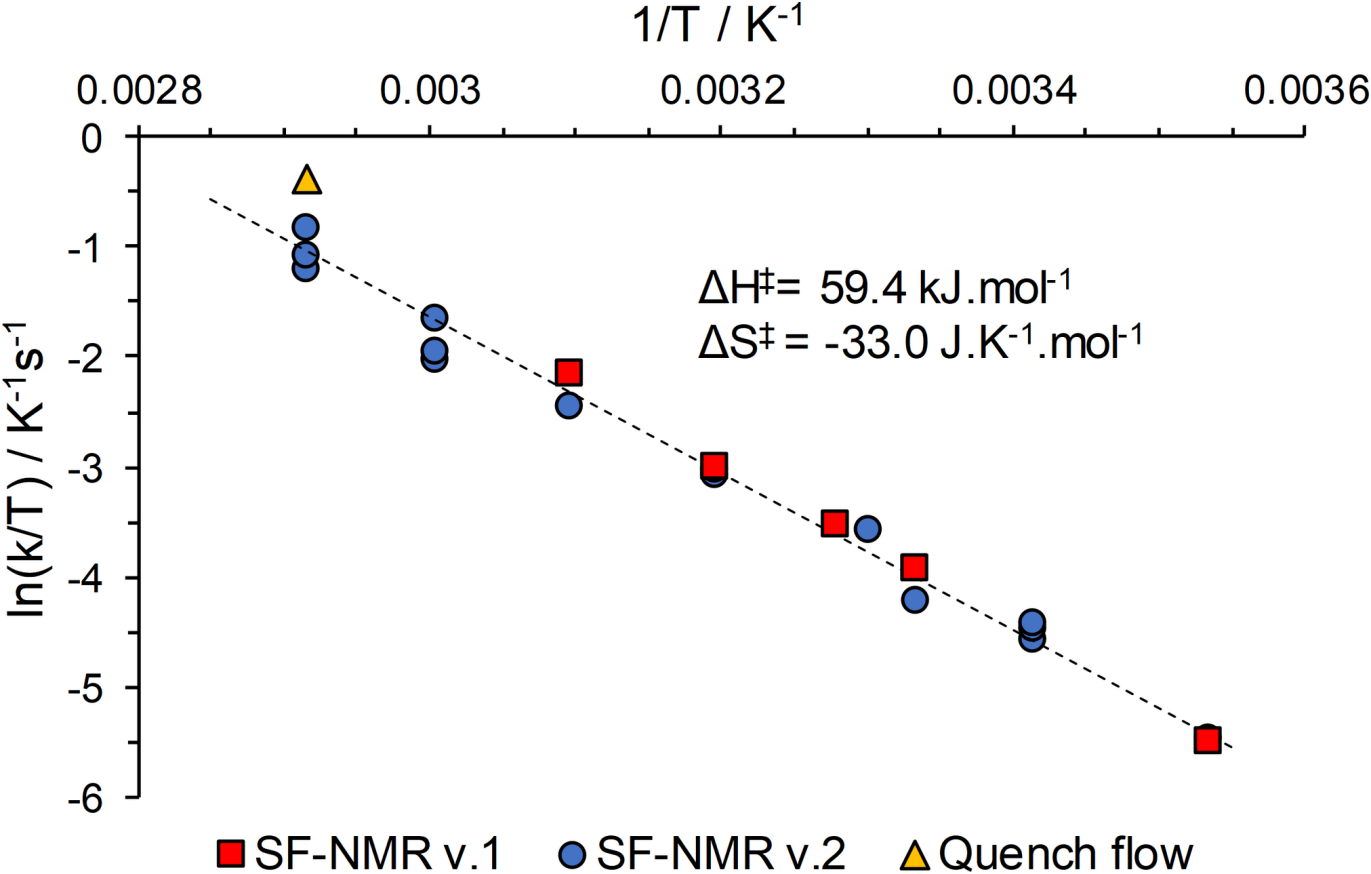
Eyring analysis of activation parameters for the base‐mediated protodeboronation of pentafluorophenyl boronic acid, comparing data acquired using the first generation stopped‐flow NMR instrument, SF‐NMR‐V1 [1, 2], the instrument presented herein, SF‐NMR‐V2 and kinetics measured using a rapid‐quench flow instrument, followed by off‐line NMR analysis [31, 32]. The dashed line is the best fit obtained by standard linear regression of all 20 datapoints.

The data acquired using the SF‐NMR‐V2 instrument design can be compared with that acquired using the previous SF‐NMR‐V1 instrument [2] and the rapid quench‐flow measurement [32]. The Eyring plot (Figure 14) shows agreement between the measurements from different instruments and between repeat measurements, demonstrating a high degree of control over reaction assembly, timing and temperature using SF‐NMR‐V2.

## 4 Conclusions

A new stopped‐flow NMR instrument, referred to herein as SF‐NMR‐V2, has been developed. It is based on a stepper motor syringe drive delivering two reagent streams into a rapid mixing NMR insert flow cell. The external profile of the insert is the same as a standard NMR spinner, allowing use on an unmodified NMR spectrometer and probe. While a limitation of using a single stepper motor is that both syringes deliver the same volume of reagents, development work is ongoing to incorporate the operational advantages of multiple independent syringe drives with the high flow rates needed for the short dead times achieved with the new instrument. Within the NMR insert, the reagents are stored in premagnetization volumes close to the magnet isocentre, with a residence time ≥ 5 *T*_1_, enabling them to build up maximum and equal polarization before injection into the NMR tube. The flow lines and premagnetization volumes are thermostated using silicone oil controlled by an external circulator. The temperature at the premagnetization stage is matched to that of the spectrometer‐controlled gas flow around the active volume and flow cell. Once injected, the reagents mix passively through custom‐shaped concentric glass capillary tubes, with complete mixing taking 1.4 milliseconds. The nascent reaction flows down and then up through the NMR detection volume before exiting at the top of the flow cell to a waste reservoir located outside the magnet. Before making an NMR measurement, the inlet and outlet flows are rapidly stopped using a set of fast‐acting (< 3 millisecond) diversion valves located on the syringe drive. Residual flow and pressure spikes are damped out using one‐way check valves located close to the NMR tube, allowing a usable NMR spectrum to be acquired approximately 10 milliseconds after initiation of the flow diversion valves. Back‐mixing of reactants occurs in a small section (≤ 3 mm) above the reagent inlet capillaries. To ensure this back‐mixing does not interfere with the rapid reaction that is analyzed by NMR spectroscopy, the location of the mixer was set to leave an 8‐mm gap between the position of complete mixing and entry into the active volume.

To obtain maximum signal to noise, each NMR spectrum is acquired using a single 90° pulse [24], where the concentration of reaction components is defined by the first point in the FID [25], with their spectral resolution and quantitative speciation extracted by Fourier transform of the standard 1D NMR FID. The process is repeated several times, incrementing the delay between triggering the valves and the 90° pulse FID acquisition to build a kinetic plot that has a pseudo time scale corresponding to the known dead‐time plus the incremented delay prior to the 90° pulse [1, 2, 25]. The high flow rate and small volume result in the relative age of the sample, held within the flow path through the annular spaces between the concentric capillaries, spanning 13.3 milliseconds. Two subvolumes in this flow path, containing 28 and 48 μL of the nascent reaction, are located within the NMR detection volume, with average sample ages of 3 and 10 milliseconds, respectively, at the stopping time. Despite this temporal blurring, there are only relatively small deviations between the observed reaction rates and the true kinetics of the system being studied. Simulations of these conditions show that the effects are greater for zero‐ and second‐order reactions, with deviations in the estimated rate coefficients being approximately −5% and +5%, respectively, from the true values, for reactions that proceed to completion in 100 milliseconds. For first‐order reactions, the correct rate coefficients are obtained, albeit with a small time‐offset in the temporal concentration profile, and thus employment of pseudo first order conditions for higher‐order reactions is beneficial, if possible.

The capabilities and limitations of the SF‐NMR‐V2 instrument have been tested using two reactions: the base‐mediated hydrolysis of methyl formate, monitored by 400 MHz ^1^H NMR spectroscopy at 27°C, and the protodeboronation of potassium pentafluorophenyltrihydroxy boronate, monitored by 380 MHz ^19^F NMR spectroscopy at temperatures up to 70°C. In both cases, there was good control of temperature, reagent mixing, sample transport and timing, minimal spectral corruption from the damped pressure oscillations and minimal data scatter between repeat experiments. The rate coefficients obtained for these second and first‐order reactions that proceed to completion in around 70 and 35 milliseconds, respectively, are in good agreement with prior literature determinations and with results independently measured using IR spectroscopic and rapid quenched‐flow techniques.

**FIGURE 15:**
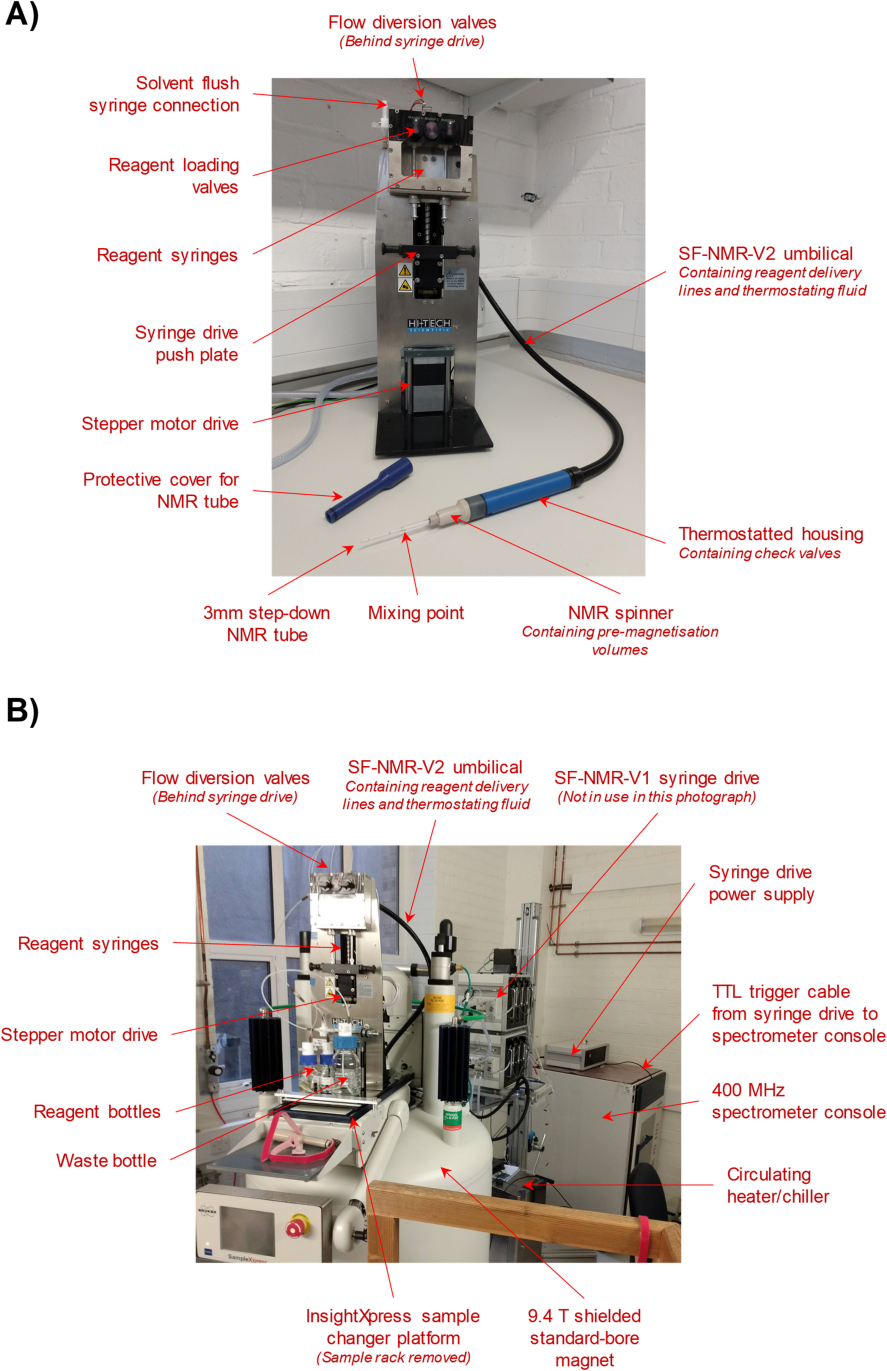
Photographs of (A) SF‐NMR‐V2 instrument (excluding syringe drive power supply) and (B) SF‐NMR‐V2 instrument in operation with the motor‐drive and syringe assembly installed on top of the SampleXpress platform, with sample case removed, and the umbilical and stopped‐flow cell inserted into the magnet bore. Key components labelled. In addition, shown at the rear of the photo is the prior first stopped‐flow NMR system (SF‐NMR‐V1) [1, 2], commercialised as InsightXpress [18, 19]. Further photographs of SF‐NMR‐V2 are available in the supporting Information.

In conclusion, the SF‐NMR‐V2 instrument (Figure 15) can be used to study reactions with half‐lives as short as 5 milliseconds. Unlike previous custom‐built probe instruments that operate on these timescales, SF‐NMR‐V2 is compatible with standard NMR spectrometers and probes. Taking around 20 min to install ready for reaction monitoring, and a similar time to fully deinstall from the spectrometer after use, the new instrument requires no permanent spectrometer or console modifications. The syringe motor drive can be located on any suitable stable platform. We are currently using the base of a SampleXpress sample changer with the sample carousel removed and a clamping system added to hold the stepper motor firmly in place. However, alternative bespoke lifting platforms, for example, that used for SF‐NMR‐V1 (Figure 15B), could also be employed on non‐SampleXpress equipped spectrometers. While the large stepper motor, its housing and the syringe drive (Figure 15A) cause a substantial change to the magnetic field homogeneity, the probe is readily reshimmed after installation at the top of the shielded magnet, and the field remains stable and suitably homogenous throughout the SF‐NMR‐V2 measurements. The SF‐NMR‐V2 system offers unprecedented practicality for the analysis of mixing‐induced rapid reactions by NMR spectroscopy in a shared NMR facility. The instrument is being actively applied to a range of kinetic and mechanistic investigations in our laboratories, and the results from these will be reported in due course.

## Author Contributions

**Andrew M. R. Hall:** methodology, writing – review and editing, writing – original draft, investigation, project administration, data curation, conceptualization, formal analysis. **Edward J. King:** investigation, methodology, writing – review and editing, conceptualization. **Lloyd A. L. Mitchell:** methodology, resources. **George A. Steedman:** methodology, resources. **Stuart Johnstone:** methodology, resources. **Clark Landis:** conceptualization. **Guy C. Lloyd‐Jones:** conceptualization, writing – original draft, writing – review and editing, project administration, supervision, resources, funding acquisition, investigation, methodology, formal analysis.

## Funding

This work was supported by the European Research Council (340163 and 838616) and Engineering and Physical Sciences Research Council (EP/V048384/1 and EP/W007517).

## Conflicts of Interest

The authors declare no conflicts of interest.

## Supporting information




**Figure S1:** NMR spectrum of a sample containing trifluoracetic acid (TFA), measured using SF‐NMR version 2 prototype without gold plating on the capillary tube. Separate NMR signals are observed for TFA located inside the capillary and in the outer NMR tube.
**Figure S2:** Frequency rejection profile recorded during probe tuning and matching of a sample of 1% CHCl3 in MeOD using the SF‐NMR version 2 prototype with: (a) no capillary tube (blue line), (b) capillary tube without gold plating (red line) and (c) gold‐plated capillary (green line).
**Figure S3:** NMR spectra of CHCl3 and CHD2OD signals in a sample of 1% CHCl3 in MeOD using the SF‐NMR version 2 prototype with: (a) no capillary tube (blue line), (b) capillary tube without gold plating (red line) and (c) gold‐plated capillary (green line).
**Figure S4:** Photographs of the gold‐plated capillary tube after exposure to trifluoroacetic acid, resulting in delamination of the gold plating.
**Figure S5:** Still images from high‐speed video footage (960 frames per second) overlaid with the pressure within the NMR tube. Recorded during the mixing of and ethanol solution of bromothymol blue (inner capillary) and an ethanolic solution of hydrochloric acid (outer capillary) at a combined flowrate of 8 mL/s (Note: Higher flow rates of 10 mL/s were used in the final design. This has little to no impact on the mixing efficiency or stopping time as can be observed in Figures S7, S8 and the supporting video).
**Figure S6:** Pressure oscillations in SF‐NMR‐V2 immediately following actuation of the flow diversion valves (10 mL/s). Pressure waves oscillate between the check valves on the inlet and outlet of the NMR tube, resulting in an echo‐like signal where the pressure at the inlet and outlet of the NMR tube are out of phase.
**Figure S7:** Still images from high‐speed video footage (960 frames per second) recorded during three repeats of mixing of an ethanol solution of bromothymol blue (inner capillary) and an ethanolic solution of hydrochloric acid (outer capillary) at a combined flowrate of 10 mL/s.
**Figure S8:** Still images from high‐speed video footage (960 frames per second) recorded during three repeats of mixing of an ethanol solution of bromothymol blue (outer capillary) and an ethanolic solution of hydrochloric acid (inner capillary) at a combined flowrate of 10 mL/s.
**Figure S9:** Spectra of the CHCl3 peak in a sample containing 1% CHCl3 in acetone‐d6 (standard 5‐mm NMR tube) acquired under various configurations of the SF‐NMR‐V2 instrument: (1) without the motor drive in place, (2) with the motor drive in place on top of the magnet, but with the stepper motor powered off, (3) with the motor drive in place and motor in motion, (4) with the motor stationary and the flow diversion valves actuated, (5) with the motor drive in place, after reshimming the magnet. Half‐height line widths are indicated for each peak in units of Hertz.
**Figure S10:** Concentration versus time plots for simulated reaction kinetics using the SF‐NMR insert. (A) Zero‐order reaction, rate constant = 10 M s–1. (b) First‐order reaction, rate constant = 50 s–1. (c) Second‐order reaction, rate constant = 500 M s–1. 1 M starting concentration. Kinetics simulated for the volume‐weighted combination of regions C (28 μL) and F (48 μL), with initial ages spanning 1.7–4.5 and 7.6–12.5 milliseconds, respectively, and each subdivided into eight temporal sections.
**Figure S11:** Linearised concentration vs. time plots for simulated reaction kinetics using the SF‐NMR insert. (A) Zero‐order reaction, rate constant = 10 M s–1. (b) First‐order reaction, rate constant = 50 s–1. (c) Second‐order reaction, rate constant = 500 M s–1. 1 M starting concentration. Kinetics simulated for the volume‐weighted combination of regions C (28 μL) and F (48 μL), with initial ages spanning 1.7–4.5 and 7.6–12.5 milliseconds, respectively, and each subdivided into eight temporal sections.
Scheme S1: The base‐mediated deuterolysis of methyl formate.
Scheme S2: Base‐mediated protodeboronation of pentafluorophenyl boronic acid.
**Figure S12:** Kinetics of the protodeboronation of potassium pentafluorophenyltrihydroxy boronate at 20°C, determined by 19F NMR spectroscopy using the SF‐NMR‐V2 instrument. Conditions: 0.05 M boronic acid, 0.16 M potassium hydroxide in 1:1 dioxane:water mixture, μL injection volume, 10 mL/s. The x‐axis is a pseudo time‐scale with each data point obtained from a separate injection of reagents with time increments of 10, 50, 100, 200, 300, 400, 500, 750, 1000 and 1500 milliseconds prior to application of the 90° pulse/FID acquisition.
**Figure S13:** Kinetics of the protodeboronation of potassium pentafluorophenyltrihydroxy boronate at 40°C, determined by 19F NMR spectroscopy using the SF‐NMR‐V2 instrument. Conditions: 0.05 M boronic acid, 0.16 M potassium hydroxide in 1:1 dioxane:water mixture, μL injection volume, mL/s The x‐axis is a pseudo time‐scale with each data point obtained from a separate injection of reagents with time increments of 10, 20, 30, 40, 50, 75, 100, 150, 200 and 250 milliseconds prior to application of the 90° pulse/FID acquisition.
**Figure S14:** Kinetics of the protodeboronation of potassium pentafluorophenyltrihydroxy boronate at 60°C, determined by 19F NMR spectroscopy using the SF‐NMR‐V2 instrument. Conditions: 0.05 M boronic acid, 0.16 M potassium hydroxide in 1:1 dioxane:water mixture, μL injection volume, mL/s The x‐axis is a pseudo time‐scale with each data point obtained from a separate injection of reagents with time increments of 10, 15, 20, 25, 30, 40, 50, 60, 70 and 80 milliseconds prior to application of the 90° pulse/FID acquisition.
**Figure S15:** Kinetics of the protodeboronation of potassium pentafluorophenyltrihydroxy boronate at 70°C, determined by 19F NMR spectroscopy using the SF‐NMR‐V2 instrument. Conditions: 0.05 M boronic acid, 0.16 M potassium hydroxide in 1:1 dioxane:water mixture, μL injection volume, mL/s The x‐axis is a pseudo time‐scale with each data point obtained from a separate injection of reagents with time increments of 10, 12, 14, 16, 18, 20, 25, 30 and 35 milliseconds prior to application of the 90° pulse/FID acquisition.
**Figure S16:** Cross‐section of the NMR insert at angles of 0°, 60° and 120°, showing (a) the flow path of reagent A (red) and the solvent flush (pale red), (b) the flow path of reagent B and (c) the flow path of the waste exiting the NMR tube. Reagent B and waste flow through circular galleries in the capillary block (see inset image), which enable the flow to distribute evenly around the annular space between the capillaries.
**Figure S17:** Photograph of SF‐NMR‐V2 instrument in position on top of NMR magnet, with key components labelled.
**Figure S18:** Photograph of SF‐NMR‐V2 valve manifold attached to syringe drive, with key components labelled.
**Figure S19:** Photograph of SF‐NMR‐V2 umbilical detached from syringe drive, with key components labelled.
**Figure S20:** Photograph of SF‐NMR‐V2 valve manifold detached from umbilical, with key components labelled.
**Figure S21:** Photograph of partially dismantled SF‐NMR‐V2 NMR tube insert, with key components labelled.
**Figure S22:** Two photographs of partially assembled SF‐NMR‐V2 capillary block, showing attachment of inner and outer capillaries, flow paths and with key components labelled.
**Figure S23:** Photograph of SF‐NMR‐V2 NMR tube, with key components labelled.
**Figure S24:** Photograph of SF‐NMR‐V2 premagnetisation block, showing premagnetisation volumes.
**Figure S25:** Two photographs of partially assembled SF‐NMR‐V2 NMR insert, showing reagent connections and with key components labelled.
**Figure S26:** Photograph of SF‐NMR‐V2 umbilical, with housing removed to show location of check valves, and with key components labelled.
**Figure S27:** Wiring schematic for 5 V TTL output.
**Figure S28:** Photograph of the back of the syringe drive controller, showing 5 V TTL output for valves labelled ‘S Solenoid control’

**Video S1:** Supporting Information.

## Data Availability

The data that supports the findings of this study are available in the supplementary material of this article.
