# Supplementary material for: A Stopped‐Flow Instrument for Millisecond Timescale Reaction Monitoring on a Standard NMR Spectrometer
Source: Magn Reson Chem. 2026 May 5;64(8):711–28. doi: 10.1002/mrc.70114 (PMC13327140; doi:10.1002/mrc.70114)
Supplement: Supplementary file 1 — Figure S1: NMR spectrum of a sample containing trifluoracetic acid (TFA), measured using SF‐NMR version 2 prototype without gold plating on the capillary tube. Separate NMR signals are observed for TFA located inside the capillary and in the outer NMR tube. Figure S2: Frequency rejection profile recorded during probe tuning and matching of a sample of 1% CHCl3 in MeOD using the SF‐NMR version 2 prototype with: (a) no capillary tube (blue line), (b) capillary tube without gold plating (red line) and (c) gold‐plated capillary (green line). Figure S3: NMR spectra of CHCl3 and CHD2OD signals in a sample of 1% CHCl3 in MeOD using the SF‐NMR version 2 prototype with: (a) no capillary tube (blue line), (b) capillary tube without gold plating (red line) and (c) gold‐plated capillary (green line). Figure S4: Photographs of the gold‐plated capillary tube after exposure to trifluoroacetic acid, resulting in delamination of the gold plating. Figure S5: Still images from high‐speed video footage (960 frames per second) overlaid with the pressure within the NMR tube. Recorded during the mixing of and ethanol solution of bromothymol blue (inner capillary) and an ethanolic solution of hydrochloric acid (outer capillary) at a combined flowrate of 8 mL/s (Note: Higher flow rates of 10 mL/s were used in the final design. This has little to no impact on the mixing efficiency or stopping time as can be observed in Figures S7, S8 and the supporting video). Figure S6: Pressure oscillations in SF‐NMR‐V2 immediately following actuation of the flow diversion valves (10 mL/s). Pressure waves oscillate between the check valves on the inlet and outlet of the NMR tube, resulting in an echo‐like signal where the pressure at the inlet and outlet of the NMR tube are out of phase. Figure S7: Still images from high‐speed video footage (960 frames per second) recorded during three repeats of mixing of an ethanol solution of bromothymol blue (inner capillary) and an ethanolic solution of hydrochloric a [file MRC-64-711-s001.pdf]

Supporting Information

A Stopped-Flow Instrument for Millisecond Timescale Reaction Monitoring on a Standard NMR Spectrometer

Andrew M. R. Hall,[a] Edward J. King,[b] Lloyd A. L. Mitchell,[a] George A. Steedman,[a] Stuart Johnstone,[a] Clark Landis,[c] Guy C. Lloyd-Jones*[a]

[a] School of Chemistry, The University of Edinburgh, Joseph Black Building, The King's Buildings, Edinburgh, EH9 3FJ, UK. Email: guy.lloyd-jones@ed.ac.uk
[b] TgK Scientific, 7 Longs Yard, Bradford-on-Avon, Wiltshire, BA15 1DH, UK.
[c] Department of Chemistry, University of Wisconsin, 1101 University Avenue, Madison, WI 53706, USA.

1 TABLE OF CONTENTS

2 Supplementary figures ... 3

2.1 Gold plated capillary tube ... 3

2.2 Mixing efficiency ... 6

2.3 Effect of motor drive on shimming ... 9

2.4 Simulation of the effect of the instrument dead-time on reaction kinetics ... 10

2.5 Deuterolysis of methyl formate, monitored by ^{1}H NMR. ... 12

2.6 Protodeboronation of pentafluorophenyl boronate, monitored by ^{19}F NMR. ... 13

2.7 Flow path within NMR insert ... 16

3 Photographs of SF-NMR-V2 instrument ... 17

4 NMR pulse sequence ... 25

5 Design drawings and materials ... 26

5.1.1 Design notes ... 26

5.2 Syringe drive ... 27

5.2.1 Syringe drive control script ... 28

5.3 NMR umbilical ... 30

5.3.1 List of parts ... 30

5.3.2 Engineering drawings ... 32

2 SUPPLEMENTARY FIGURES

All measurements were made on a Bruker AVIII HD spectrometer equipped with a 9.4T Ascend 400 magnet and a 5 mm BBO Prodigy cryoprobe. No modification was required to the spectrometer beyond removing the sample rack to allow the syringe drive to be mounted to the SampleXpress platform, and connection of the TTL trigger cable to the T-controller on the AQS board of the console (Note: The position of the TTL input varies with console design. Some consoles require a separate TTL input board).

2.1 GOLD PLATED CAPILLARY TUBE

The prototype design for SF-NMR version 2 used concentric capillaries to deliver reagents to the bottom of the NMR tube, where the high flow rate resulted in turbulence and mixing of the reagents. This design requires the capillaries containing un-mixed reagents to pass through the active volume of the NMR tube, meaning that both mixed and un-mixed reagents are observed in the spectrum (Figure S1).

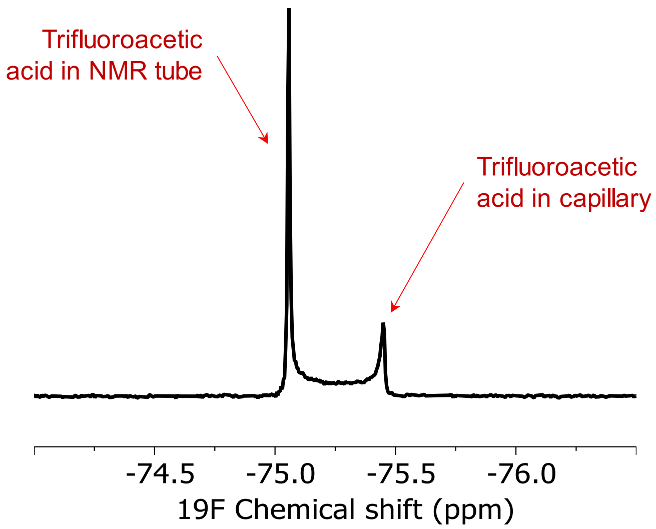

Figure S1: NMR spectrum of a sample containing trifluoracetic acid (TFA), measured using SF-NMR version 2 prototype without gold plating on the capillary tube. Separate NMR signals are observed for TFA located inside the capillary and in the outer NMR tube.

To prevent the un-mixed reagents from being observed in the NMR spectrum, the outer capillary was coated with a layer of gold plating, shielding the contents of the capillary from the radiofrequency (RF) pulses. This method was successful at shielding the contents of the capillary, however the conductivity of the gold resulted in poor matching of the RF coil and a substantial loss of signal (Figures S2 and S3).

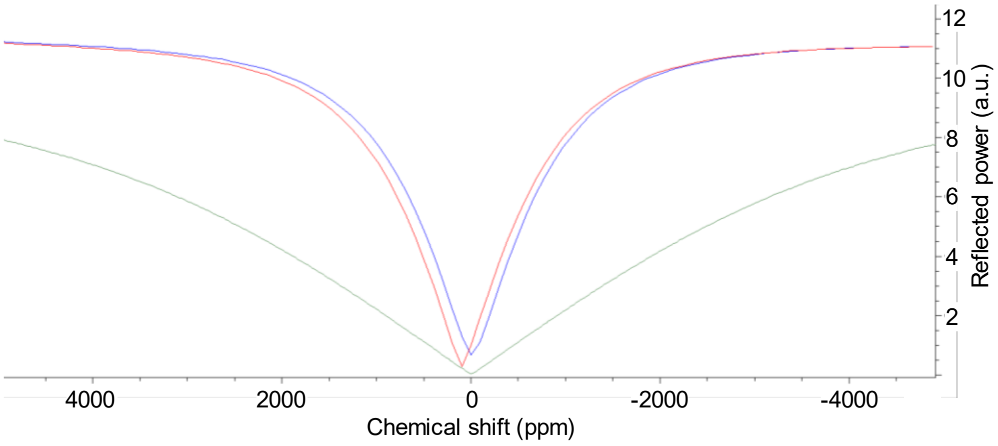

Figure S2: Frequency rejection profile recorded during probe tuning and matching of a sample of 1% $CHCl_3$ in MeOD using the SF-NMR version 2 prototype with: a) no capillary tube (blue line), b) capillary tube without gold plating (red line), c) gold plated capillary (green line).

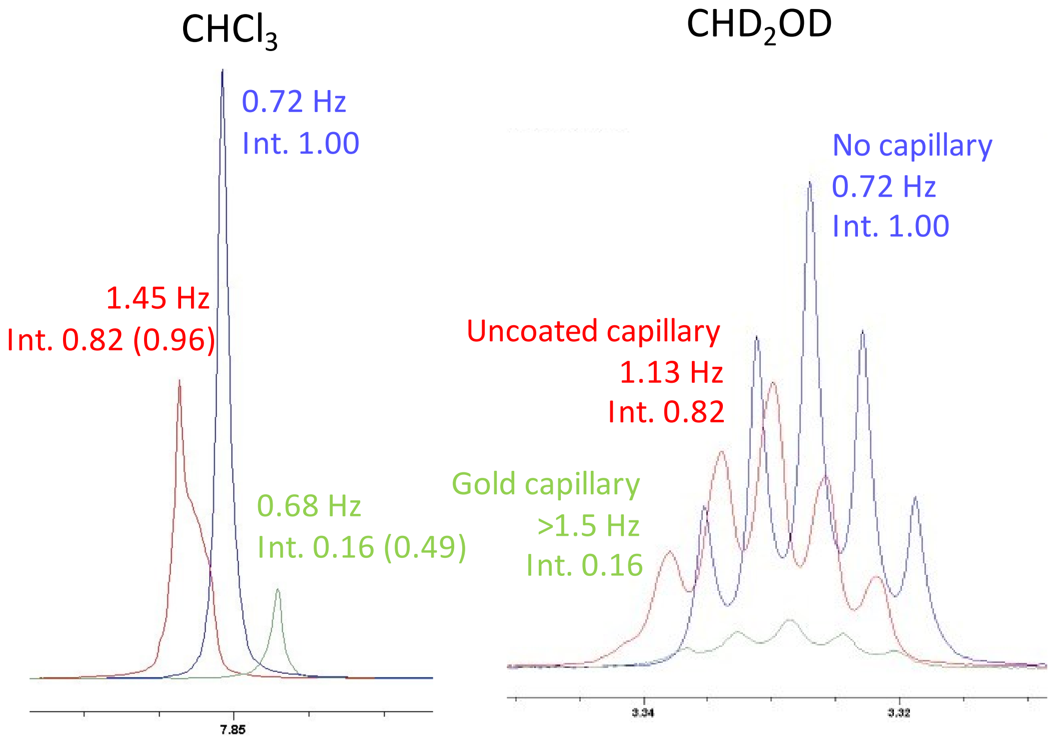

Figure S3: NMR spectra of $CHCl_3$ and CHD_2OD signals in a sample of 1% $CHCl_3$ in MeOD using the SF-NMR version 2 prototype with: a) no capillary tube (blue line), b) capillary tube without gold plating (red line), c) gold plated capillary (green line).

Additionally, the gold plating was found to be unstable to certain chemicals, with prolonged exposure to trifluoroacetic acid resulting in delamination of the gold (Figure S4).

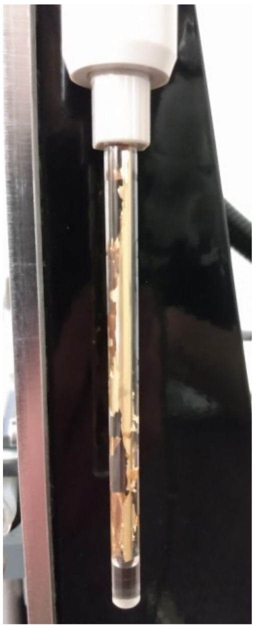

Figure S4: Photographs of the gold-plated capillary tube after exposure to trifluoroacetic acid, resulting in delamination of the gold plating.

2.2 MIXING EFFICIENCY

Mixing efficiency was assessed visually by high-speed video footage recorded during the mixing of an ethanol solution of bromothymol blue (inner capillary) and an ethanol solution of hydrochloric acid (outer capillary) at a combined flowrate of 8 mL/s (Figure S5). The syringe pump was started at frame 0. During the first 38 frames, the reagents flowed into the NMR tube displacing the previous contents (ethanol, colourless). By Frame 88 approximately 600 µL of reagents were delivered and the mixing reached a steady state. After actuating the flow diversion valves (frame 188), the flow within the NMR tube is rapidly arrested. Pressure waves result in a brief period of unstable flow (frames 188 to 208), with reagents first drawn back up into the capillary tubes, followed by small oscillations (<3 mm) which are damped out by the check valves, trapping a stationary sample within the NMR tube (frame 228).

Pressure measurements were made by addition of a tee into the reagent and/or waste lines immediately above the premagnetisation block. A fast acting (< 1 millisecond full-scale rise time) pressure transducer (Honeywell 24PCGFM6G, 250 psi, 10 V) was connected to the tee, and changes in voltage across the transducer measured using a PicoScope 3403D MSO digital oscilloscope (Pico Technologies). Pressure values were calculated from the measured voltage using the calibration curve supplied by the manufacturer, with accuracy verified by comparison to an IDEX QuickStart I2C PS200F EVAL pressure sensor (Note: it was not possible to use the IDEX sensor for pressure measurements in flow due to the 67 millisecond rise time of this sensor). The oscilloscope measurement was triggered using the TTL output from the syringe drive, with time point zero corresponding to the trigger signal used to close the valve and start the NMR acquisition. The position of the syringe drive was measured using a linear potentiometer attached to the drive plate, with voltage output measured using the same oscilloscope (up to four pressure/position sensors were connected simultaneously). A linear calibration was performed, relating voltage across the potentiometer to measured position (and therefore delivered volume) of the syringe drive.

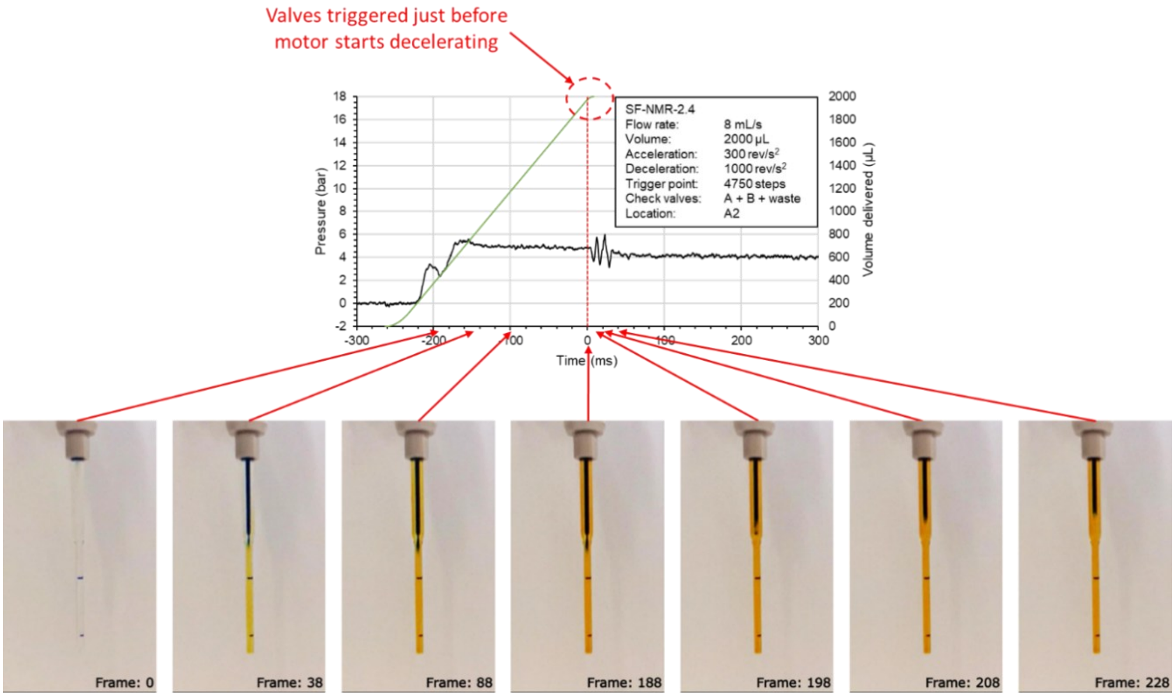

Figure S5: Still images from high-speed video footage (960 frames per second) overlaid with the pressure within the NMR tube. Recorded during the mixing of and ethanol solution of bromothymol blue (inner capillary) and an ethanolic solution of hydrochloric acid (outer capillary) at a combined flowrate of 8 mL/s (Note: Higher flow rates of 10 mL/s were used in the final design. This has little to no impact on the mixing efficiency or stopping time as can be observed in Figures S7, S8 and the supporting video).

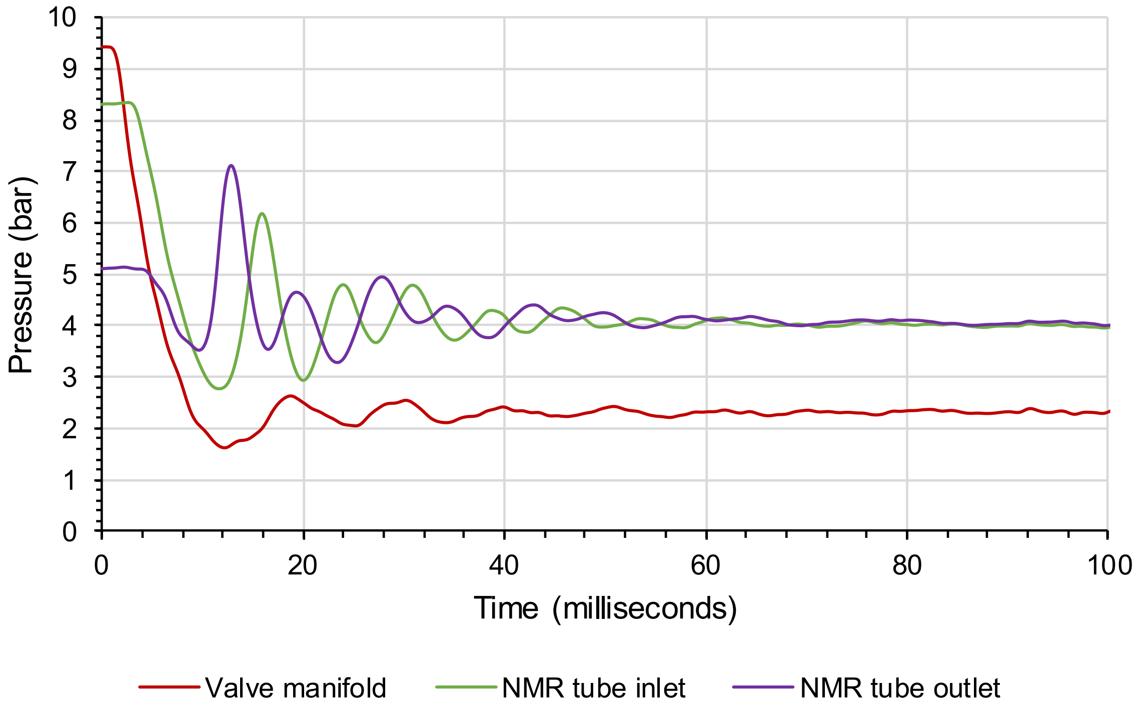

Figure S6: Pressure oscillations in SF-NMR-V2 immediately following actuation of the flow diversion valves (10 mL/s). Pressure waves oscillate between the check valves on the inlet and outlet of the NMR tube, resulting in an echo-like signal where the pressure at the inlet and outlet of the NMR tube are out of phase.

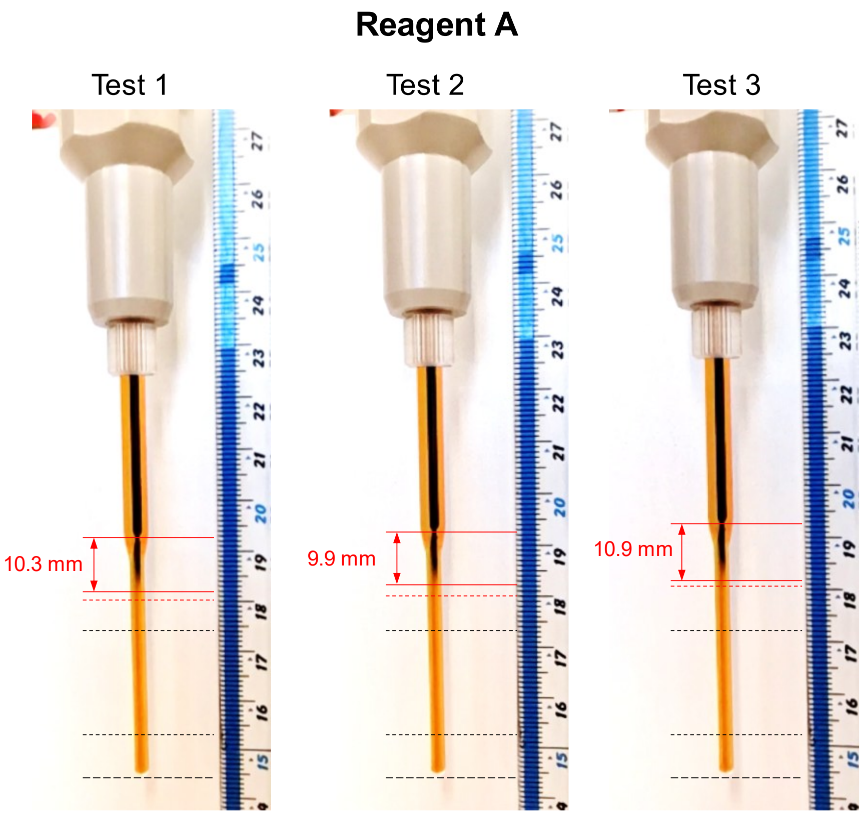

Figure S7: Still images from high-speed video footage (960 frames per second) recorded during three repeats of mixing of an ethanol solution of bromothymol blue (inner capillary) and an ethanolic solution of hydrochloric acid (outer capillary) at a combined flowrate of 10 mL/s.

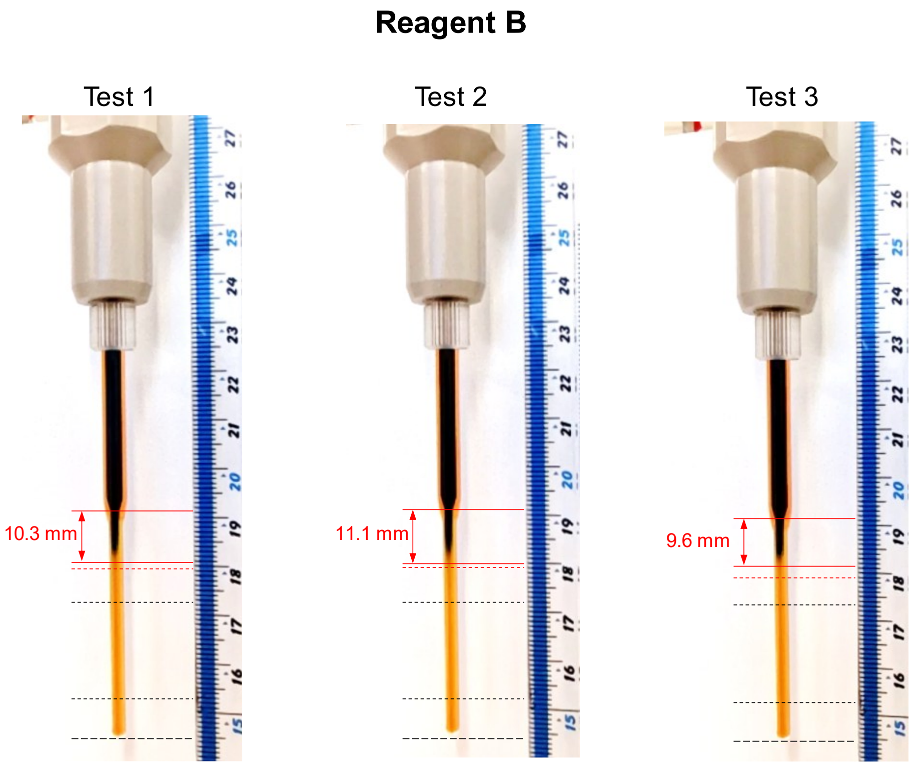

Figure S8: Still images from high-speed video footage (960 frames per second) recorded during three repeats of mixing of an ethanol solution of bromothymol blue (outer capillary) and an ethanolic solution of hydrochloric acid (inner capillary) at a combined flowrate of 10 mL/s.

2.3 EFFECT OF MOTOR DRIVE ON SHIMMING

The addition of the stepper motor drive to the top of the shielded magnet has a significant impact on the quality of the NMR line shape (Figure S9). When the stepper motor is in motion, small distortions can be seen in the baseline, however no effect was observed when switching the solenoid valves on/off during the NMR measurement. All effects are easily corrected by the spectrometer shim system, resulting in a line shape that is equal to that without the motor drive in place.

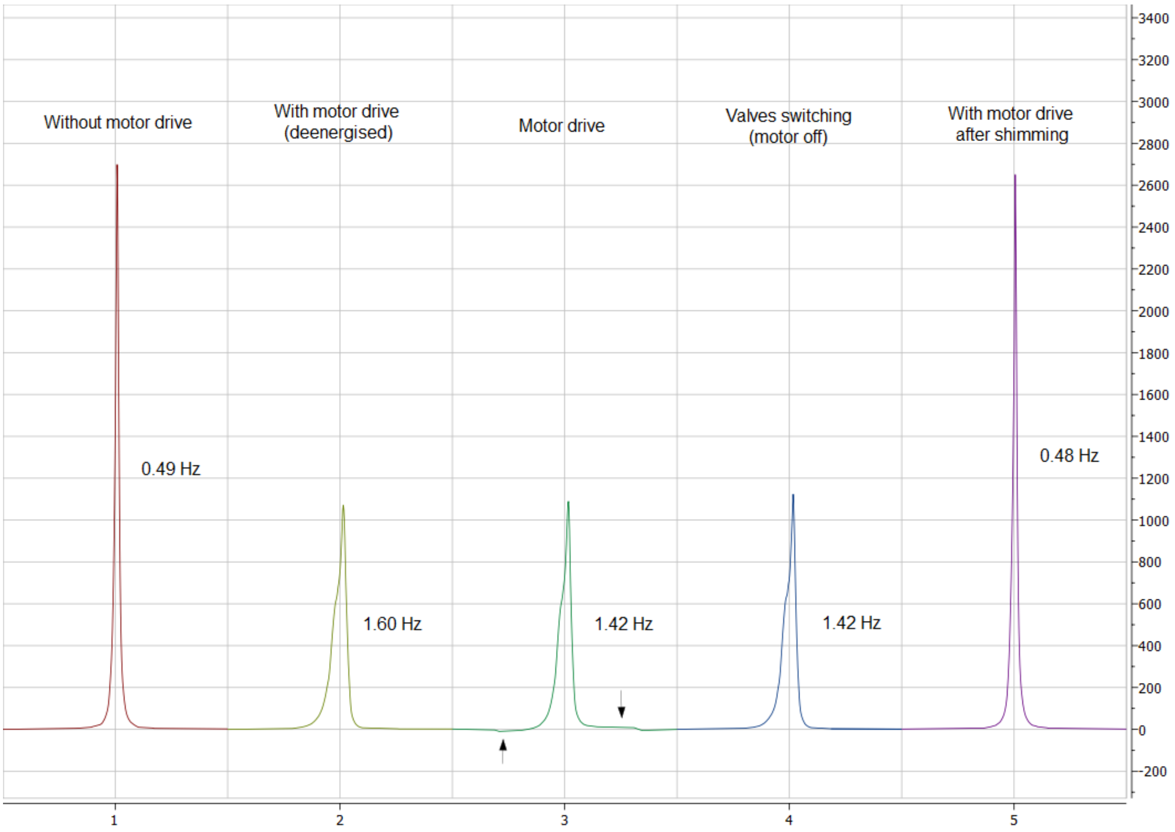

Figure S9: Spectra of the $CHCl_3$ peak in a sample containing 1% $CHCl_3$ in acetone-d_6 (standard 5 mm NMR tube) acquired under various configurations of the SF-NMR-V2 instrument: 1) without the motor drive in place, 2) with the motor drive in place on top of the magnet, but with the stepper motor powered off, 3) with the motor drive in place and motor in motion, 4) with the motor stationary and the flow diversion valves actuated, 5) with the motor drive in place, after re-shimming the magnet. Half-height line widths are indicated for each peak in units of Hertz.

2.4 Simulation of the effect of the instrument dead-time on reaction kinetics

Reaction kinetics were simulated using Microsoft Excel for the integrated rate equations of zero, first and second order reactions using the SF-NMR insert, with a 1M starting concentration, zero-order rate constant = 10 M.s^{-1}, first-order rate constant = 50 s^{-1}, second-order rate constant = 500 M^{-1}.s^{-1}. In each case, the reaction was simulated for 16 regions with initial sample ages of 1.7, 2.1, 2.4, 2.9, 3.3, 3.7, 4.1, 4.5, 7.6, 8.3, 9.0, 9.7, 10.4, 11.1, 11.8 and 12.5 milliseconds, corresponding to approximately 2.6 mm slices through the NMR observation volume (zones C/F in Figure 11). Reaction kinetics were simulated for each region in 0.1 millisecond steps for a total duration of 100 milliseconds. A weighted average of the concentrations at each time point was taken, based on the sample volume that each region represents (28 µL for zone C and 48 µL for zone F). The resulting data was fitted using a kinetic model to determine the apparent rate constant that would be observed in each case.

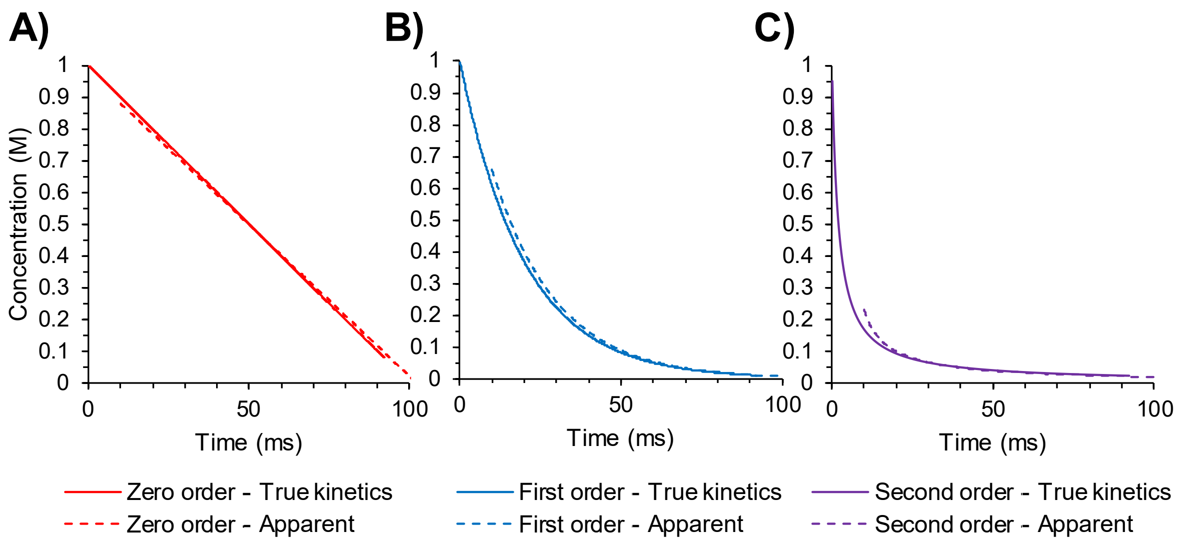

Figure S10: Concentration vs time plots for simulated reaction kinetics using the SF-NMR insert. A) Zero-order reaction, rate constant = 10 M.s^{-1}, b) First-order reaction, rate constant = 50 s^{-1}, c) Second-order reaction, rate constant = 500 M^{-1}.s^{-1}. 1M starting concentration. Kinetics simulated for the volume-weighted combination of regions C (28 µL) and F (48 µL), with initial ages spanning 1.7 to 4.5 milliseconds and 7.6 to 12.5 milliseconds respectively, and each subdivided into 8 temporal sections.

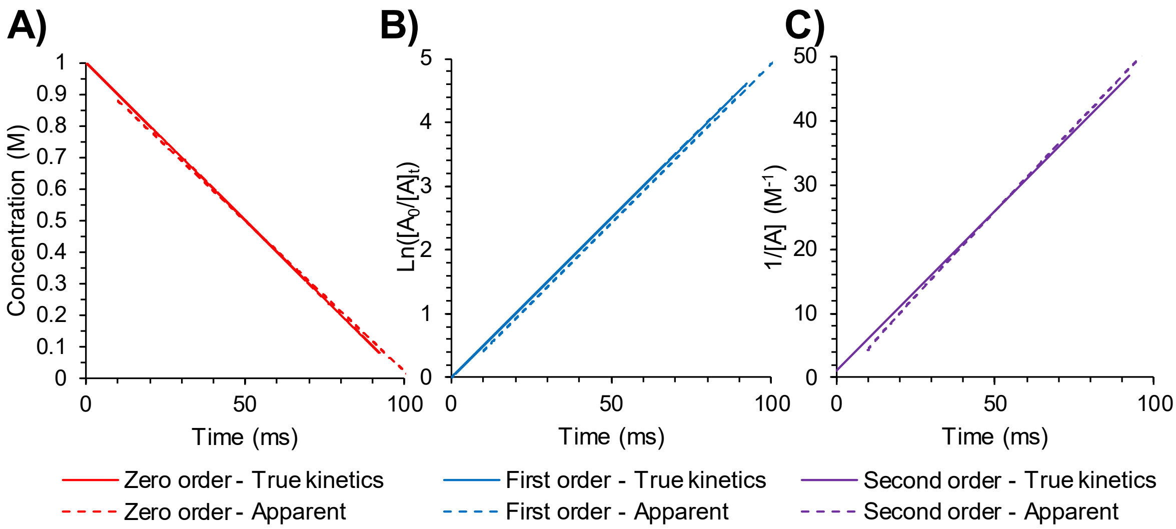

Figure S11: Linearised concentration vs time plots for simulated reaction kinetics using the SF-NMR insert. A) Zero-order reaction, rate constant = 10 M.s^{-1}, b) First-order reaction, rate constant = 50 s^{-1}, c) Second-order reaction, rate constant = 500 M^{-1}.s^{-1}. 1M starting concentration. Kinetics simulated for the volume-weighted combination of regions C (28 μL) and F (48 μL), with initial ages spanning 1.7 to 4.5 milliseconds and 7.6 to 12.5 milliseconds respectively, and each subdivided into 8 temporal sections.

2.5 DEUTEROLYSIS OF METHYL FORMATE, MONITORED BY ^{1}H NMR.

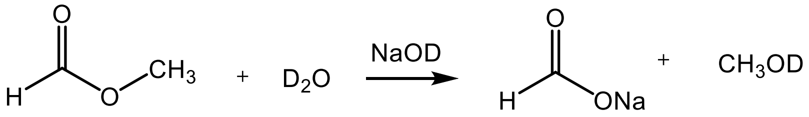

Scheme 1: The base-mediated deuterolysis of methyl formate.

Stock solutions of a) 0.200 M dimethyl sulfoxide (internal concentration reference) and 0.502 M methyl formate in deuterated water, and b) 2.06 M sodium deuteroxide in deuterated water were prepared. Both stock solutions were degassed by sonication for 10 minutes. The SF-NMR-V2 instrument was primed with degassed water followed by deuterated water to ensure that no gas bubbles remained within the tubing. The stock solutions were connected to the syringe drive and the syringes and reagent lines primed with 6 mL of each reagent. The solvent flush syringe was connected to a bottle containing degassed D_2O. Silicone oil was circulated around the SF-NMR-V2 insert using a recirculating heater/chiller to maintain a constant temperature of 300 K.

The NMR umbilical was inserted into the NMR probe and locking, tuning and shimming performed using automated routines. The probe temperature was set to 300 K. To acquire a spectrum using the SF-NMR-V2 instrument, a ^{1}H NMR experiment was prepared using a 90° pulse programme containing a trigger input followed by a variable delay before the acquisition (see Section 4 for further details). The NMR experiment was started, with the spectrometer waiting for the trigger input before starting the acquisition. The SF-NMR-V2 syringe drive was commanded to deliver a reaction shot of 600 µL at a combined flow rate of 10 mL/s, with the syringe drive sending simultaneous trigger signals to the spectrometer and to the solenoid valves after 533 µL had been delivered.

The experiment was repeated with the variable delay between the trigger input and the start of the NMR acquisition incremented in 10 millisecond intervals. Each experiment was performed on a fresh injection of reagents. The NMR tube was flushed with degassed D_2O prior to each experiment, and a delay of at least 30 seconds was allowed between each experiment to ensure full pre-magnetisation of the reagents. The resulting spectra were integrated using TopSpin 4.3 to calculate concentrations of each species. Reaction kinetics were modelled numerically to calculate the second-order rate constant.

2.6 PROTODEBORONATION OF PENTAFLUOROPHENYL BORONATE, MONITORED BY ^{19}F NMR.

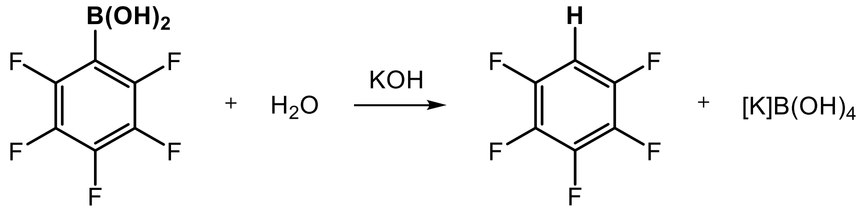

Scheme 2: Base-mediated protodeboronation of pentafluorophenyl boronic acid.

Stock solutions of a) 0.100 M pentafluorophenylboronic acid and 0.0522 M trifluoracetic acid (internal concentration reference) in 1:1 dioxane:H_2O mixture, and b) 0.311 M potassium hydroxide in 1:1 dioxane:H_2O mixture were prepared. Both stock solutions were degassed by sonication for 10 minutes. The SF-NMR-V2 instrument was primed with 1:1 dioxane:H_2O to ensure that no gas bubbles remained within the tubing. The stock solutions were connected to the syringe drive and the syringes and reagent lines primed with 6 mL of each reagent. The solvent flush syringe was connected to a bottle containing 1:1 dioxane:H_2O mixture. Silicone oil was circulated around the SF-NMR-V2 insert using a recirculating heater/chiller to maintain a constant temperature.

The NMR umbilical was inserted into the NMR probe, the probe temperature was set, and tuning and ^{1}H shimming performed using automated routines. Locking was not performed as the solvent did not contain deuterium. To acquire a spectrum using the SF-NMR-V2 instrument, a ^{19}F NMR experiment was prepared using a 90° pulse programme containing a trigger input followed by a variable delay before the acquisition (see Section 4 for further details). The NMR experiment was started, with the spectrometer waiting for the trigger input before starting the acquisition. The SF-NMR-V2 syringe drive was commanded to deliver a reaction shot of 600 µL at a combined flow rate of 10 mL/s, with the syringe drive sending simultaneous trigger signals to the spectrometer and to the solenoid valves after 533 µL had been delivered.

The experiment was repeated with the variable delay between the trigger input and the start of the NMR acquisition incremented. Each experiment was performed on a fresh injection of reagents. The NMR tube was flushed with 1:1 dioxane:H_2O mixture prior to each experiment, and a delay of at least 30 seconds was allowed between each experiment to ensure full pre-magnetisation of the reagents. The resulting spectra were integrated using TopSpin 4.3 to calculate concentrations of each species. Reaction kinetics were modelled numerically to calculate the rate constants.

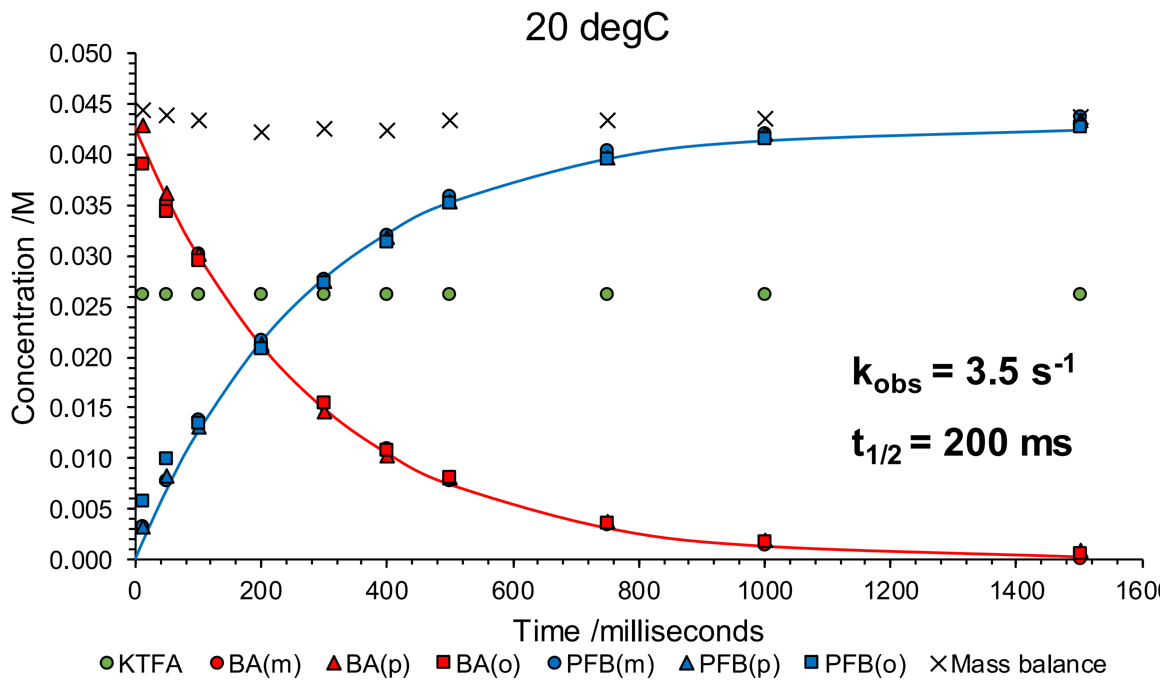

Figure S12: Kinetics of the protodeboronation of potassium pentafluorophenyltrihydroxy boronate at 20°C, determined by ^{19}F NMR spectroscopy using the SF-NMR-V2 instrument. Conditions: 0.05 M boronic acid, 0.16 M potassium hydroxide in 1:1 dioxane:water mixture, 600 µL injection volume, 10 mL/s. The *x*-axis is a pseudo time-scale with each data point obtained from a separate injection of reagents with time increments of 10, 50, 100, 200, 300, 400, 500, 750 1000 and 1500 milliseconds prior to application of the 90 ° pulse / FID acquisition.

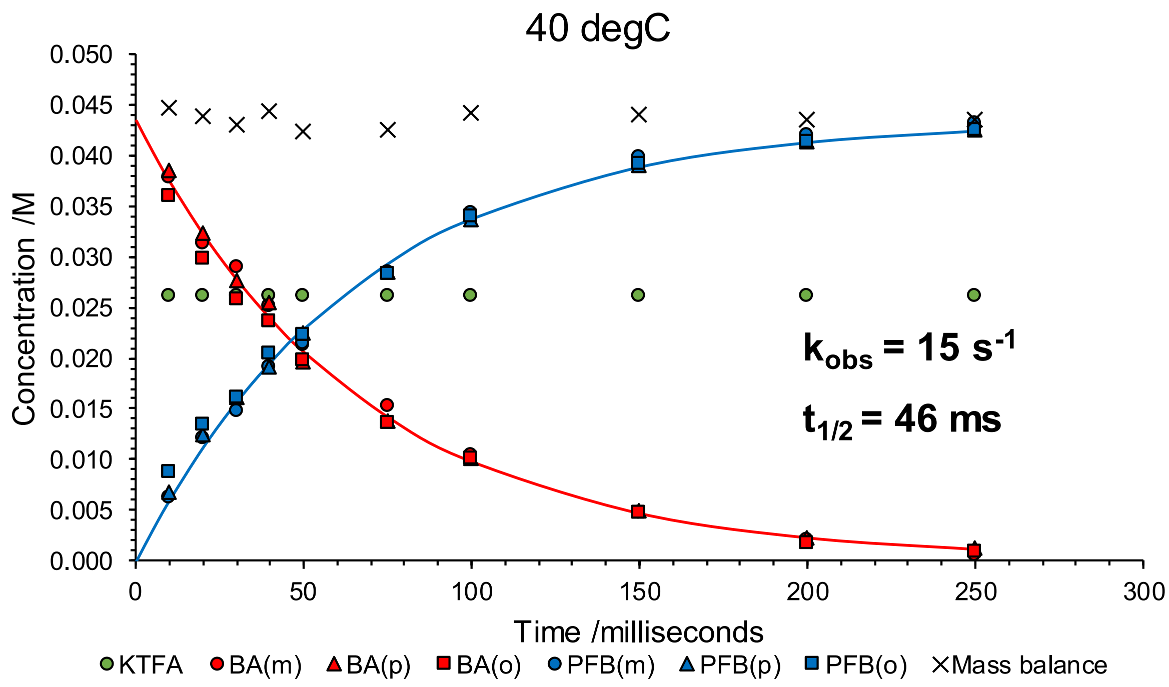

Figure S13: Kinetics of the protodeboronation of potassium pentafluorophenyltrihydroxy boronate at 40°C, determined by ^{19}F NMR spectroscopy using the SF-NMR-V2 instrument. Conditions: 0.05 M boronic acid, 0.16 M potassium hydroxide in 1:1 dioxane:water mixture, 600 µL injection volume, 10 mL/s. The *x*-axis is a pseudo time-scale with each data point obtained from a separate injection of reagents with time increments of 10, 20, 30, 40, 50, 75, 100, 150, 200 and 250 milliseconds prior to application of the 90 ° pulse / FID acquisition.

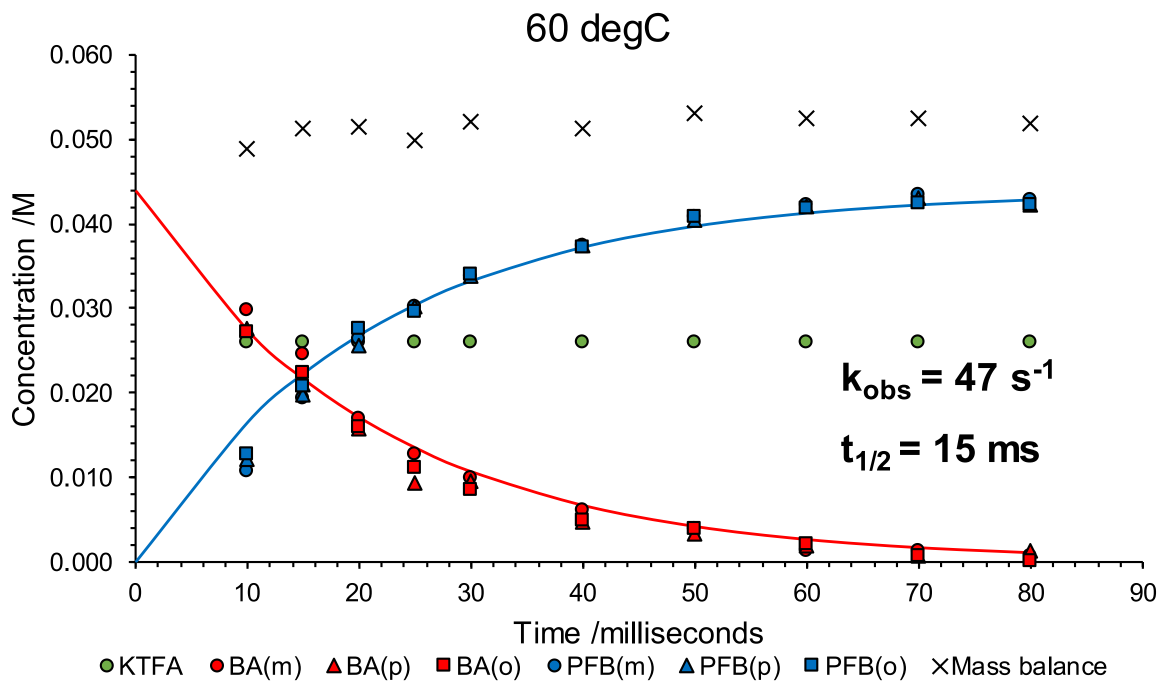

Figure S14: Kinetics of the protodeboronation of potassium pentafluorophenyltrihydroxy boronate at 60°C, determined by ^{19}F NMR spectroscopy using the SF-NMR-V2 instrument. Conditions: 0.05 M boronic acid, 0.16 M potassium hydroxide in 1:1 dioxane:water mixture, 600 µL injection volume, 10 mL/s. The *x*-axis is a pseudo time-scale with each data point obtained from a separate injection of reagents with time increments of 10, 15, 20, 25, 30, 40, 50, 60, 70, and 80 milliseconds prior to application of the 90 ° pulse / FID acquisition.

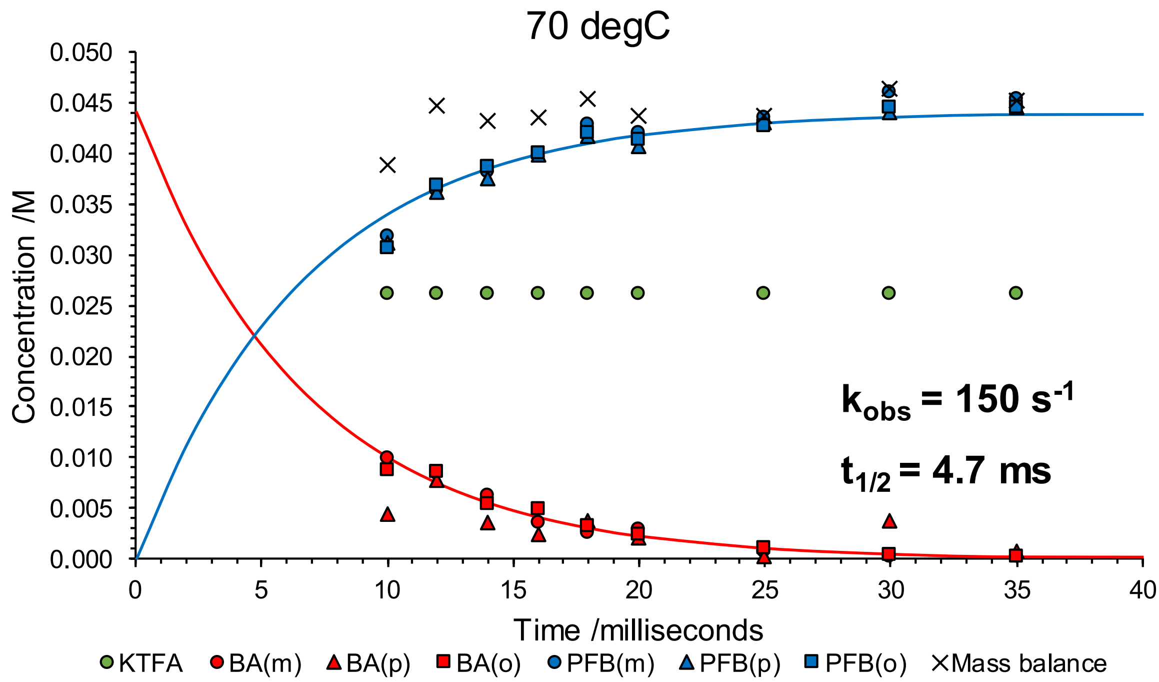

Figure S15: Kinetics of the protodeboronation of potassium pentafluorophenyltrihydroxy boronate at 70°C, determined by ^{19}F NMR spectroscopy using the SF-NMR-V2 instrument. Conditions: 0.05 M boronic acid, 0.16 M potassium hydroxide in 1:1 dioxane:water mixture, 600 µL injection volume, 10 mL/s. The *x*-axis is a pseudo time-scale with each data point obtained from a separate injection of reagents with time increments of 10, 12, 14, 16, 18, 20, 25, 30 and 35 milliseconds prior to application of the 90 ° pulse / FID acquisition.

2.7 FLOW PATH WITHIN NMR INSERT

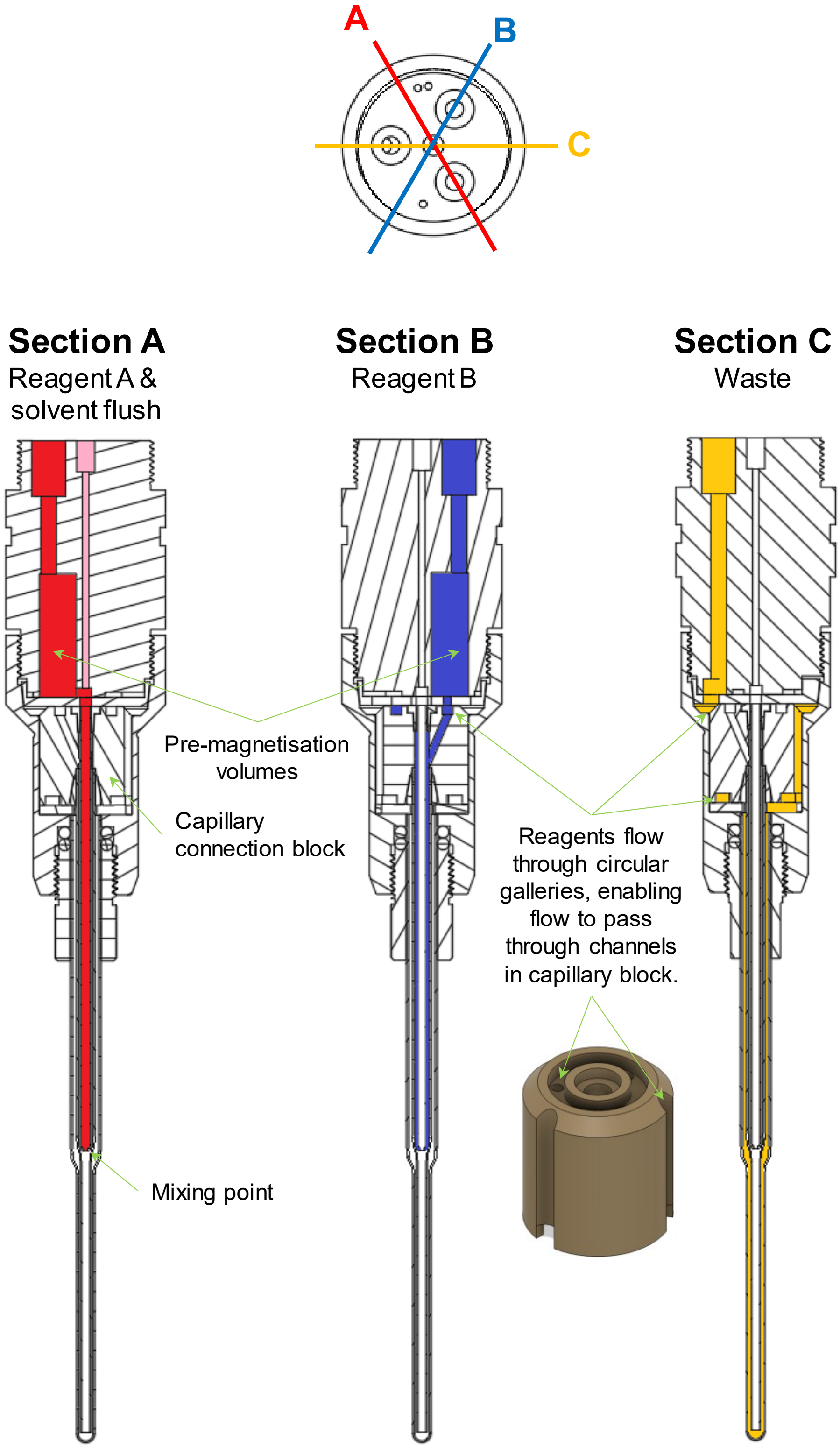

Figure S16: Cross-section of the NMR insert at angles of 0°, 60° and 120°, showing a) the flow path of reagent A (red) and the solvent flush (pale red), b) the flow path of reagent B, c) the flow path of the waste exiting the NMR tube. Reagent B and waste flow through circular galleries in the capillary block (see inset image) which enable the flow to distribute evenly around the annular space between the capillaries.

3 PHOTOGRAPHS OF SF-NMR-V2 INSTRUMENT

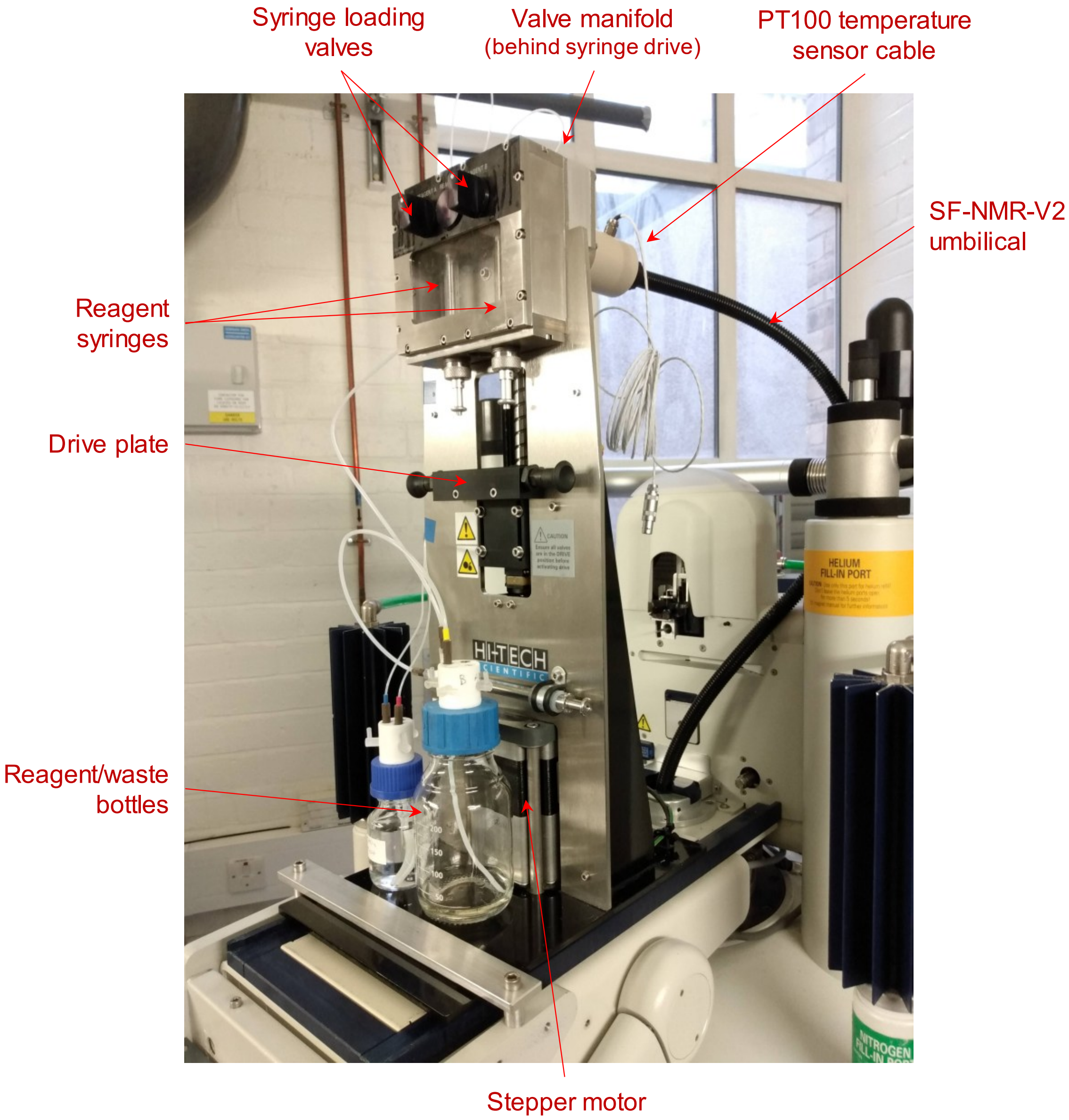

Figure S17: Photograph of SF-NMR-V2 instrument in position on top of NMR magnet, with key components labelled.

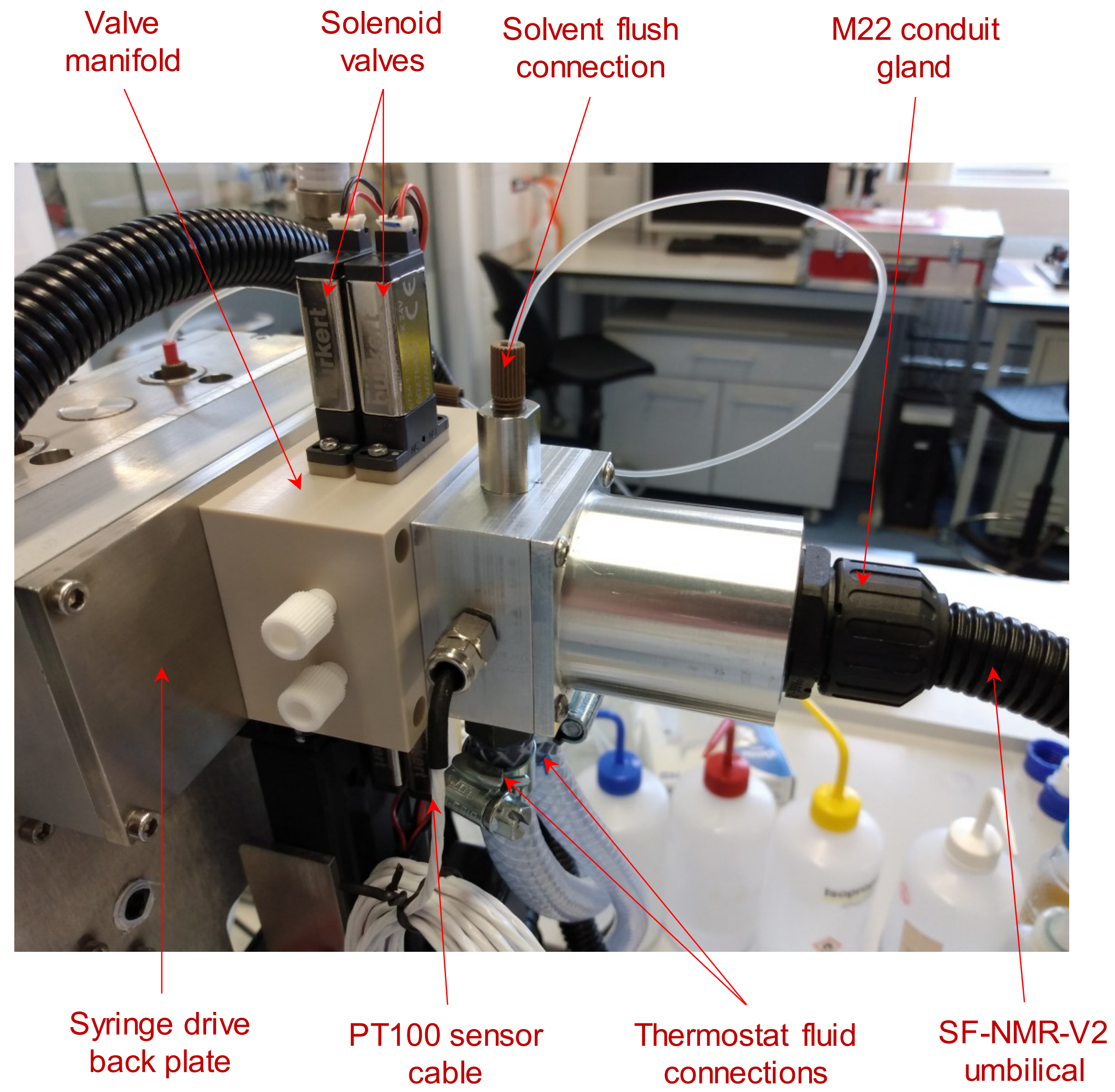

Figure S18: Photograph of SF-NMR-V2 valve manifold attached to syringe drive, with key components labelled.

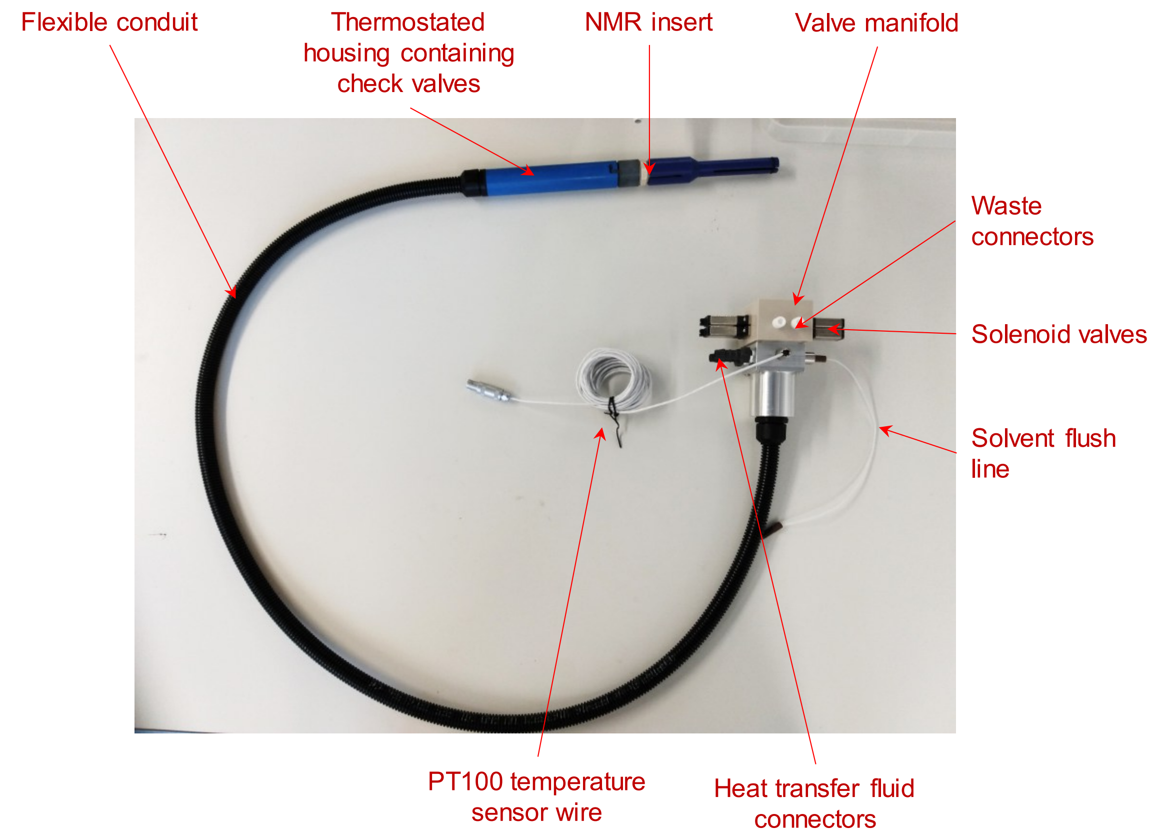

Figure S19: Photograph of SF-NMR-V2 umbilical detached from syringe drive, with key components labelled.

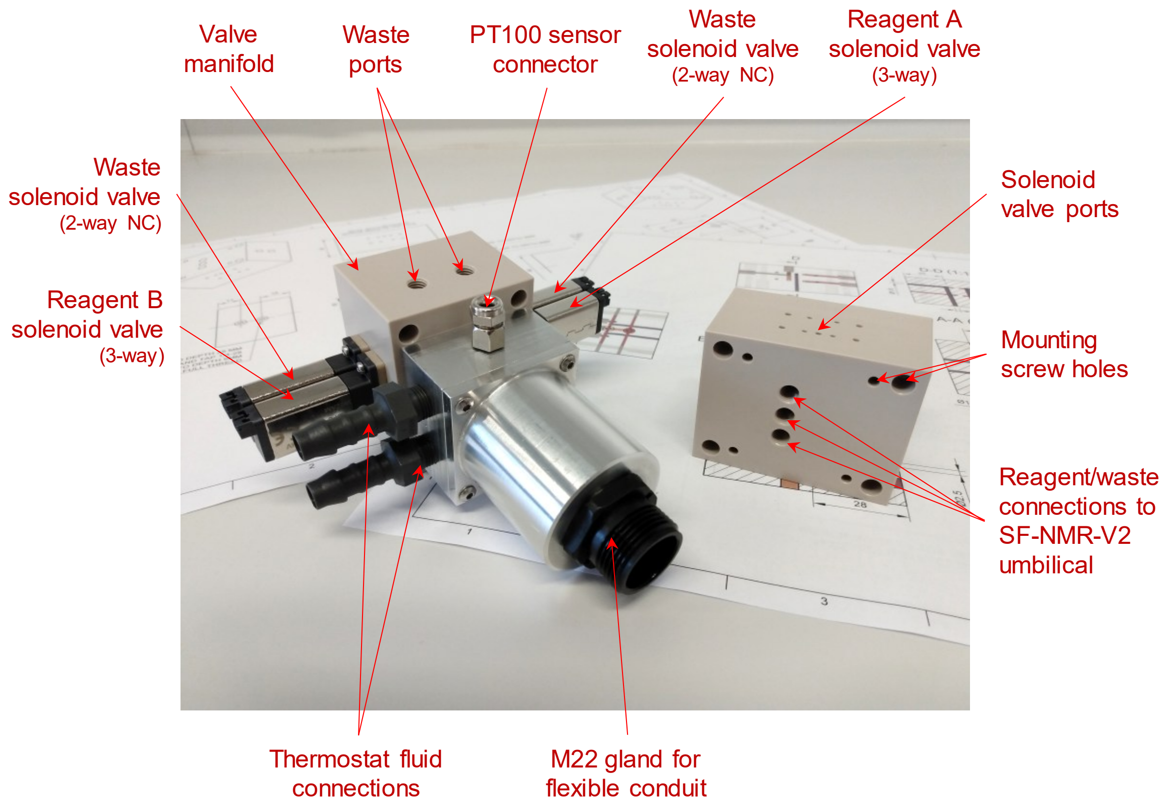

Figure S20: Photograph of SF-NMR-V2 valve manifold detached from umbilical, with key components labelled.

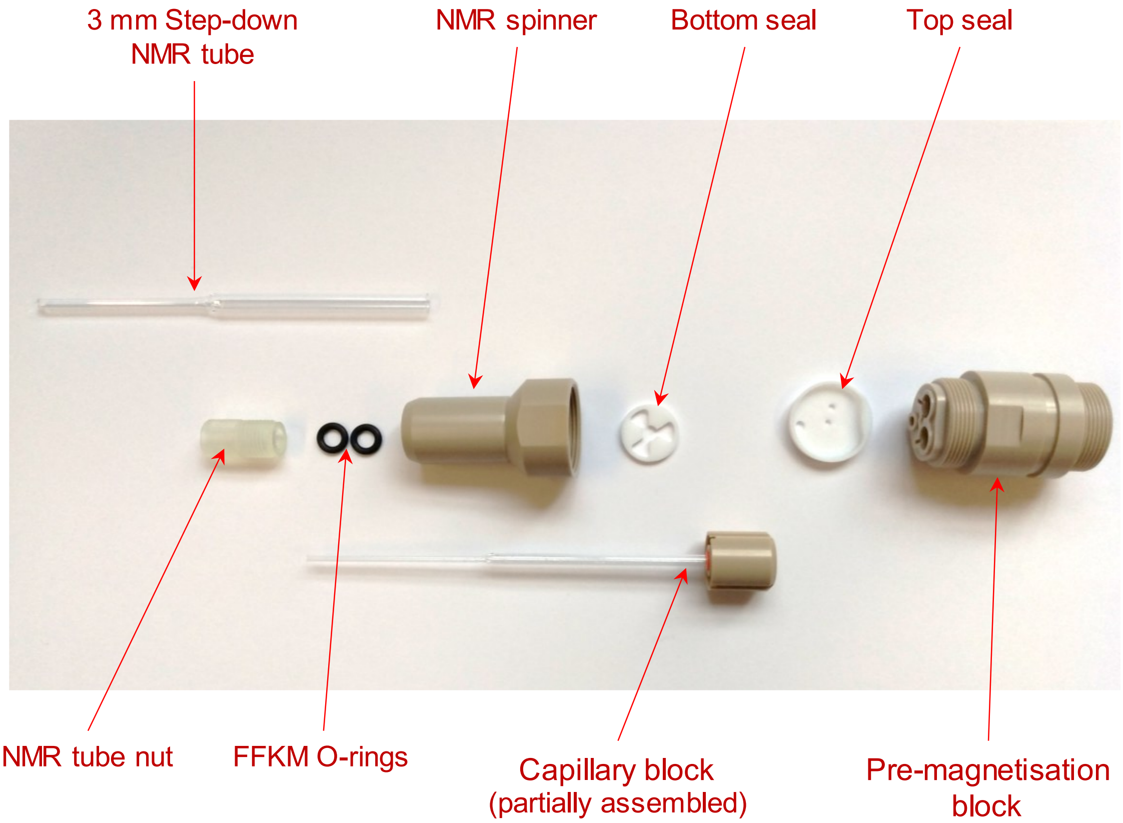

Figure S21: Photograph of partially dismantled SF-NMR-V2 NMR tube insert, with key components labelled.

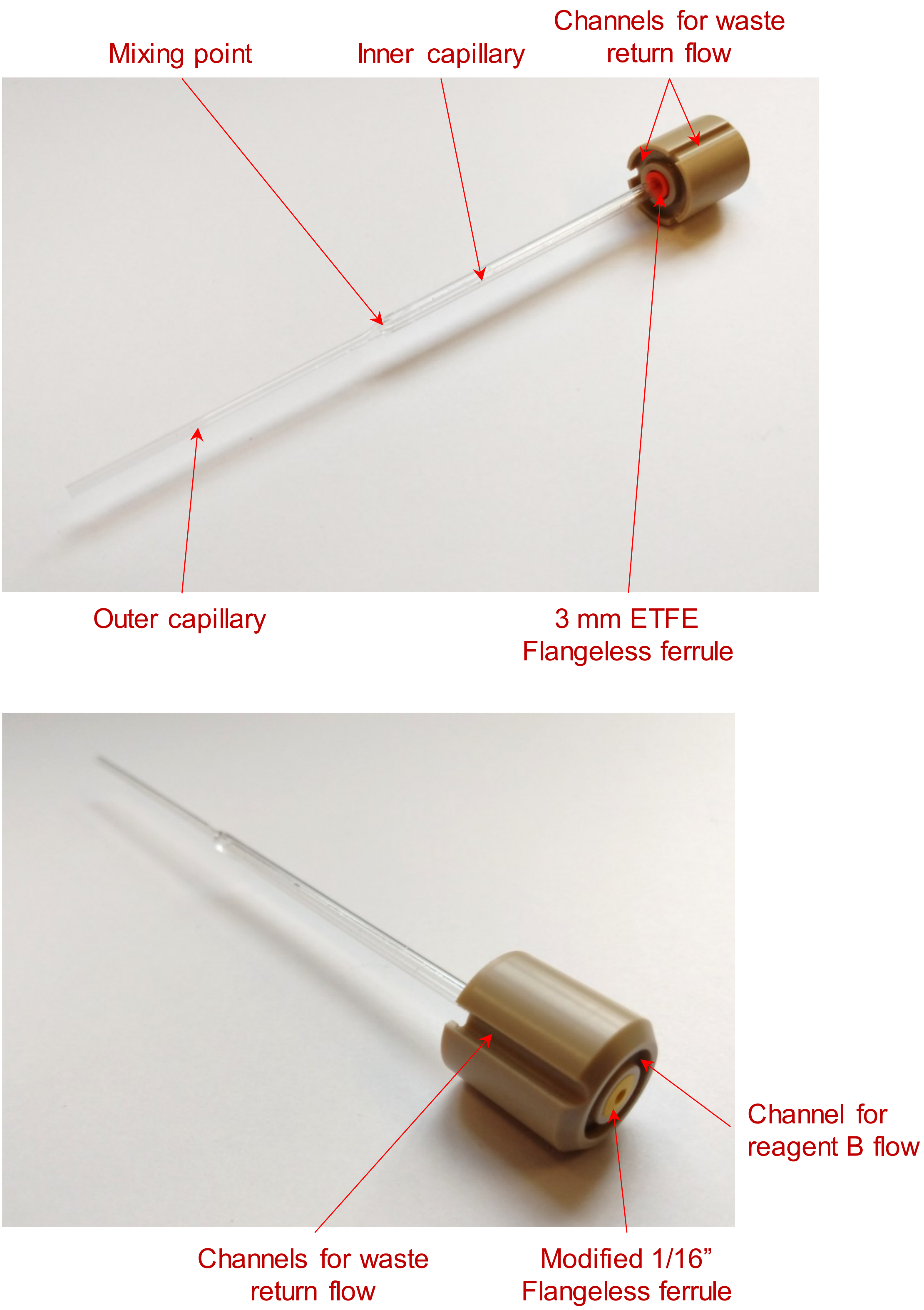

Figure S22: Two photographs of partially assembled SF-NMR-V2 capillary block, showing attachment of inner and outer capillaries, flow paths and with key components labelled.

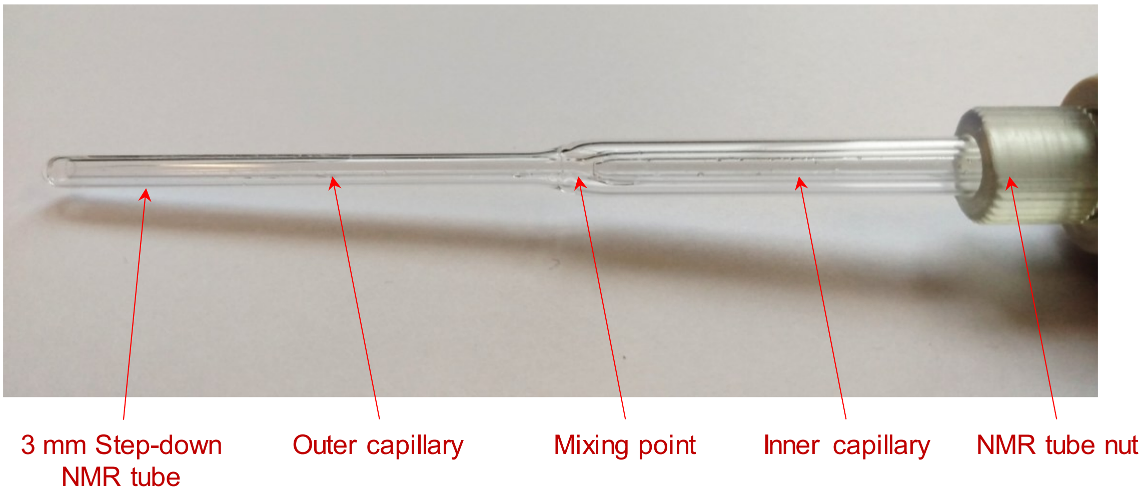

Figure S23: Photograph of SF-NMR-V2 NMR tube, with key components labelled.

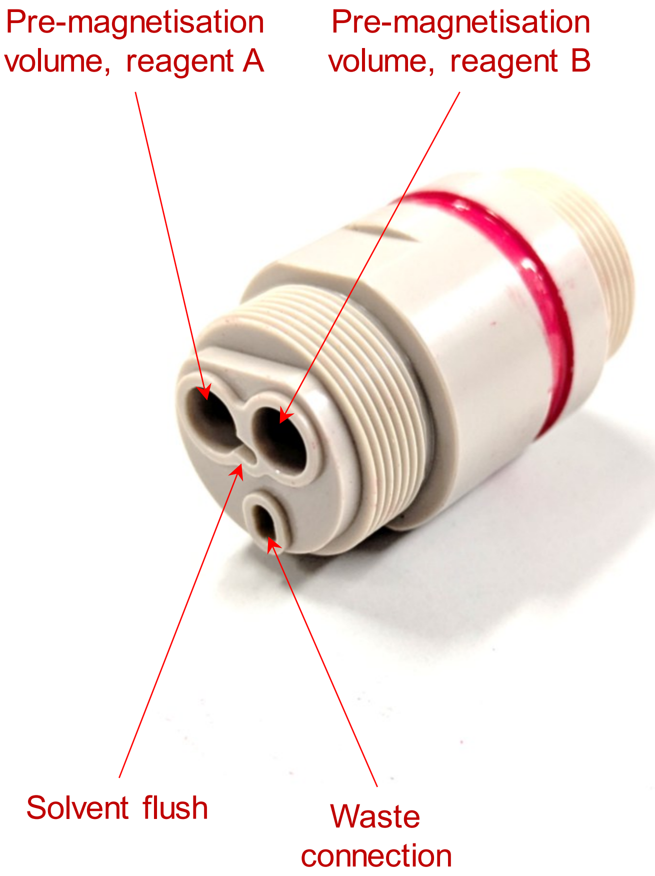

Figure S24: Photograph of SF-NMR-V2 pre-magnetisation block, showing pre-magnetisation volumes.

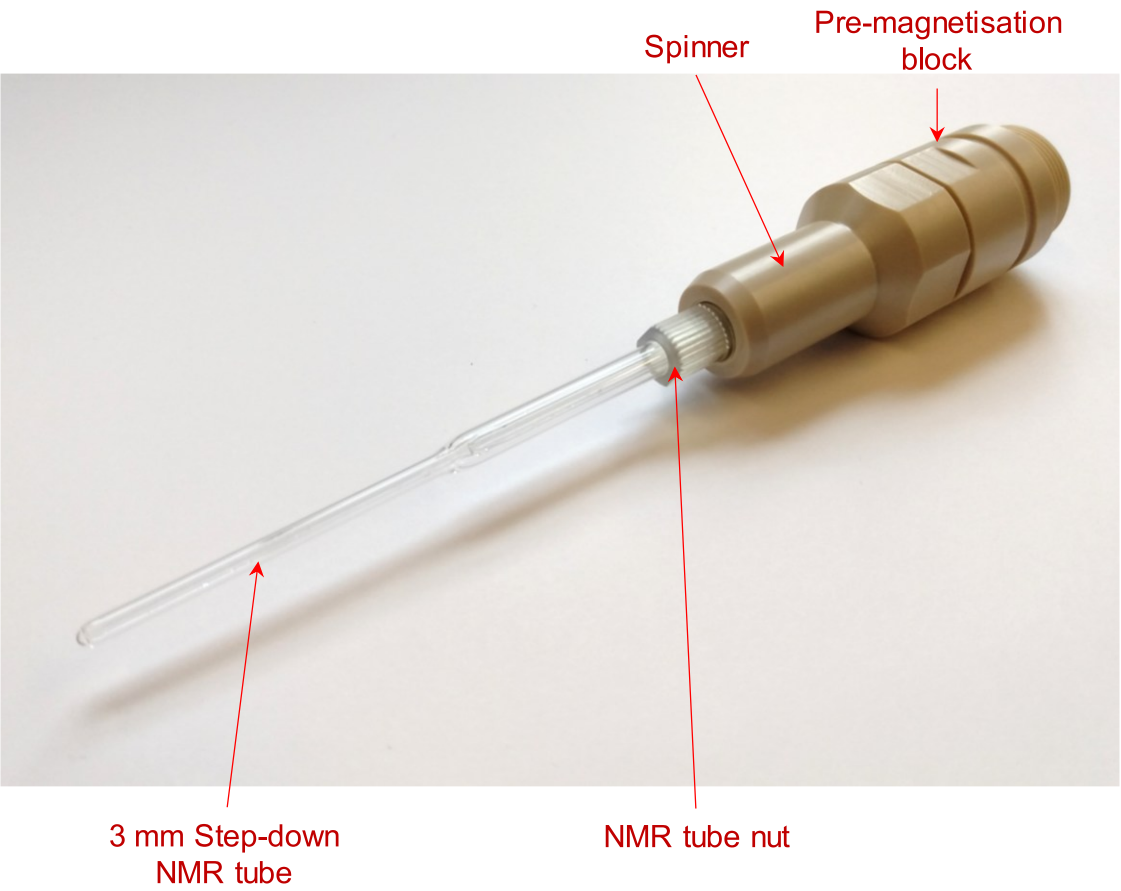

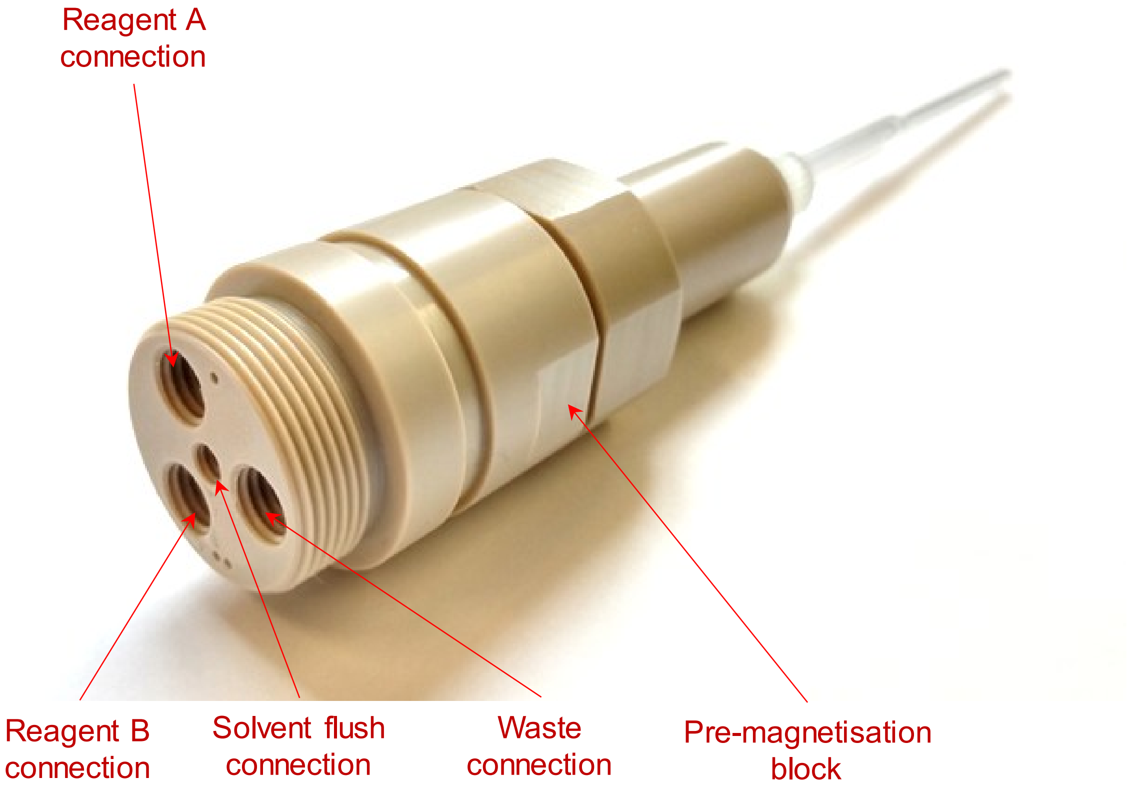

Figure S25: Two photographs of partially assembled SF-NMR-V2 NMR insert, showing reagent connections and with key components labelled.

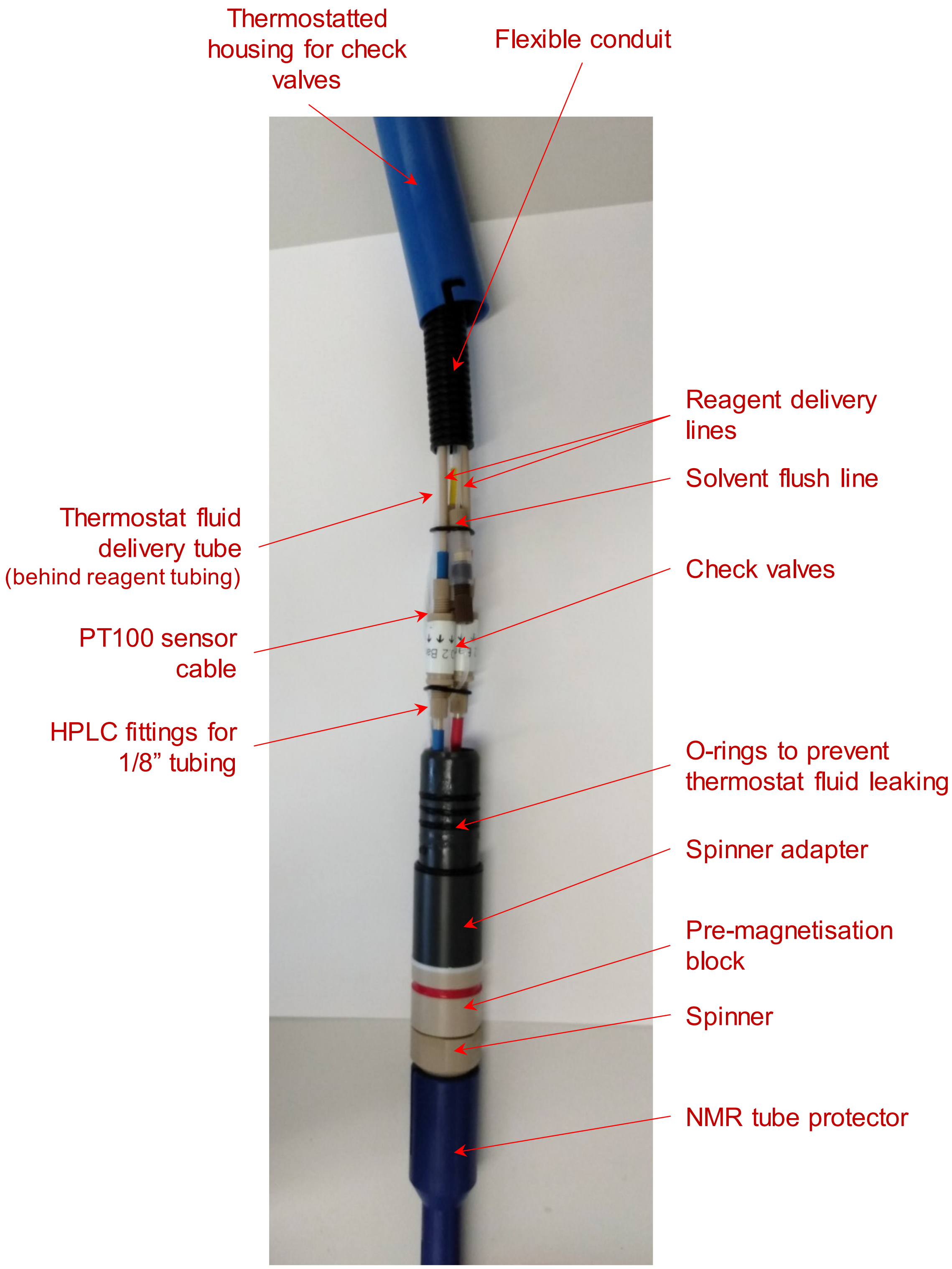

Figure S26: Photograph of SF-NMR-V2 umbilical, with housing removed to show location of check valves, and with key components labelled.

4 NMR PULSE SEQUENCE

This pulse programme has been tested on a Bruker AVANCE III HD spectrometer with an integrated IPSO/AQS module. The location of trigger outputs and 'setnmr' commands may vary depending on spectrometer model – **Use of this pulse programme is at your own risk. Please check this pulse programme carefully for compatibility with your spectrometer before use and consult the instrument AQS manual for further details!**

```
;zg_trig_nodelay

;avance-version (12/01/11)
;1D sequence with trigger
;30 ms read/write delay removed
;
;$CLASS=HighRes
;$DIM=1D
;$TYPE=
;$SUBTYPE=
;$COMMENT=
;$RECOMMEND=y
;$OWNER=Andrew Hall

#include <Avance.incl>

"acqt0=-p1*0.66/3.1416"

1 ze
  1u trigne1
2 1u
  d7
  p1 ph1
  go=2 ph31
  wr #0
exit

ph1=0 2 2 0 1 3 3 1
ph31=0 2 2 0 1 3 3 1

;pl1 : f1 channel - power level for pulse (default)
;p1 : f1 channel -  90 degree high power pulse
;d7 : sample ageing time/trigger delay
;ns: 1 * n, total number of scans: NS * TD0
```

5 DESIGN DRAWINGS AND MATERIALS

5.1.1 Design notes

The SF-NMR-V2 instrument consists of a custom NMR umbilical which is inserted into the NMR probe, and a SF-73 two-syringe stepper motor drive (TgK Scientific). The NMR umbilical attaches to the back of the SF-73 syringe drive and does not require any modification of the syringe drive connections.

The NMR umbilical consists of an NMR insert, containing pre-magnetisation volumes, NMR spinner, 3 mm step-down NMR tube, check valves and capillary tubes for delivering reagents. The NMR insert is connected to a valve manifold which is mounted on the back of the SF-73 syringe drive via a 120 cm length flexible conduit containing reagent delivery lines, solvent flush line, waste line and PT100 temperature sensor, all immersed in a heat transfer fluid. The valve manifold contains connectors to receive reagents from the syringe drive, four rapidly acting solenoid valves for flow diversion, connectors for heat transfer fluid and connectors for waste and solvent flush tubing.

5.1.1.1 Valves

Fast acting (<3 milliseconds) solenoid valves were purchased from Burkert. Three-way diversion valves (part number 6724-T01) were used for reagent flow diversion, and two-way (NC) valves (part number 6724-A01) were used to close the waste line. To reduce pressure loss due to the high flow-rates in the waste line, two valves were used in parallel.

The valves were powered using individual 24 V valve controller units (one per valve, part number 2503). The valve controller uses a 5 V TTL signal from the syringe drive to trigger the valves, with the voltage polarity reversed when the valves are closed to increase the pressure rating of the valve.

5.1.1.2 NMR tube nut

The NMR tube is secured using a custom designed nut, which we previously reported for use in an NMR *in-situ* mixing device (*Y. Gao, A. M. R. Hall, N. A. Fohn, E. J. King, L. A. L. Mitchell, G. A. Steedman, G. C. Lloyd-Jones, Eur. J. Org. Chem., 20, e202400095, 2024*). The NMR tube nut compresses two FFKM O-rings onto the NMR tube, providing a tight and chemically resistant seal that has been tested up to 12 bar continuous pressure without leaking. To achieve this seal, it is necessary to torque the NMR tube nut to 10 N.m. A similar design was previously reported by Morris *et. al.* (*M. Khajeh, M. A. Bernstein, G. A. Morris, Magn. Reson. Chem. 2010, 48, 516–522*).

5.2 SYRINGE DRIVE

A SF-73 two-syringe stepper motor drive (TgK Scientific) was with 2.5 mL drive syringes was used for all experiments. The stepper motor controller was modified to provide a 5V TTL output (labelled S6) from the syringe drive output 'Y4' with 3.3 kΩ pull-up resistor that could be triggered at an arbitrary time during the motor movement, and was used to trigger the NMR spectrometer along with the solenoid valves.

By using different sized syringes for the two reagents it is possible to deliver unequal volumes of reagent, however the ratio of the two reagents remains fixed unless the syringe size is changed. Care must also be taken to avoid creating uneven loads on the drive plate that may damage the stepper motor. Note that certain mixer designs such as the one used in this instrument require equal flow rates of both reagents to achieve effective mixing.

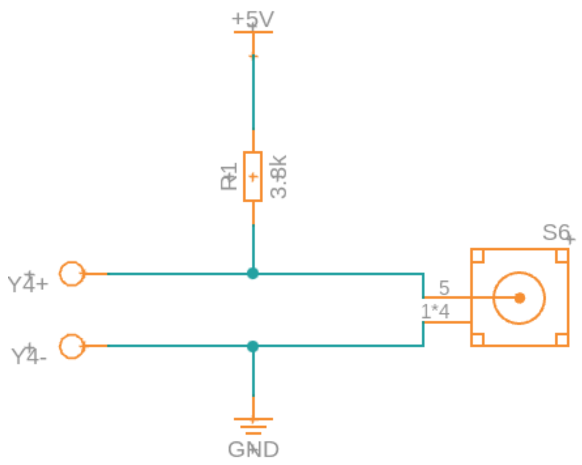

Figure S27: Wiring schematic for 5V TTL output

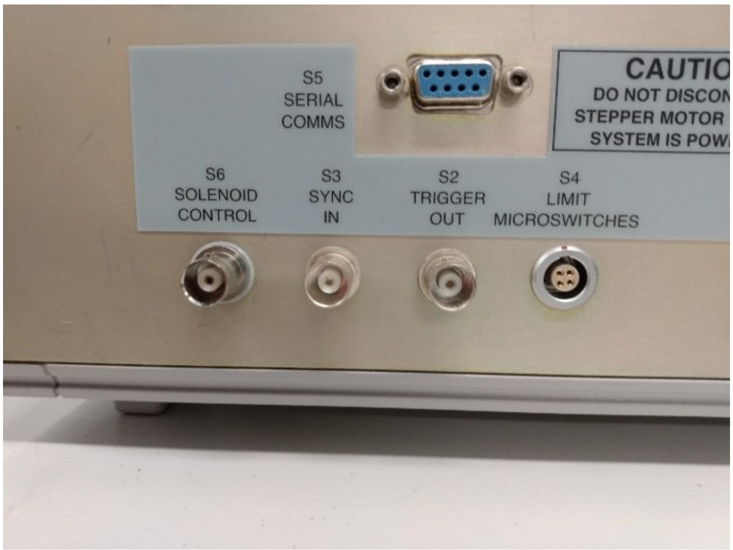

Figure S28: Photograph of the back of the syringe drive controller, showing 5V TTL output for valves labelled 'S6 Solenoid control'.

5.2.1 Syringe drive control script

Control of the stepper motor was achieved using KinetaDrive 3.06 software (TgK Scientific). The 'DC' command was used to set the point at which the solenoid valves should be triggered. For a standard 600 µL reaction volume, the trigger point was set at 1280 steps (533 µL) to occur just before the motor starts decelerating.

```
// FireSFNMR.script
// -------------------------------------------------------------------
// PRODUCT: SF-NMR-2
// Date: 01/11/2021
// Version: 1.00
// -------------------------------------------------------------------
!SIMPLE
// Stop and kill buffer
// Halts any buffered command in progress. Removes any other commands from buffer.
SK
// Energise the motor
ME
// Set-up shot parameters
AC[$SHOT_ACC]
DE[$SHOT_DEC]
VE[$V_SHOT]
// Store 10000ms in register 2
// This is used for the wait WD command below.
RX210000
// Set the driving distance
DI[$SHOTDISTANCE]
//
// Calculate the number of steps for decelleration of motor and
// subtract this from the total number of steps to determine when
// to trigger the solenoid valve.
// DC - set the distance in steps before the output is set in FOY4L
DC1280
// -------------------------------------------------------------------
// Pause (PS) suspends execution of buffered commands until (CT) is received.
PS
// -------------------------------------------------------------------
// START BUFFERED SCRIPT
// ===================================================================
// -------------------------------------------------------------------
// TRIGGERING
// Set the waiting for trigger indicator
// SS – Send String
SStrg1
// Re-arm Kinetic Studio for data collection.
SSadc
// WI - Wait for input (trigger) (H=high, L=low, R= rising edge, F=falling edge.)
//WI1F
// FO – Feed to Length and Set Output
// Same as Feed to Length (FL) but changes the state of an output during the move.
// Set output Y4 to low at distance DC-##
FOY4L
// -------------------------------------------------------------------
```

```
// Clear the waiting for trigger indicator
SStrg0
// Wait # ms as specified in RX2 command above.
WD2
// De-activate the solenoid
SOY4H
// Resume execution of buffered commands.
CT
// ==================================================================
// END OF BUFFERED SCRIPT
// ------------------------------------------------------------------
RS  // Request status - updates the indicators in KD
SSdone // Send a message to let KD know it can re-enable the control panel.
// ------------------------------------------------------------------
// Send a re-trigger request, let's go again!
SSarm0
```

5.3 NMR UMBILICAL

5.3.1 List of parts

UNIVERSITY OF EDINBURGH	LIST OF PARTS			AS2500-LOP
EQUIPMENT:	SF-NMR-2.5 UMBILICAL ASSEMBLY			SHEET 1 OF 2
**ITEM**	**PART REF.**	**DESCRIPTION**	**QTY**	**SUPPLIER/REMARKS**
1	620-1F	MICROPROBE TUBE 4", 2.95 x 43.5MM STEM	1	WILMAD
2	SF-NMR-3015	TUBE PROTECTOR	1	3D PRINT, PLA
3	SF-NMR-2511	SPINNER	1	CUSTOM MACHINED, PEEK
4	SF-NMR-2512	TUBE NUT	1	CUSTOM MACHINED, PEEK
5	BS 008	O-RING, KALREZ 6375, 4.9 ID x 1.9 SEC	2	BARNWELL MBMS0701 (NMR TUBE)
6	SF-NMR-2513	BOTTOM SEAL	1	CUSTOM MACHINED, PTFE
7	SF-NMR-2514	TOP SEAL	1	CUSTOM MACHINED, PTFE
8				
9	SF-NMR-2521	CAPILLARY BLOCK	1	CUSTOM MACHINED, PEEK
10	SF-NMR-2522	INNER CAPILLARY 1.7MM OD	1	MODIFIED WG-1365-1.7 (WILMAD)
11	SF-NMR-2523	OUTER CAPILLARY 3.0/1.7MM OD	1	MODIFIED 307-PP-9 (WILMAD)
12	P-343	ETFE FLANGELESS FERRULE, 3.0MM	1	COLE-PALMER
13	SF-NMR-2524	MODIFIED FERRULE, 1.7MM	1	COLE-PALMER (NO SS RING)
14				
15	SF-NMR-2531	PRE-MAGNETISATION BLOCK	1	CUSTOM MACHINED, PEEK
16				
17	SF-NMR-2541	PTFE WASHER, M22	1	CUSTOM MACHINED, PTFE
18				
19	12110	CHECK VALVE, HASTELLOY, 0.2BARG, FFKM	3	CAMBRIDGE REACTOR DESIGN
20	P-359	ETFE SUPERFLANGELESS FITTING, 1/8"	12	COLE-PALMER
21	P-259	ETFE SUPERFLANGELESS FITTING, 1/16"	3	COLE-PALMER
22	P-248	ETFE SUPERFLANGELESS FITTING, 1/16"	1	COLE-PALMER
23	M-650	SUPERFLANGELESS FERRULE, 1/16"	3	COLE-PALMER
24	P-387	SUPERFLANGELESS NUT, 1/8"	6	COLE-PALMER
25	P-336	SUPERFLANGELESS NUT, SHORT, 1/8"	6	COLE-PALMER
26				
27				
28				
29	NA7997	SPINNER ADAPTOR	1	CUSTOM MACHINED, PVC
30	NA7998	TUBE HOUSING	1	CUSTOM MACHINED, PVC
31	NA7999	BAYONET PIN	2	CUSTOM MACHINED, PVC
32	NA8005	MODIFIED CONDUIT GLAND	1	ADAPTAFLEX
33	NA8160	CONDUIT GLAND SEAL	1	CUSTOM MACHINED, PTFE
34	196-4742	O-RING, NITRILE, 20.35 ID x 1.78 SEC	1	RS (TUBE HOUSING)
35	196-546	O-RING, NITRILE, 18.1 ID x 1.6 SEC	3	RS (SPINNER ADAPTOR)
36				
37	1524L	FEP TUBING 3/16" x 0.125"	1.5M	COLE-PALMER (HEAT TRANSFER)
38	ASF16/M20/A/BL	CONDUIT GLAND, M20, 16 MM, BLACK	1	ADAPTAFLEX
39	186-5050	FLEXIBLE CONDUIT, 16 MM, BLACK	1.2 M	RS (ADAPTAFLEX 0P116/BLACK)
40	P-140	ETFE SUPERFLANGELESS FITTING, 3/16"	1	COLE-PALMER
41	P-137	SUPERFLANGELESS NUT, 5/16-24"	1	COLE-PALMER
42				
43	1648	ETFE TUBING 1/8" x 0.093" ID	1.5 M	COLE-PALMER (WASTE LINE)
44	1530	ETFE TUBING 1/8" x 1/16" ID	2.4 M	COLE-PALMER (DRIVE LINES)
45	1517	ETFE TUBING 1/16" x 0.040" ID	1.5 M	COLE-PALMER (FLUSH LINE)
46				
47				
48	NA8070	M6 1/4-28 TUBE ADAPTOR	1	CUSTOM MACHINED, 316 S STEEL
49				
50				
DATE	13/03/2023	14/03/2023		
ISSUE	v13	v14		

UNIVERSITY OF EDINBURGH	**LIST OF PARTS**			AS2500-LOP
EQUIPMENT:	SF-NMR-2.5 UMBILICAL ASSEMBLY			SHEET 2 OF 2
**ITEM**	**PART REF.**	**DESCRIPTION**	**QTY**	**SUPPLIER/REMARKS**
51	PT100	RTD PROBE, 5M CABLE, 1/10 DIN	1	OMEGA (HSRTD-3-100-1/10-5M)
52	458-3953	CABLE GLAND, M6	1	RS COMPONENTS
53	173-2067	LEMO FFA.1S.304.CLAC32 CIRCULAR PLUG	1	RS COMPONENTS (PT100 SENSOR)
54	399-776	RUBBER SLEEVE, H12	1	RS COMPONENTS
55				
56	SF-NMR-2501	UMBILICAL CONNECTOR	1	CUSTOM MACHINED, ALUMINIUM
57	SF-NMR-2551	HEAT TRANSFER MANIFOLD	1	CUSTOM MACHINED, ALUMINIUM
58	SF-NMR-2505	VALVE MANIFOLD	1	CUSTOM MACHINED, PEEK
59	196-4900	O-RING, NITRILE, 36 ID x 2 SEC	2	RS COMPONENTS
60	526-956	M3 x 10 SCREW, PAN	4	RS COMPONENTS
61	304-4615	M3 x 30 SCREW, HEX CAP	8	RS COMPONENTS
62	795-130	HOSE CONNECTOR, BSP 1/8", 8MM	2	RS COMPONENTS
63	P-316	PFA PLUG, 1/4"-28	2	COLE-PALMER (TEST PORTS)
64				
65				
66	6724-A01	2-WAY SOLENOID VALVE, FFKM 1.2MM, 24V	2	BURKERT (PART NO. 00281506)
67	6724-T01	3-WAY SOLENOID VALVE, FFKM 1.2MM, 24V	2	BURKERT (PART NO. 00276458)
68	2503	WISPERVALVE BOOST CONTROLLER	4	BURKERT (PART NO. 00689998)
69	134-6930	POWER SUPPLY, 24V, 0.75A	1	RS COMPONENTS
70	144-0932	BNC PLUG, FEMALE, PANEL MOUNT	1	RS COMPONENTS
71	2908905	POWER CONNECTOR, 2.1MM, 500mA	1	FARNELL
72				
73	SF-73	STEPPER MOTOR DRIVE	1	TGK SCIENTIFIC
74				
75				
76				
77				
78				
79				
80				
81				
82				
83				
84				
85				
86				
87				
88				
89				
90				
91				
92				
93				
94				
95				
96				
97				
98				
99				
100				
DATE	13/03/2023	14/03/2023		
ISSUE	v13	v14		

5.3.2 Engineering drawings

Engineering drawings for custom parts are shown below. PDFs of all drawings along with CAD files (.STEP format) are available to download from the University of Edinburgh DataShare website: https://datashare.ed.ac.uk/

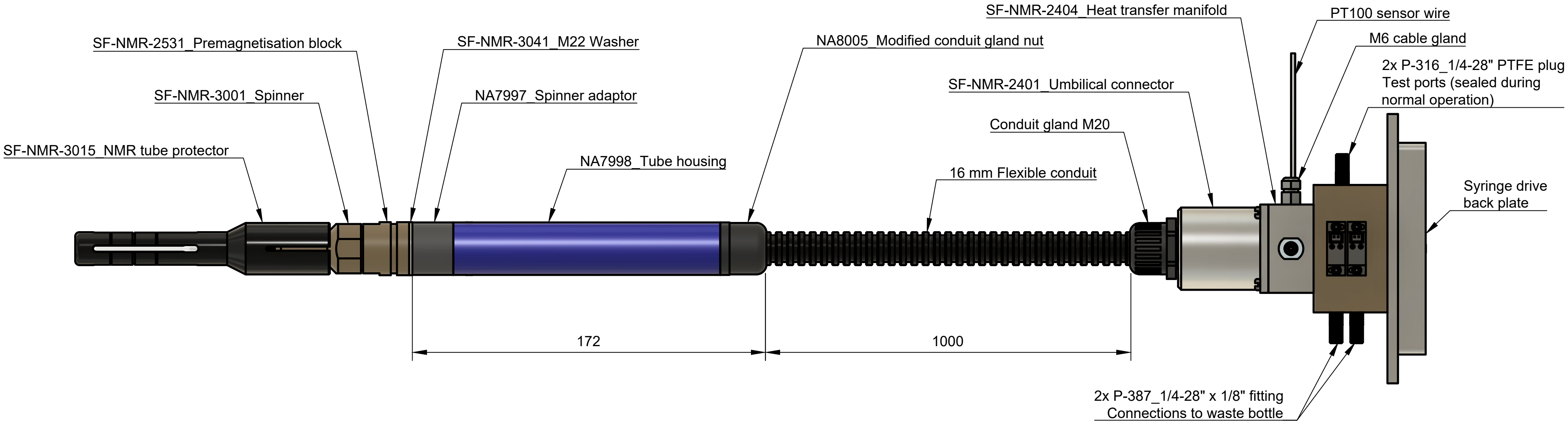

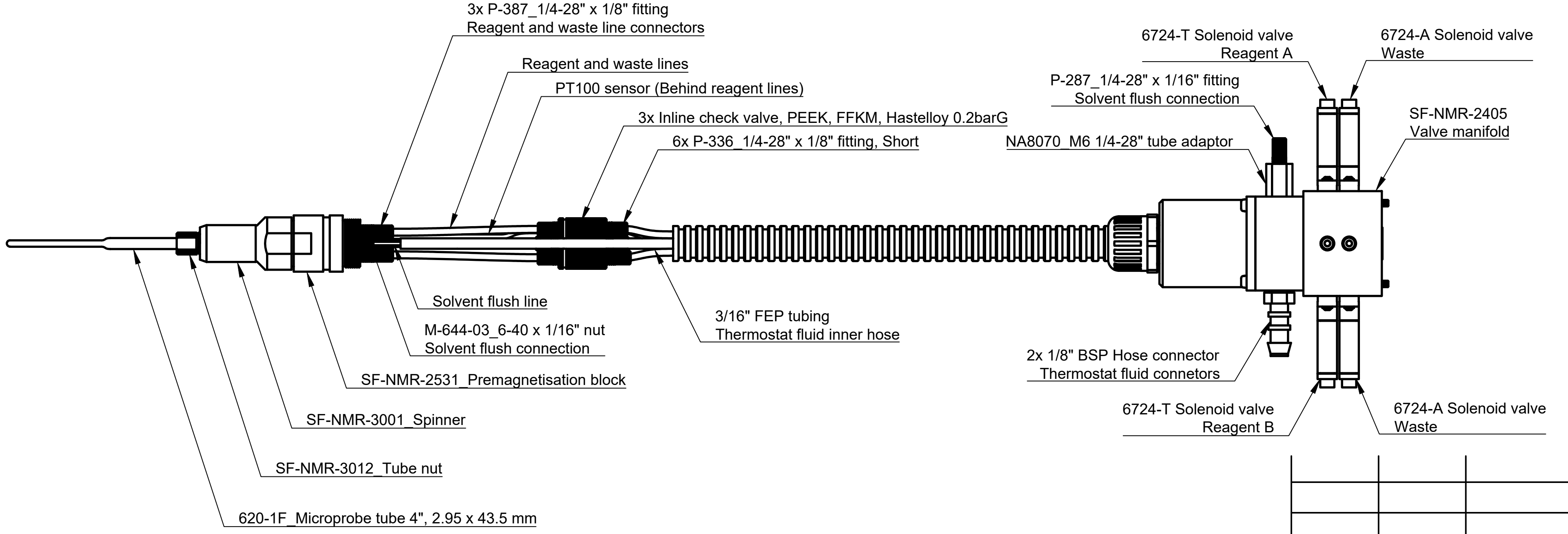

DO NOT SCALE<br>REMOVE ALL BURRS AND SHARP EDGES	Material	Finish	Date of issue<br>27/01/2026	Issue number<br>005	Modification
	Scale<br>1:2	Tolerance U.O.S.<br>± mm	Created by<br>Andrew Hall 27/01/2026		
DWG No.<br>SF-NMR-AS2500	Project<br>SF-NMR		UNIVERSITY OF EDINBURGH		
Title<br>SF-NMR-V2 Full assembly					Sheet<br>1/1

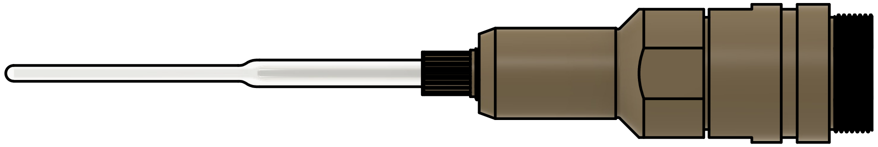

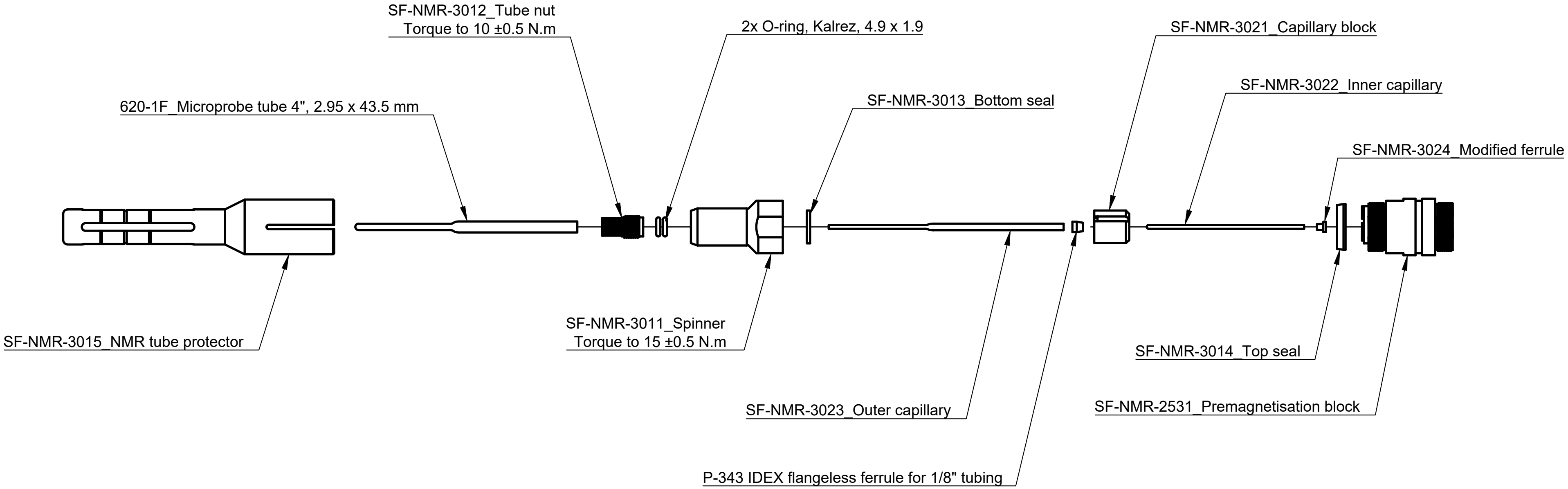

DO NOT SCALE<br>REMOVE ALL BURRS<br>AND SHARP EDGES	Material	Finish	Date of issue<br>27/01/2026	Issue number<br>007	Modification
	Scale<br>1:2	Tolerance U.O.S.<br>± mm	Created by<br>Andrew Hall 27/01/2026		
DWG No.<br>SF-NMR-AS2510	Project<br>SF-NMR		UNIVERSITY OF EDINBURGH		
Title<br>Spinner assembly					Sheet<br>1/1

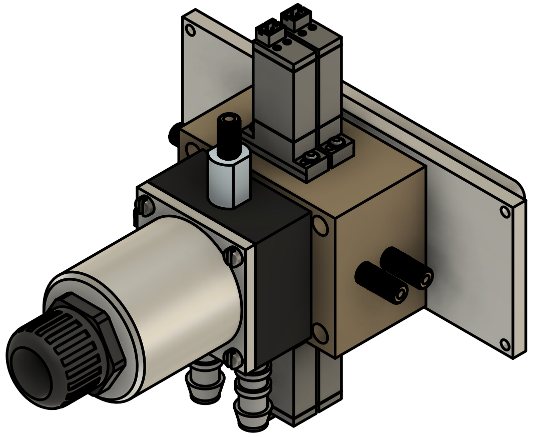

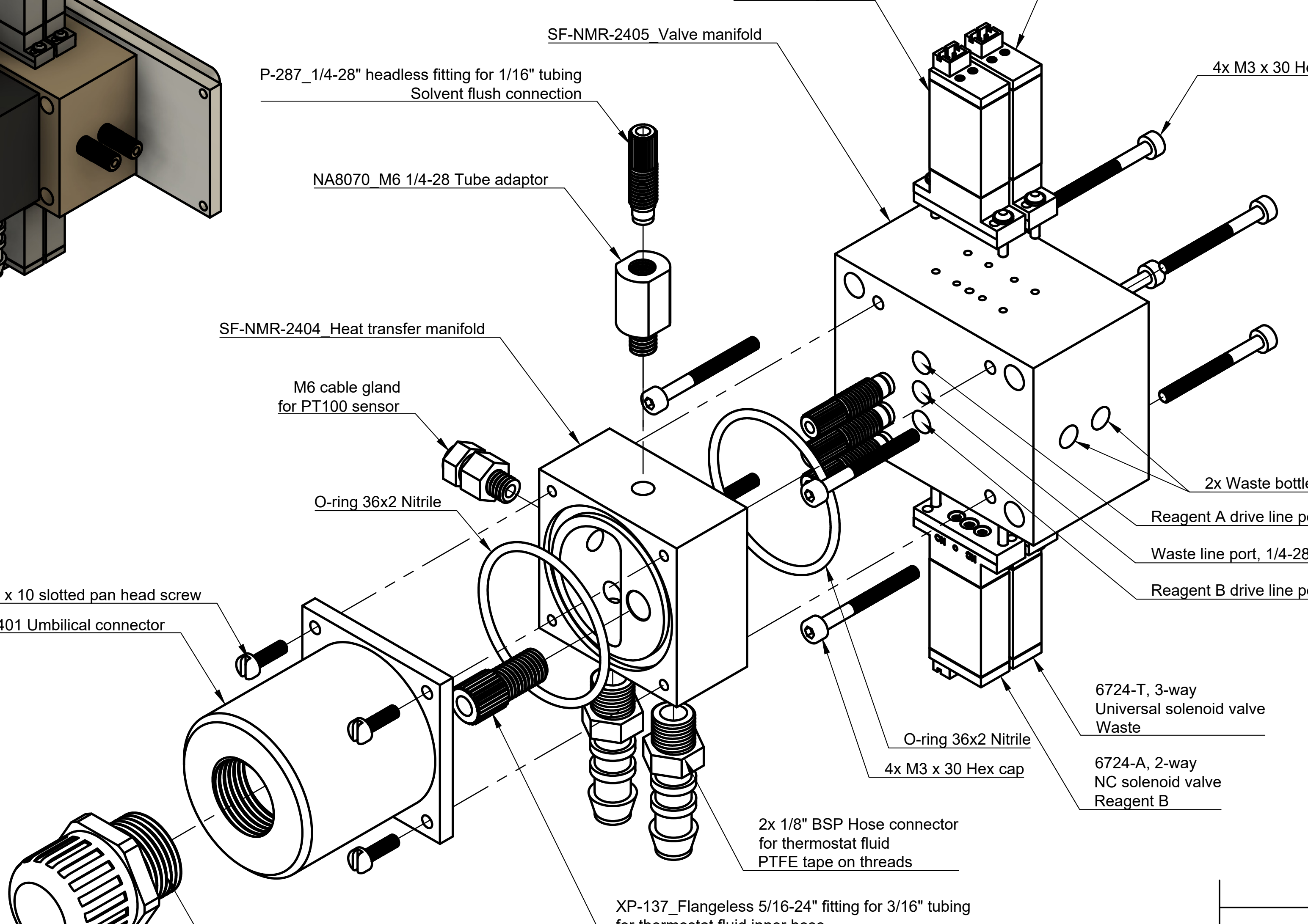

DO NOT SCALE
REMOVE ALL BURRS AND SHARP EDGES

Material	Finish	Date of issue: 27/01/2026	Issue number: 002	Modification
Scale: 2:3	Tolerance U.O.S.: ± mm	Created by: Andrew Hall 14/03/2023		

DWG No. SF-NMR-AS2550

Project: SF-NMR

UNIVERSITY OF EDINBURGH

Title: Umbilical connector assembly

Sheet 1/1

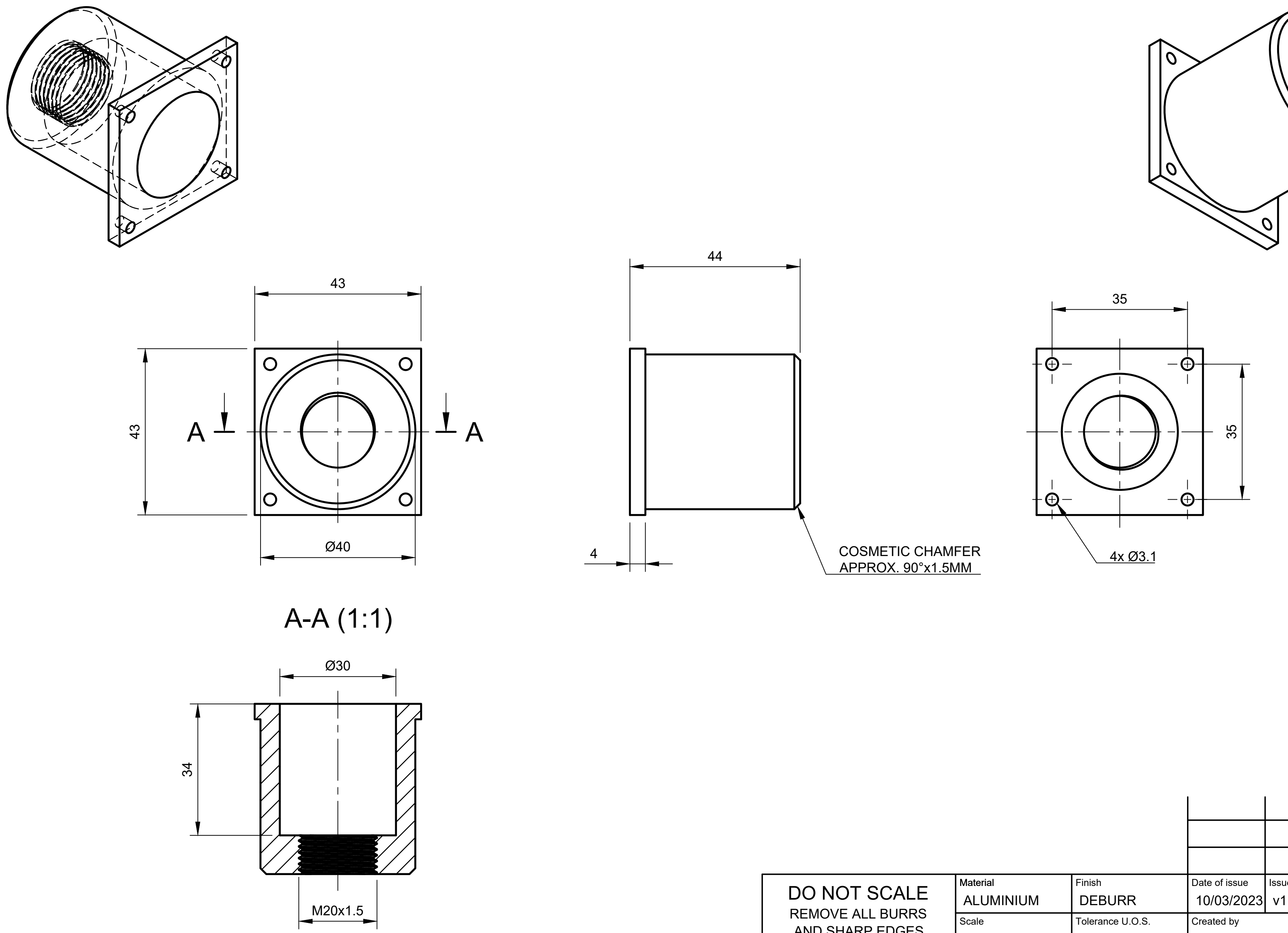

DO NOT SCALE<br>REMOVE ALL BURRS<br>AND SHARP EDGES	Material: ALUMINIUM	Finish: DEBURR	Date of issue: 10/03/2023 · Issue number: v1 · Modification:
	Scale: 1:1	Tolerance U.O.S.: ±0.2 mm	Created by: Andrew Hall 10/03/2023
DWG No.: SF-NMR-2401	Project: SF-NMR		UNIVERSITY OF EDINBURGH

Title: UMBILICAL CONNECTOR

Sheet: 1/1

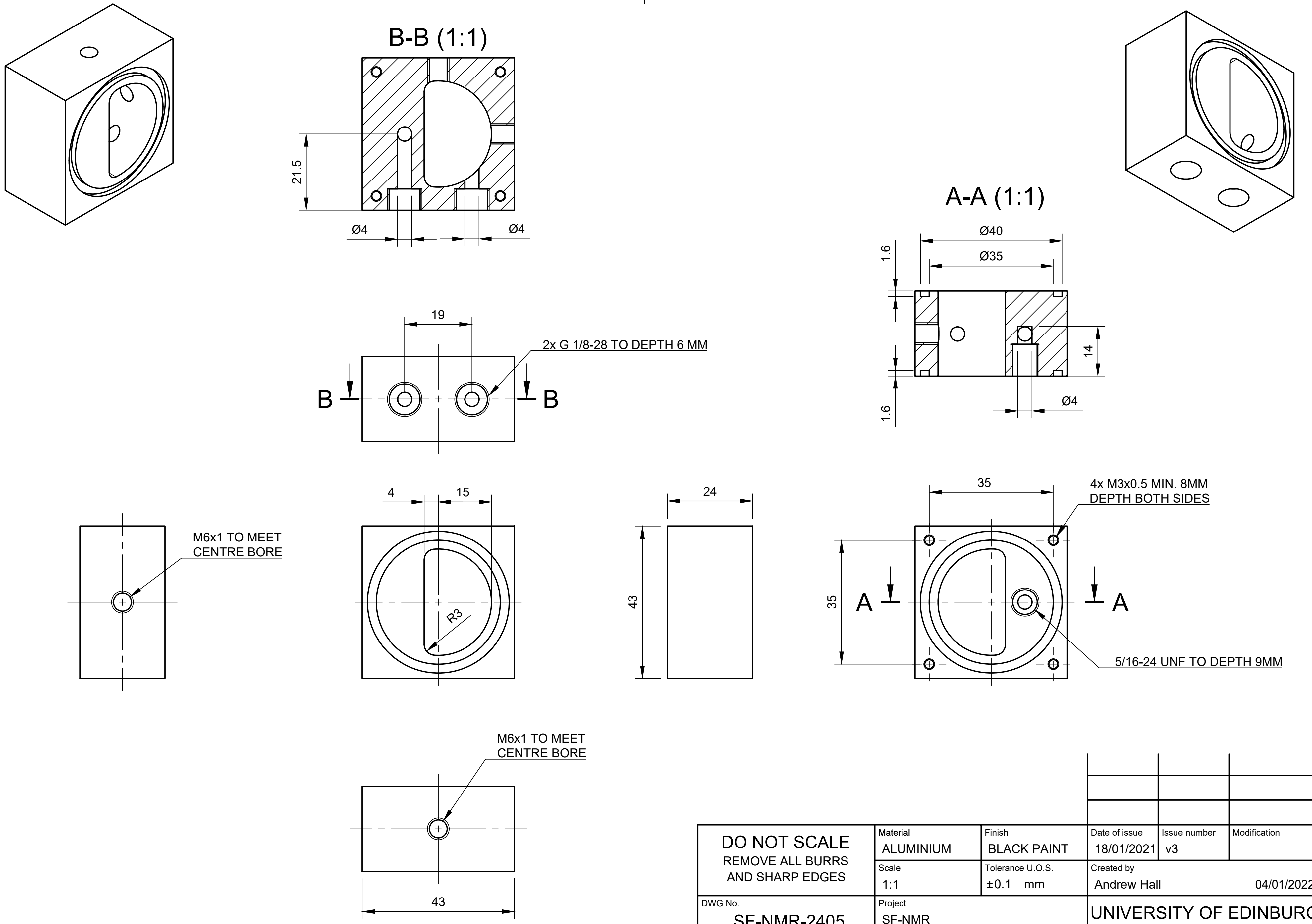

DO NOT SCALE<br>REMOVE ALL BURRS<br>AND SHARP EDGES	Material<br>ALUMINIUM	Finish<br>BLACK PAINT	Date of issue<br>18/01/2021	Issue number<br>v3	Modification
	Scale<br>1:1	Tolerance U.O.S.<br>±0.1 mm	Created by<br>Andrew Hall 04/01/2022		
DWG No.<br>SF-NMR-2405	Project<br>SF-NMR		UNIVERSITY OF EDINBURGH		
Title<br>Heat transfer manifold					Sheet<br>1/1

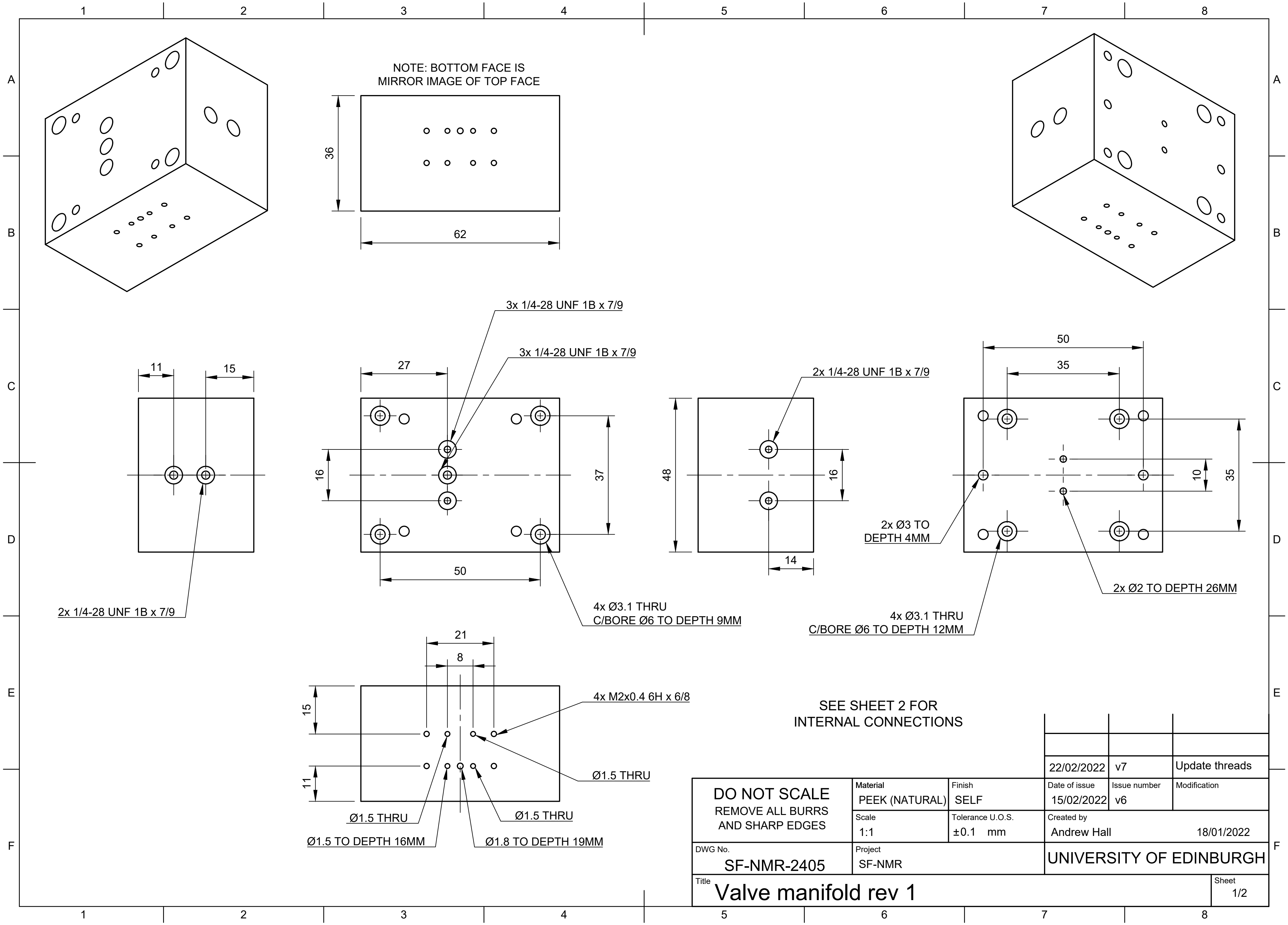
NOTE: BOTTOM FACE IS
MIRROR IMAGE OF TOP FACE
36
62
3x 1/4-28 UNF 1B x 7/9
3x 1/4-28 UNF 1B x 7/9
2x 1/4-28 UNF 1B x 7/9
11
15
27
16
37
50
48
16
14
50
35
10
35
2x 1/4-28 UNF 1B x 7/9
4x Ø3.1 THRU
C/BORE Ø6 TO DEPTH 9MM
2x Ø3 TO
DEPTH 4MM
2x Ø2 TO DEPTH 26MM
4x Ø3.1 THRU
C/BORE Ø6 TO DEPTH 12MM
21
8
15
11
4x M2x0.4 6H x 6/8
Ø1.5 THRU
Ø1.5 THRU
Ø1.5 THRU
Ø1.5 TO DEPTH 16MM
Ø1.8 TO DEPTH 19MM
SEE SHEET 2 FOR
INTERNAL CONNECTIONS
22/02/2022
v7
Update threads
DO NOT SCALE
REMOVE ALL BURRS
AND SHARP EDGES
Material
PEEK (NATURAL)
Finish
SELF
Date of issue
15/02/2022
Issue number
v6
Modification
Scale
1:1
Tolerance U.O.S.
±0.1 mm
Created by
Andrew Hall
18/01/2022
DWG No.
SF-NMR-2405
Project
SF-NMR
UNIVERSITY OF EDINBURGH
Title
Valve manifold rev 1
Sheet
1/2

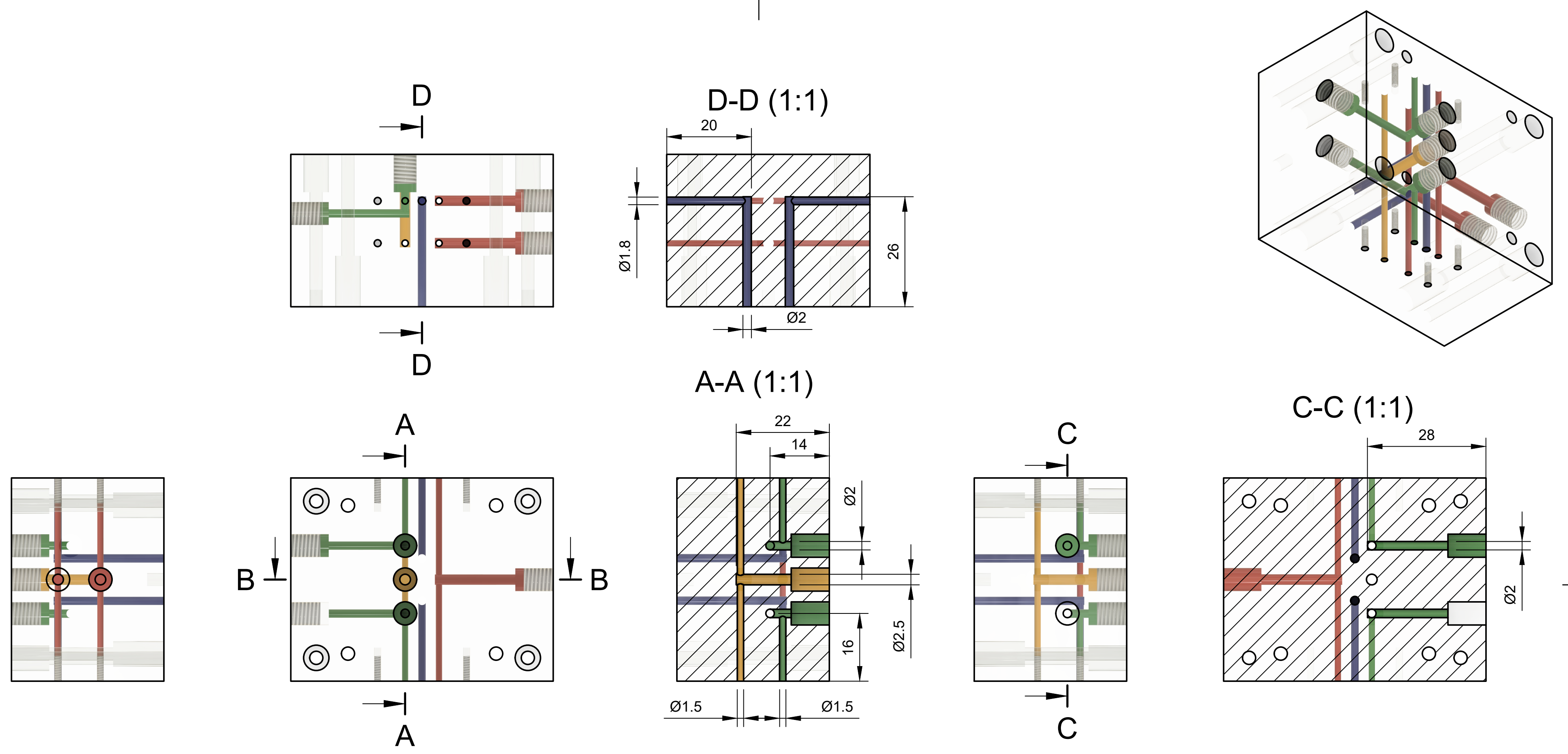

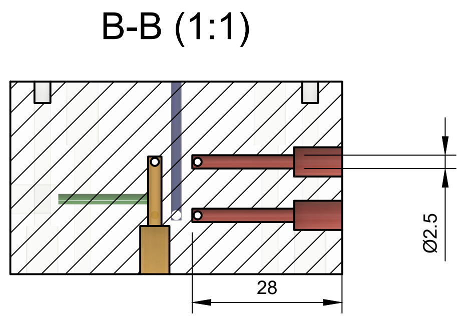

COLOURS FOR VISUAL AID ONLY

22/02/2022	v7	Update threads
Date of issue: 15/02/2022	Issue number: v6	Modification

DO NOT SCALE<br>REMOVE ALL BURRS AND SHARP EDGES	Material: PEEK (NATURAL)	Finish: SELF	
	Scale: 1:1	Tolerance U.O.S.: ±0.1 mm	Created by: Andrew Hall 18/01/2022
DWG No.: SF-NMR-2405	Project: SF-NMR		UNIVERSITY OF EDINBURGH
Title: Valve manifold rev 1			Sheet: 2/2

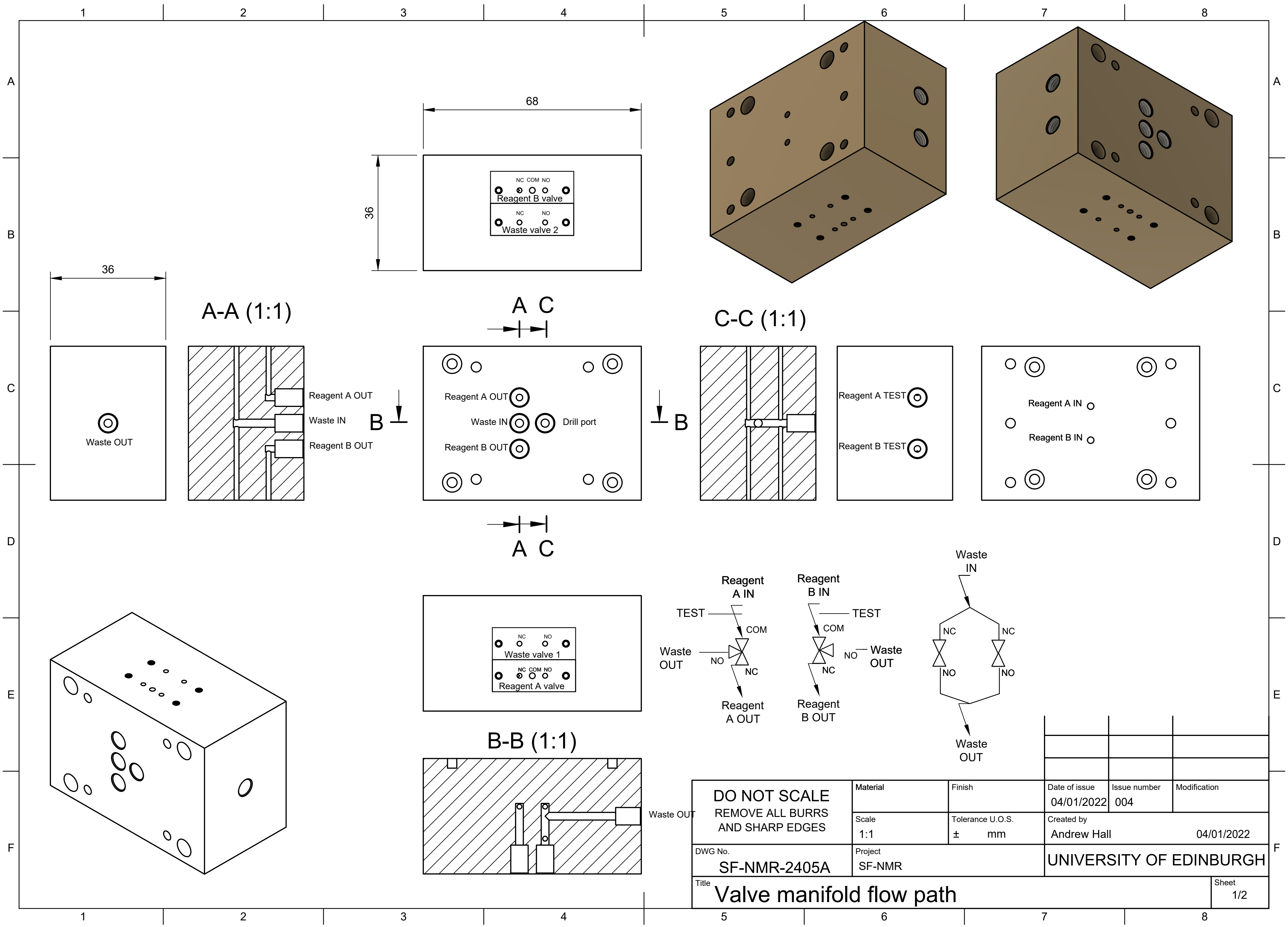

DO NOT SCALE<br>REMOVE ALL BURRS AND SHARP EDGES	Material	Finish	Date of issue<br>04/01/2022	Issue number<br>004	Modification
	Scale<br>1:1	Tolerance U.O.S.<br>± mm	Created by<br>Andrew Hall 04/01/2022		
DWG No.<br>SF-NMR-2405A	Project<br>SF-NMR		UNIVERSITY OF EDINBURGH		
Title<br>Valve manifold flow path					Sheet<br>1/2

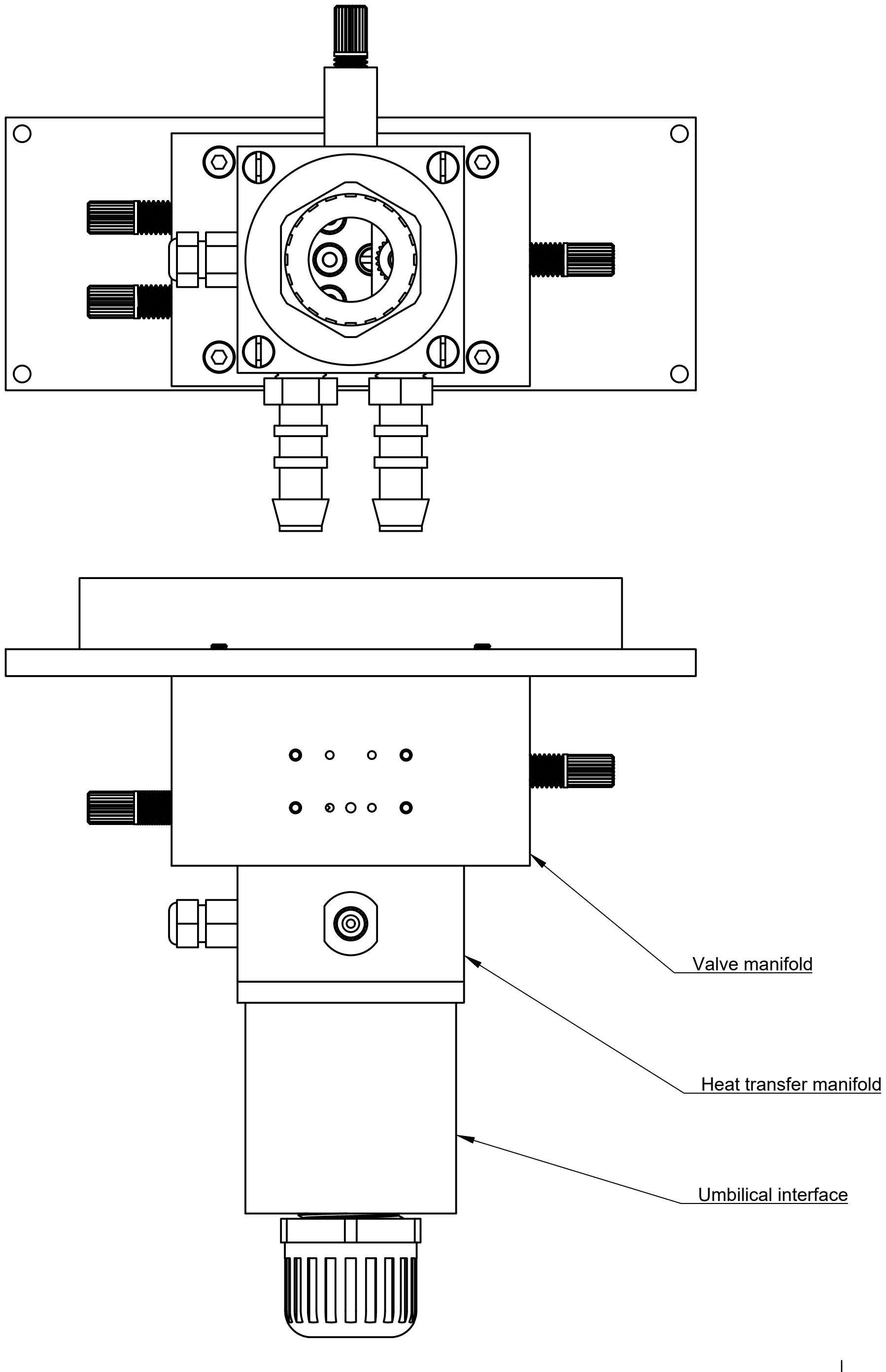

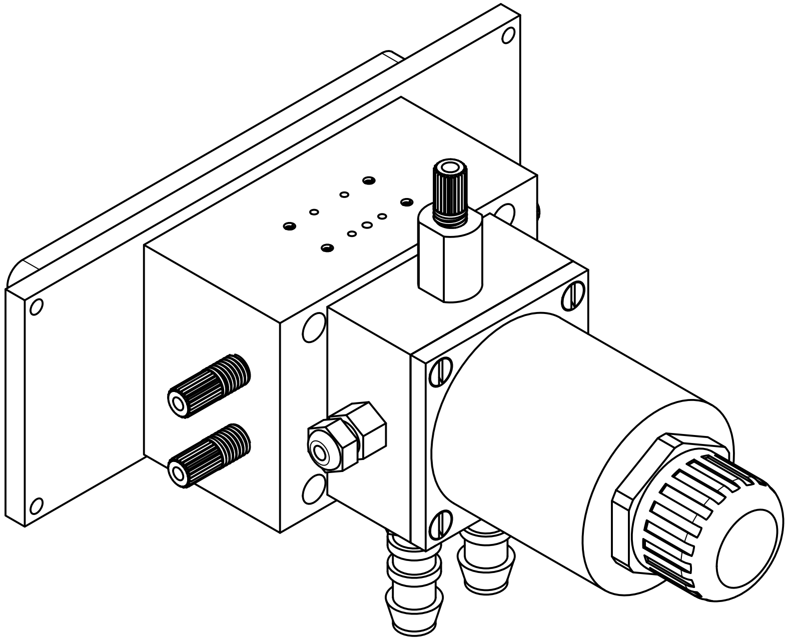

DO NOT SCALE<br>REMOVE ALL BURRS<br>AND SHARP EDGES	Material	Finish	Date of issue	Issue number	Modification
	Scale<br>1:1	Tolerance U.O.S.<br>± mm	Created by<br>Andrew Hall 04/01/2022		
DWG No.	Project<br>SF-NMR		UNIVERSITY OF EDINBURGH		
Title<br>Umbilical connector assembly rev 3					Sheet<br>2/2

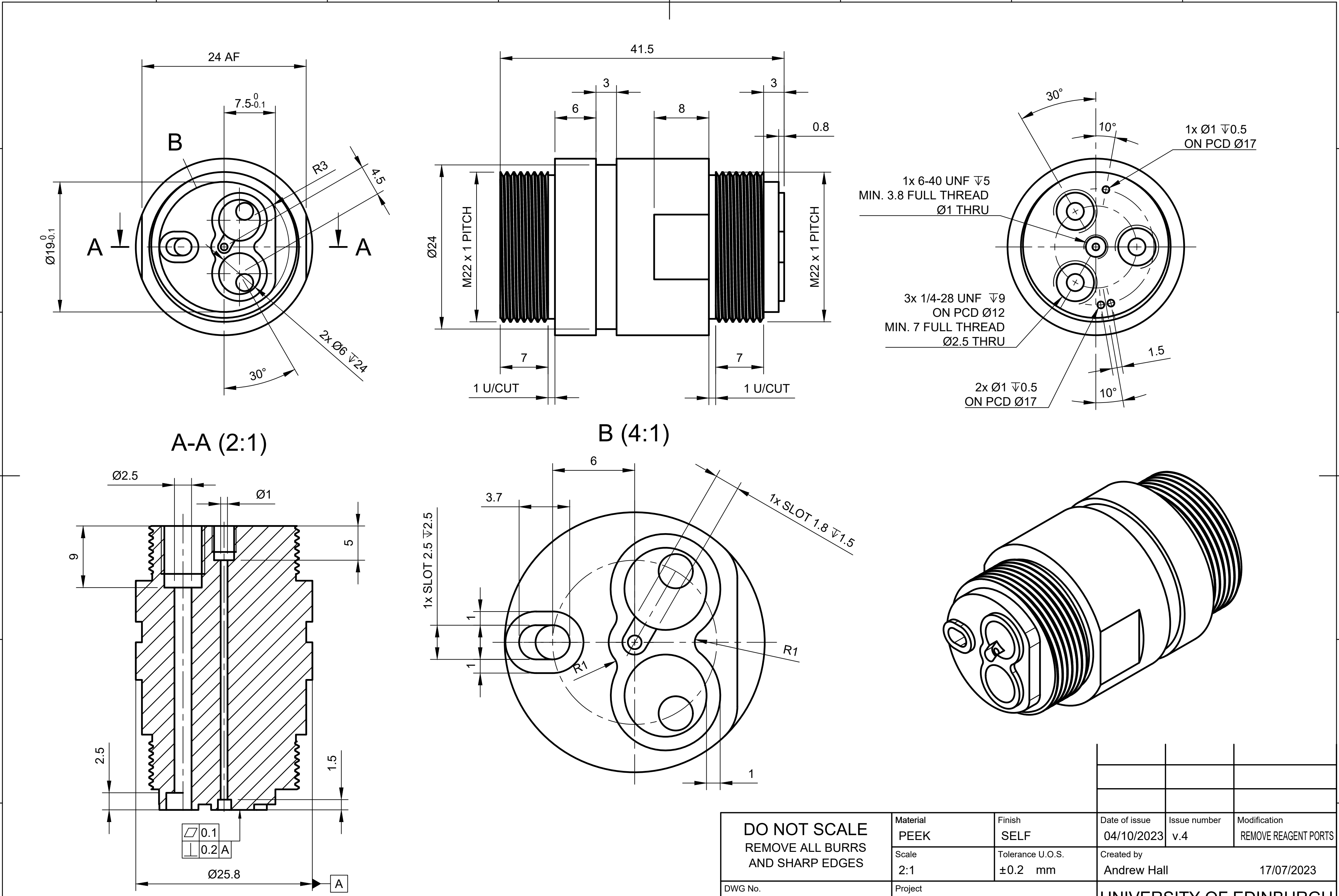

DO NOT SCALE<br>REMOVE ALL BURRS<br>AND SHARP EDGES	Material<br>PEEK	Finish<br>SELF	Date of issue<br>04/10/2023	Issue number<br>v.4	Modification<br>REMOVE REAGENT PORTS
	Scale<br>2:1	Tolerance U.O.S.<br>±0.2 mm	Created by<br>Andrew Hall 17/07/2023		
DWG No.<br>SF-NMR-2531	Project<br>SF-NMR		UNIVERSITY OF EDINBURGH		
Title<br>PREMAGNETISATION BLOCK					Sheet<br>1/1

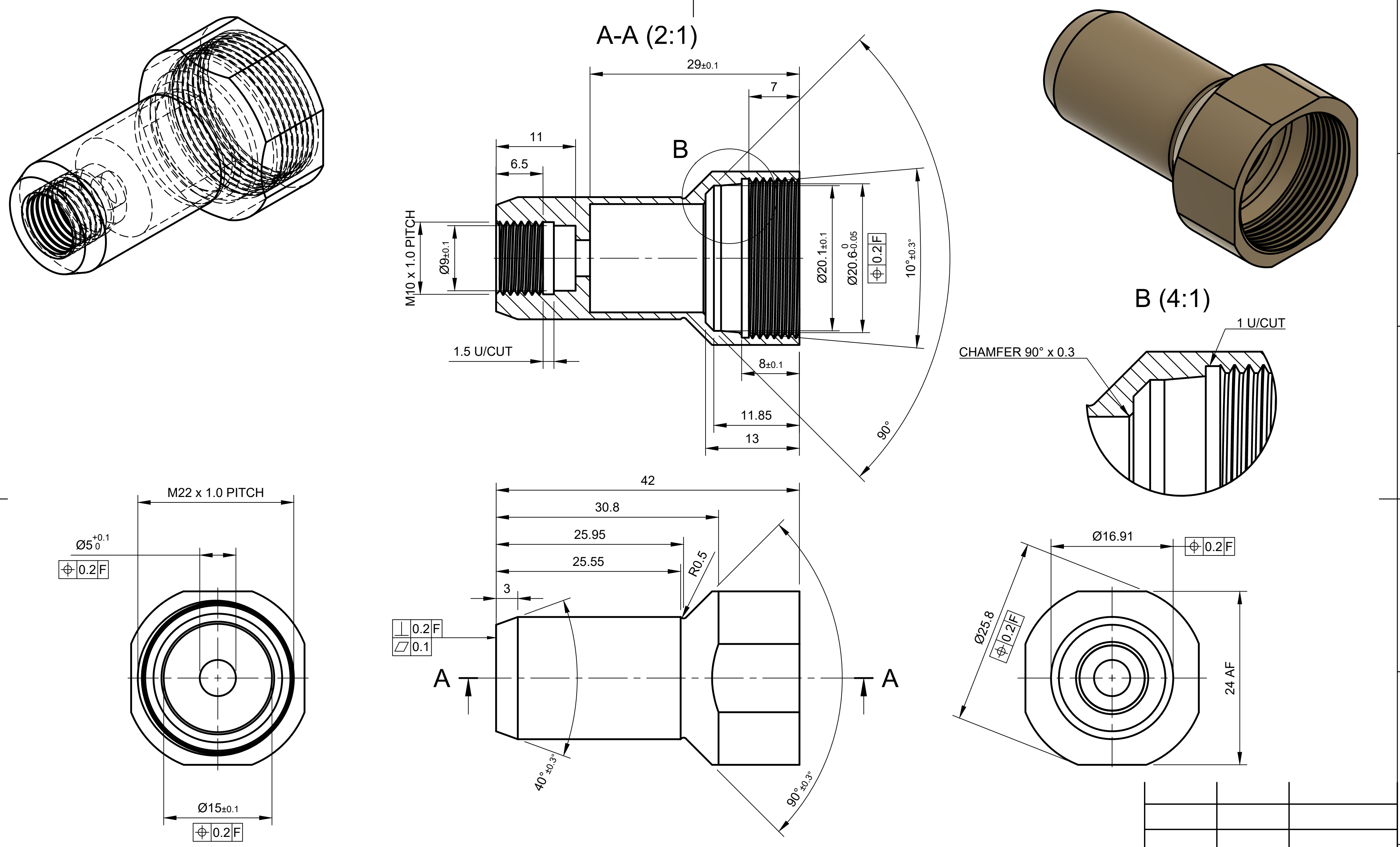

DO NOT SCALE<br>REMOVE ALL BURRS AND SHARP EDGES	Material<br>PEEK	Finish<br>SELF	Date of issue<br>24/02/2023	Issue number<br>v.9	Modification
	Scale<br>2:1	Tolerance U.O.S.<br>±0.2 mm	Created by<br>Andrew Hall 20/01/2023		
DWG No.<br>SF-NMR-3011	Project<br>SF-NMR		UNIVERSITY OF EDINBURGH		
Title<br>SPINNER					Sheet<br>1/1

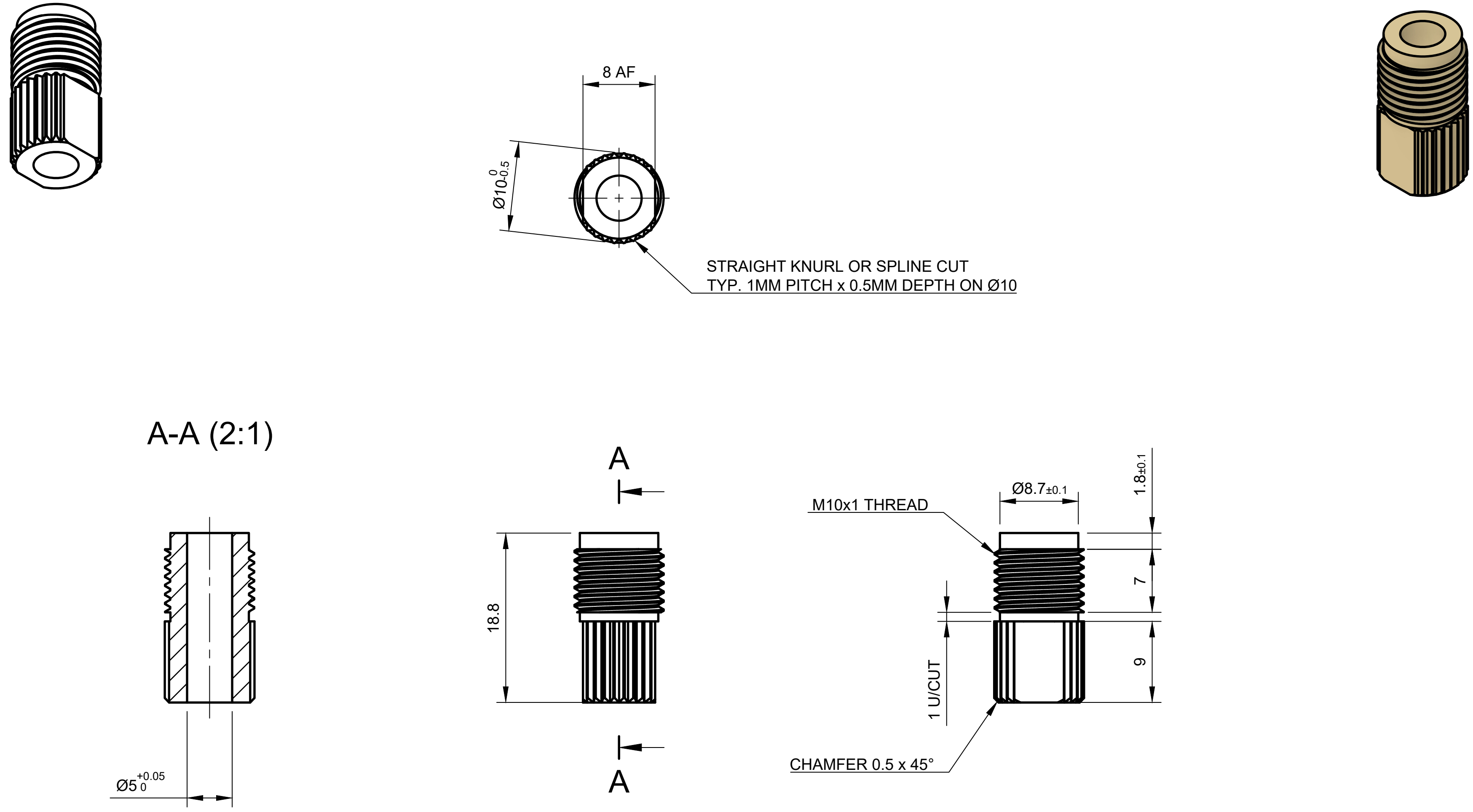

DO NOT SCALE<br>REMOVE ALL BURRS AND SHARP EDGES	Material<br>PEEK	Finish<br>SELF	Date of issue<br>13/03/2023	Issue number<br>v.4	Modification
	Scale<br>2:1	Tolerance U.O.S.<br>±0.2 mm	Created by<br>Andrew Hall 27/02/2023		
DWG No.<br>SF-NMR-3012	Project<br>SF-NMR		UNIVERSITY OF EDINBURGH		
Title<br>TUBE NUT					Sheet<br>1/1

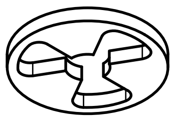

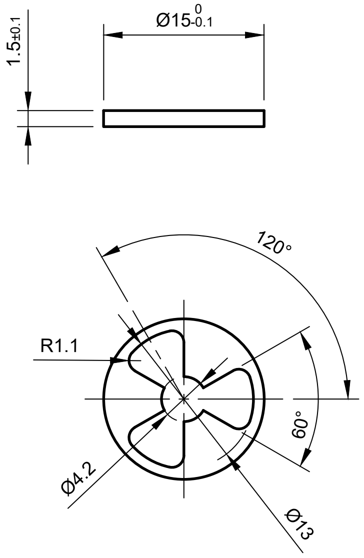

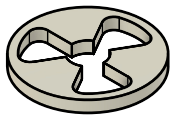

DO NOT SCALE<br>REMOVE ALL BURRS<br>AND SHARP EDGES	Material<br>PTFE	Finish<br>SELF	Date of issue<br>24/02/2023	Issue number<br>v.3	Modification
	Scale<br>2:1	Tolerance U.O.S.<br>±0.2 mm	Created by<br>Andrew Hall 24/02/2023		
DWG No.<br>SF-NMR-3013	Project<br>SF-NMR		UNIVERSITY OF EDINBURGH		
Title<br>BOTTOM SEAL					Sheet<br>1/1

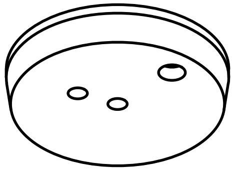

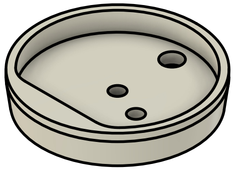

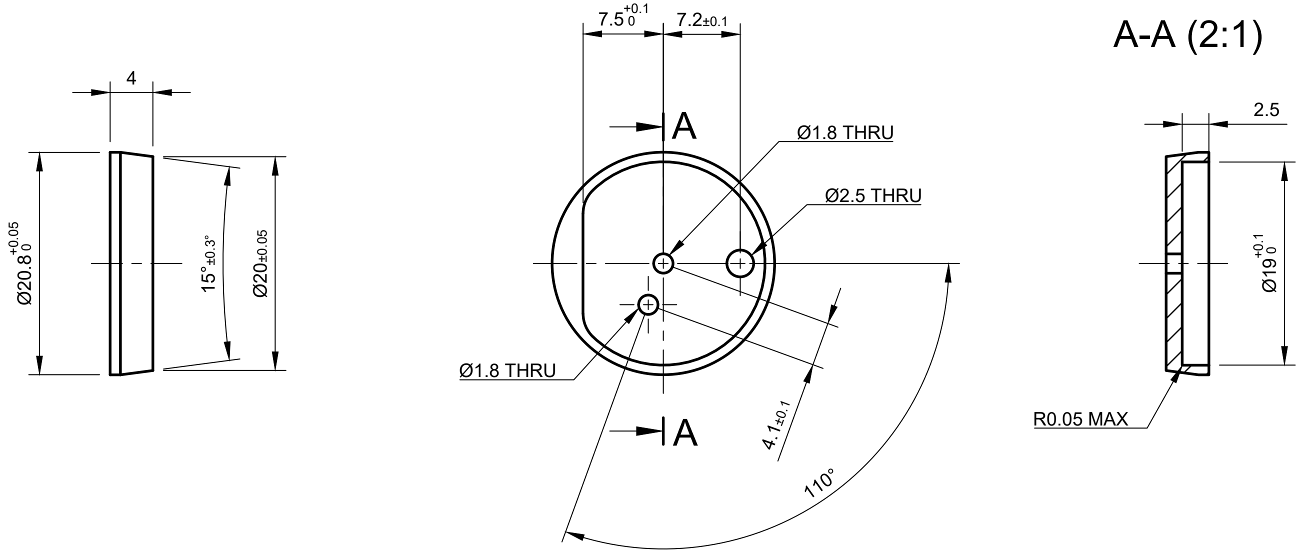

DO NOT SCALE<br>REMOVE ALL BURRS<br>AND SHARP EDGES	Material<br>PTFE	Finish<br>SELF	Date of issue<br>23/02/2023	Issue number<br>v.20	Modification
	Scale<br>2:1	Tolerance U.O.S.<br>±0.2 mm	Created by<br>Andrew Hall 19/01/2023		
DWG No.<br>SF-NMR-3014	Project<br>SF-NMR		UNIVERSITY OF EDINBURGH		
Title<br>TOP SEAL					Sheet<br>1/1

A-A (1:1)

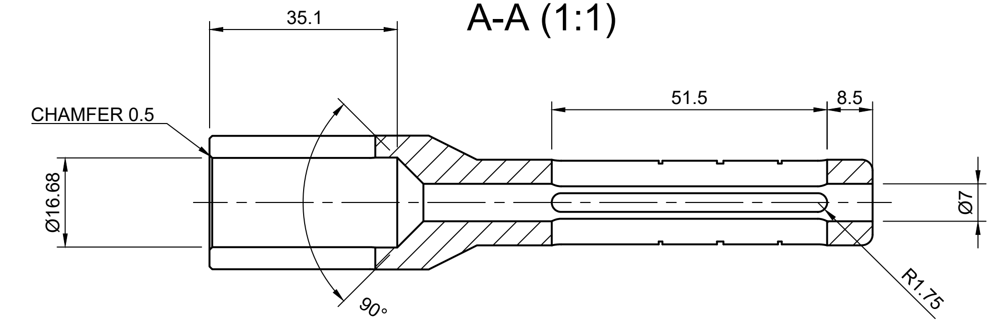

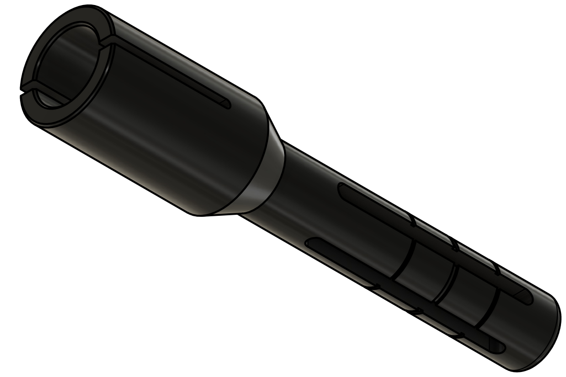

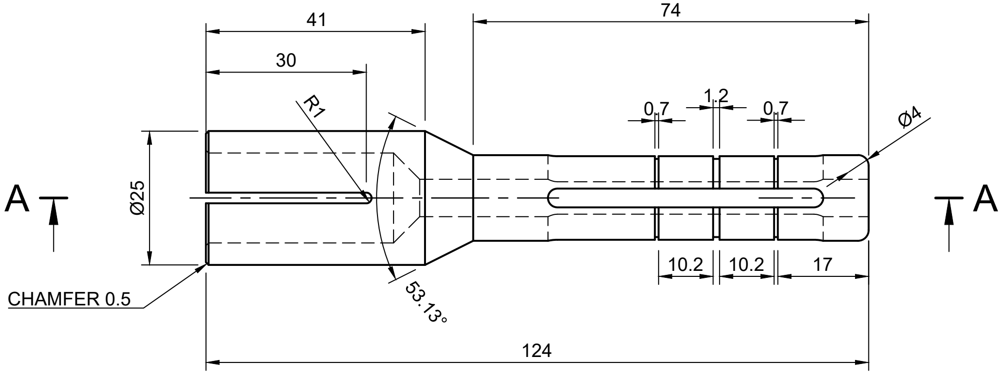

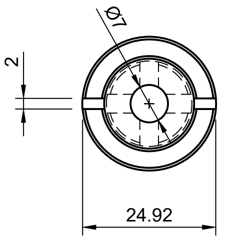

DO NOT SCALE
REMOVE ALL BURRS AND SHARP EDGES

Material	Finish	Date of issue	Issue number	Modification
PLA		27/01/2026	001	

Scale	Tolerance U.O.S.	Created by
1:1 (From parent)	±0.2 mm	Andrew Hall 27/01/2026

DWG No. SF-NMR-2515

Project SF-NMR

UNIVERSITY OF EDINBURGH

Title **NMR tube protector**

Sheet 1/1

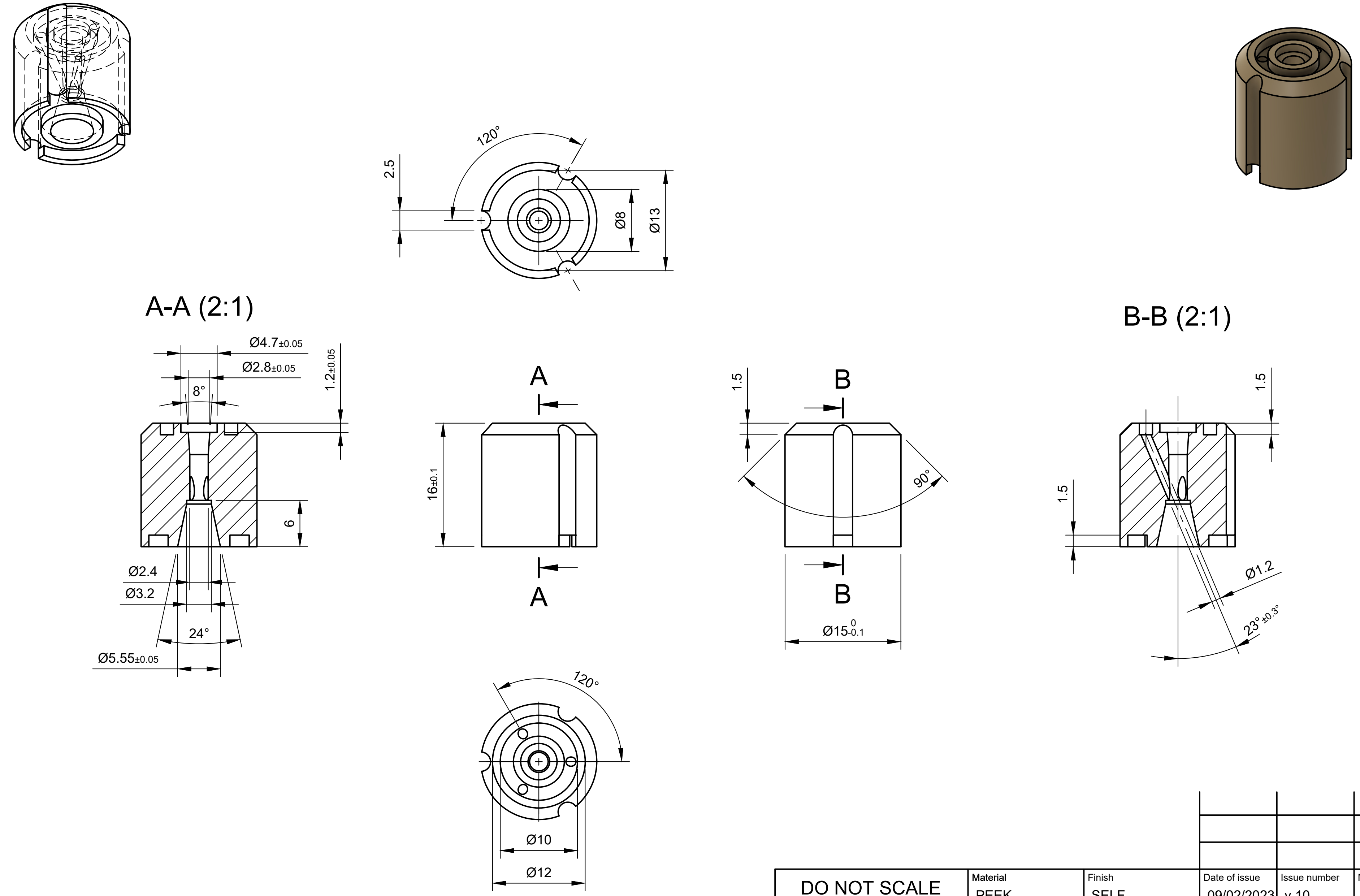

DO NOT SCALE<br>REMOVE ALL BURRS AND SHARP EDGES	Material<br>PEEK	Finish<br>SELF	Date of issue<br>09/02/2023	Issue number<br>v.10	Modification
	Scale<br>2:1	Tolerance U.O.S.<br>±0.2 mm	Created by<br>Andrew Hall 27/07/2022		
DWG No.<br>SF-NMR-3021	Project<br>SF-NMR		UNIVERSITY OF EDINBURGH		
Title<br>CAPILLARY BLOCK					Sheet<br>1/1

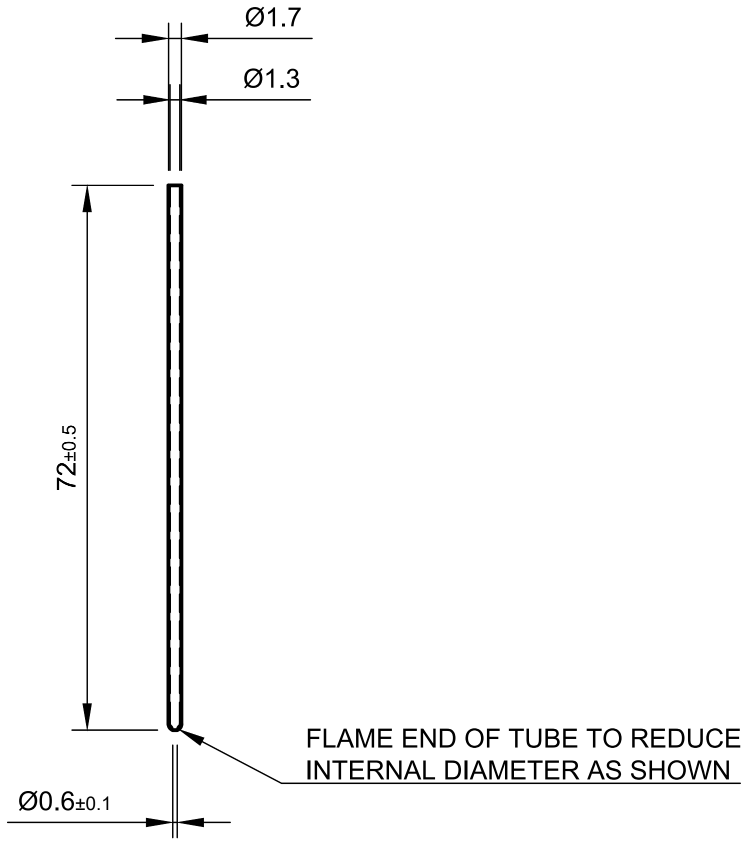

MODIFIED FROM WILMAD WG-1365-1.7

DO NOT SCALE<br>REMOVE ALL BURRS<br>AND SHARP EDGES	Material<br>BOROSILICATE GLASS	Finish	Date of issue<br>13/06/2023	Issue number<br>v.1	Modification
	Scale<br>1:1	Tolerance U.O.S.<br>±0.2 mm	Created by<br>Andrew Hall 13/06/2023		
DWG No.<br>SF-NMR-3023	Project<br>SF-NMR		UNIVERSITY OF EDINBURGH		
Title<br>INNER CAPILLARY					Sheet<br>1/1

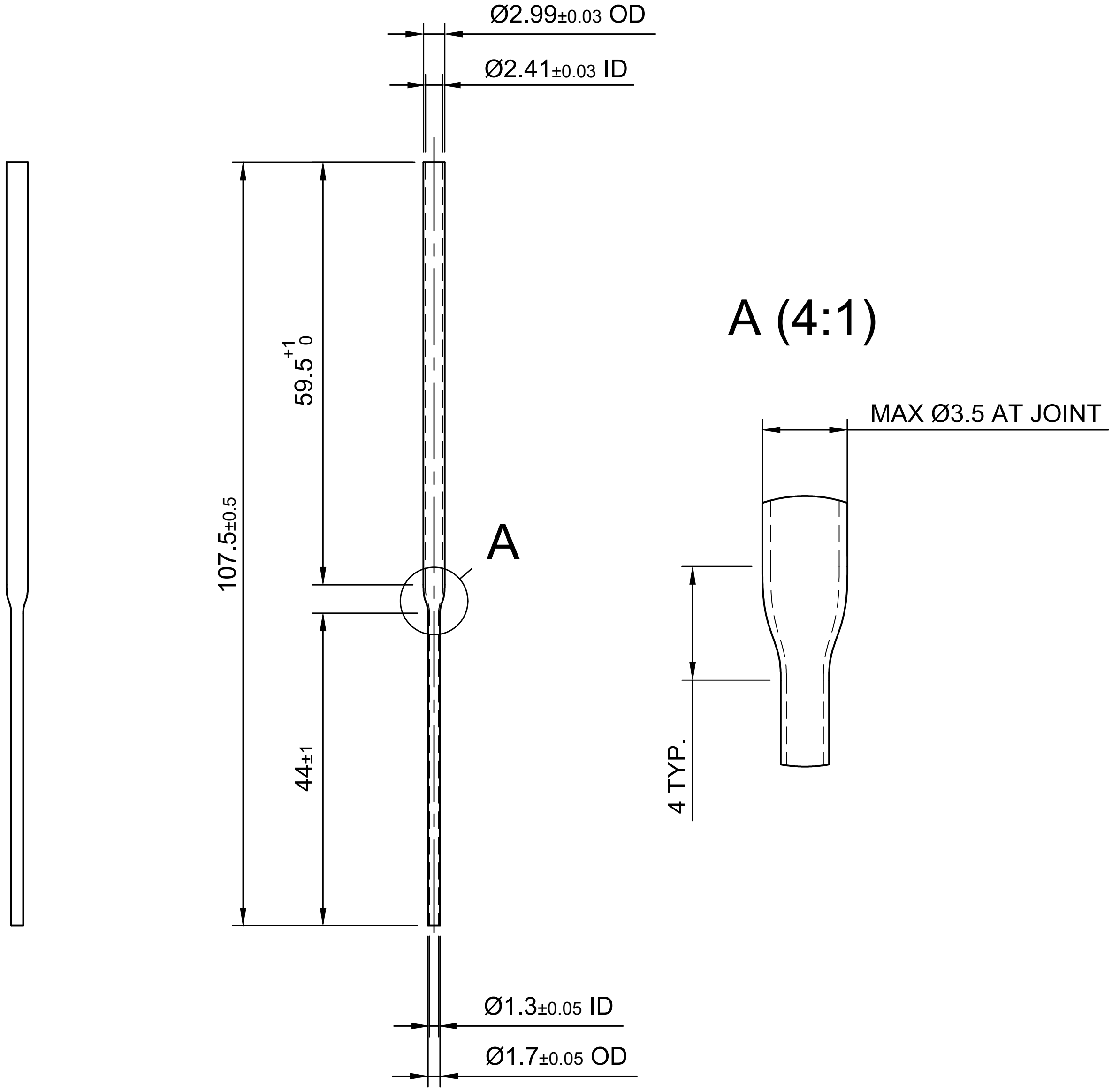

BOTH TUBES AS CONCENTRIC
AND PARALLEL AS POSSIBLE

BOTH ENDS OPEN

DO NOT SCALE<br>REMOVE ALL BURRS<br>AND SHARP EDGES	Material<br>Borosilicate glass, Type 1 Class A	Finish<br>Flame polish ends	Date of issue<br>11/08/2023	Issue number<br>v.6	Modification<br>Update finish
	Scale<br>1:1	Tolerance U.O.S.<br>±0.2 mm	Created by<br>Andrew Hall 16/03/2023		
DWG No.<br>SF-NMR-3023	Project<br>SF-NMR		UNIVERSITY OF EDINBURGH		
Title<br>OUTER CAPILLARY					Sheet<br>1/1

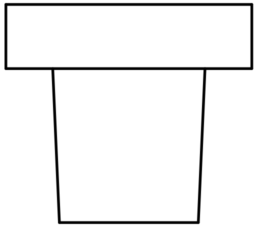

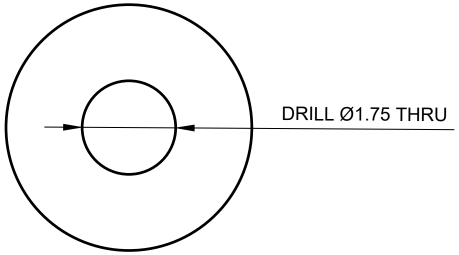

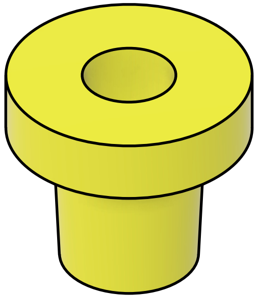

MODIFICATION TO P-259 SUPERFLANGELESS FERRULE, 1/16". STAINLESS STEEL RING NOT REQUIRED

DO NOT SCALE<br>REMOVE ALL BURRS AND SHARP EDGES	Material<br>ETFE	Finish<br>NONE	Date of issue<br>13/03/2023	Issue number<br>v.1	Modification
	Scale<br>10:1	Tolerance U.O.S.<br>±0.1 mm	Created by<br>Andrew Hall 13/03/2023		
DWG No.<br>SF-NMR-3024	Project<br>SF-NMR		UNIVERSITY OF EDINBURGH		
Title<br>MODIFIED FERRULE					Sheet<br>1/1

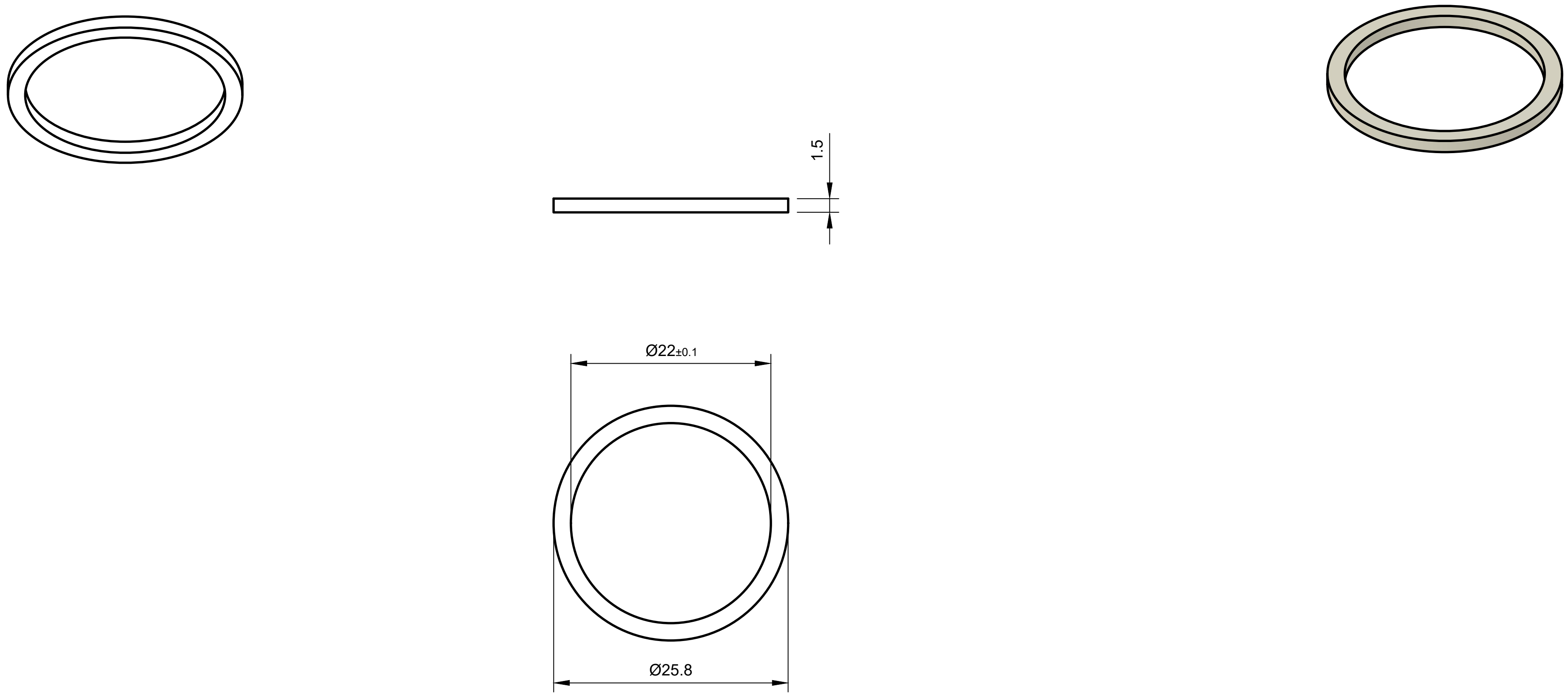

DO NOT SCALE<br>REMOVE ALL BURRS<br>AND SHARP EDGES	Material<br>PTFE	Finish<br>SELF	Date of issue<br>27/02/2023	Issue number<br>v.1	Modification
	Scale<br>2:1	Tolerance U.O.S.<br>±0.2 mm	Created by<br>Andrew Hall 27/02/2023		
DWG No.<br>SF-NMR-3041	Project<br>SF-NMR		UNIVERSITY OF EDINBURGH		
Title<br>M22 WASHER				Sheet<br>1/1	

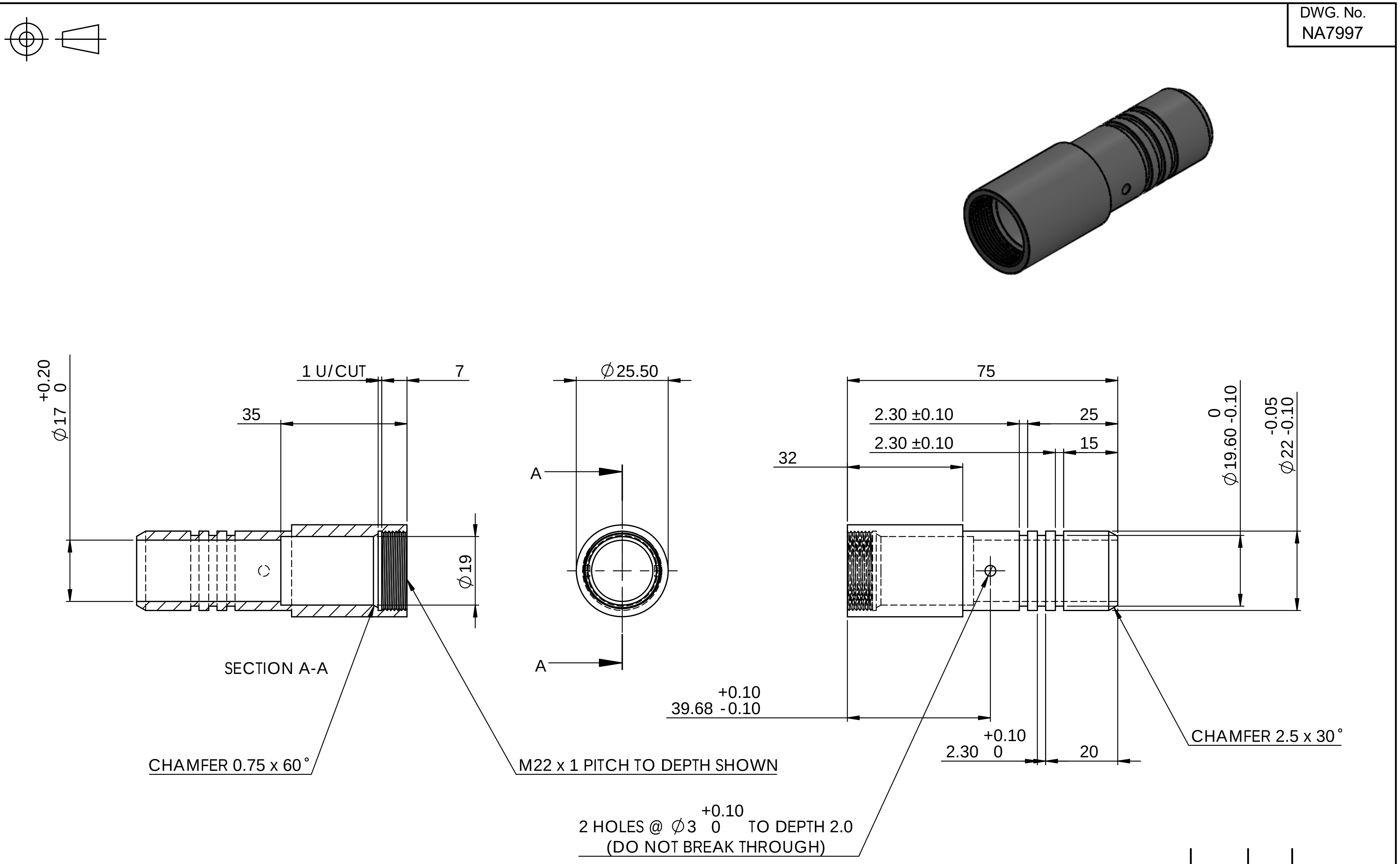

DATE	ISSUE	MODIFICATION
05.04.2016	2	ECN768
04.12.2015	1	

TgK Scientific Limited	DRAWN: MSP	MAT'L: GREY PVC	USED ON SF/NMR	-DO NOT SCALE- REMOVE ALL BURRS AND SHARP EDGES
7 LONGS YARD, ST. MARGARETS STREET BRADFORD ON AVON, BA15 1DH, UNITED KINGDOM Tel. +44 (0)1225 868699 Fax. +44 (0)1225 868633	DATE: 04.12.2015	FINISH: SELF	TOLERANCE U.OS. ±0.2mm	
	CHECKED:	TITLE SPINNER ADAPTOR		DWG. No. NA7997
	SCALE: 1:1			

DWG. No.
NA7998

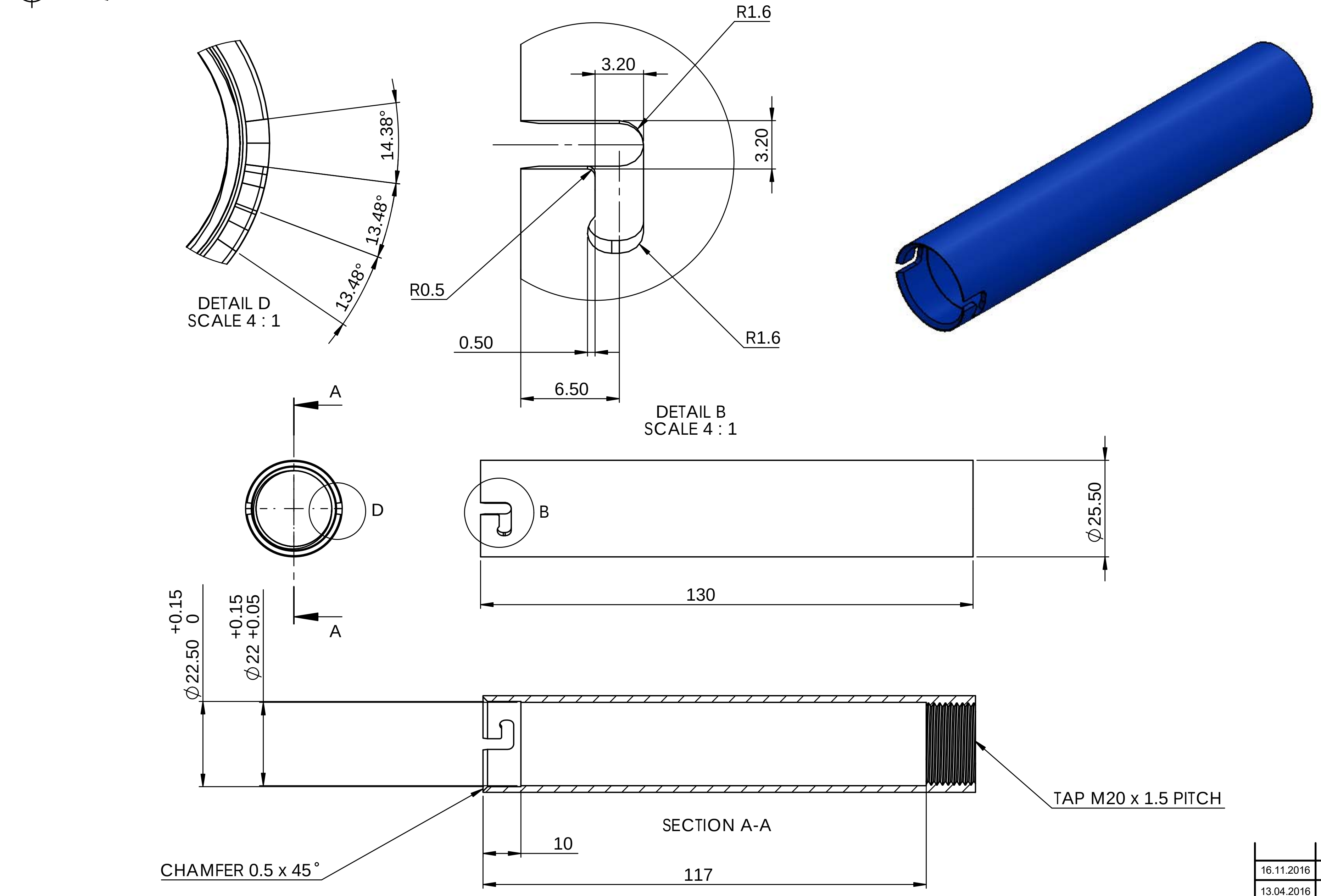

DATE	ISSUE	MODIFICATION
16.11.2016	3	ECN771
13.04.2016	2	ECN768
04.12.2015	1	

TgK Scientific Limited	DRAWN: MSP	MAT'L: BLUE PVC	USED ON SF/NMR	-DO NOT SCALE- REMOVE ALL BURRS AND SHARP EDGES
7 LONGS YARD, ST. MARGARETS STREET BRADFORD ON AVON, BA15 1DH, UNITED KINGDOM Tel. +44 (0)1225 868699 Fax. +44 (0)1225 868633	DATE: 04.12.2015	FINISH: SELF	TOLERANCE U.OS. ±0.2mm	
	CHECKED:	TITLE TUBE HOUSING		DWG. No. NA7998
	SCALE: 1:1			

DWG. No.
NA7999

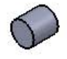

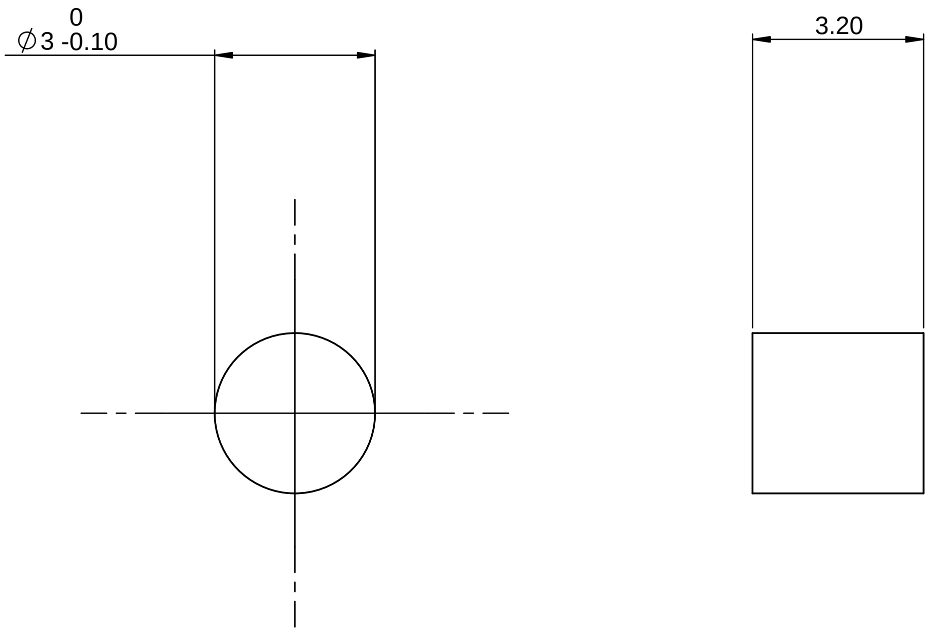

DATE	ISSUE	MODIFICATION
05.04.2016	2	ECN768
04.12.2015	1	

TgK Scientific Limited	DRAWN: MSP	MAT'L: GREY PVC	USED ON SF/NMR	-DO NOT SCALE- REMOVE ALL BURRS AND SHARP EDGES
7 LONGS YARD, ST. MARGARETS STREET BRADFORD ON AVON, BA15 1DH, UNITED KINGDOM Tel. +44 (0)1225 868699 Fax. +44 (0)1225 868633	DATE: 04.12.2015	FINISH: SELF	TOLERANCE U.OS. ±0.2mm	
	CHECKED:	TITLE BAYONET PIN		
	SCALE: 10:1			

DWG. No.
NA7999

DWG. No.
NA8005

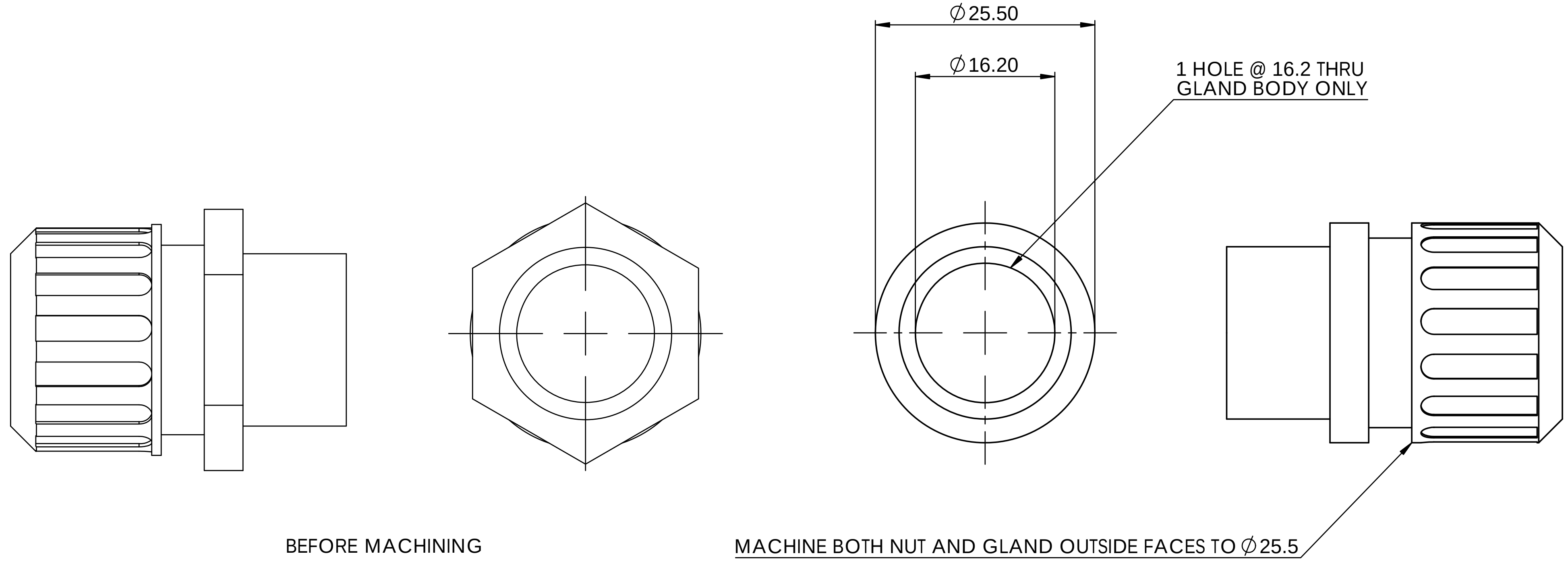

DATE	ISSUE	MODIFICATION
10.05.2016	2	ECN768
15.12.2015	1	

TgK Scientific Limited	DRAWN: MSP	MAT'L: SELF	USED ON SF/NMR	-DO NOT SCALE- REMOVE ALL BURRS AND SHARP EDGES
	DATE: 15.12.2015	FINISH: SELF	TOLERANCE U.OS. ±0.2mm	
7 LONGS YARD, ST. MARGARETS STREET<br>BRADFORD ON AVON, BA15 1DH, UNITED KINGDOM<br>Tel. +44 (0)1225 868699<br>Fax. +44 (0)1225 868633	CHECKED:	TITLE MODIFICATION - CONDUIT GLAND NUT		DWG. No. NA8005
	SCALE: 2:1			

DWG. No.
NA8070

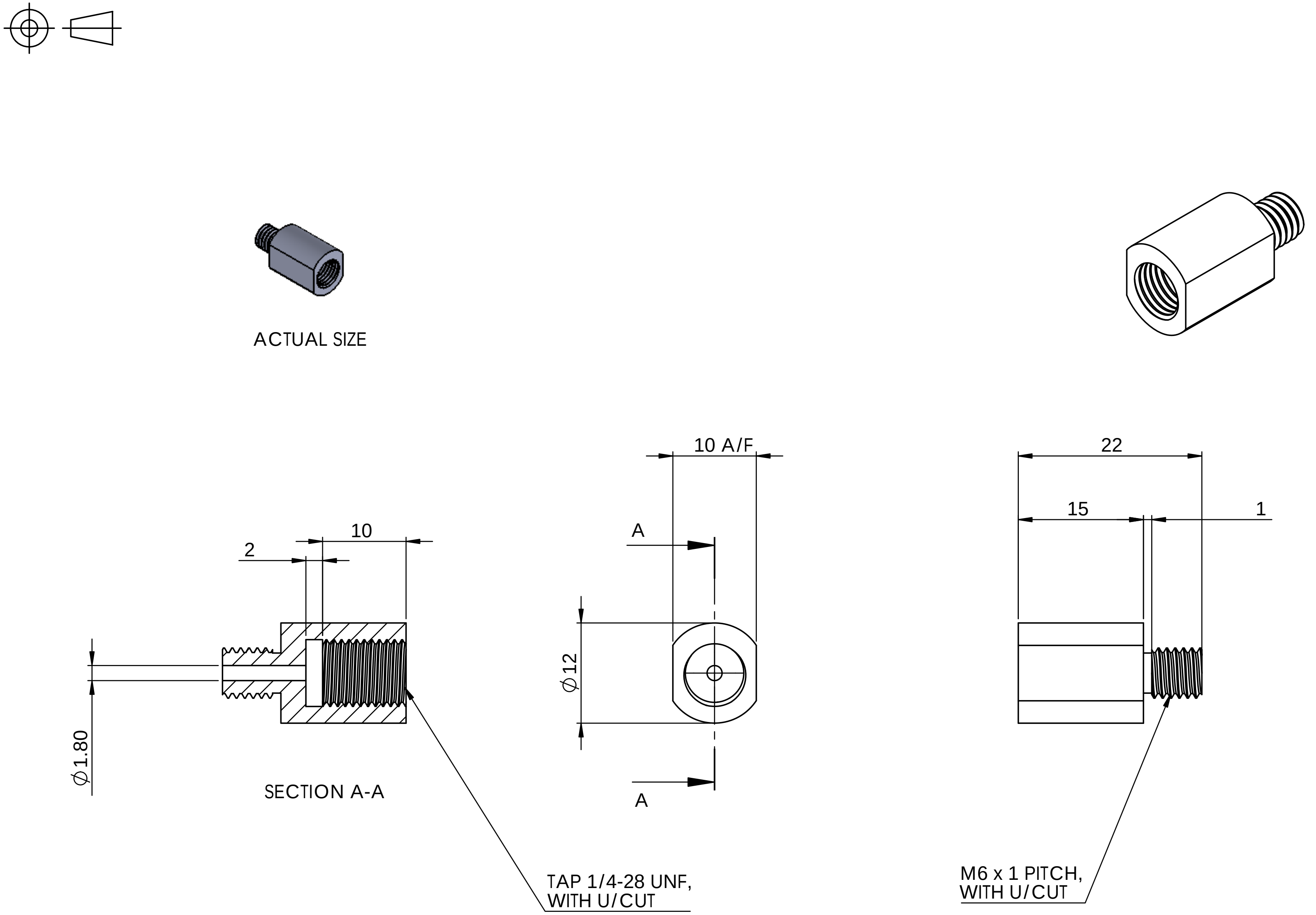

TgK Scientific Limited	DRAWN: MSP	MAT'L: 316 ST.STEEL	USED ON NC8033	-DO NOT SCALE- REMOVE ALL BURRS AND SHARP EDGES	15.04.2016	1	
	DATE: 15.04.2016	FINISH: SELF	TOLERANCE U.OS. ±0.2mm		DATE	ISSUE	MODIFICATION
7 LONGS YARD, ST. MARGARETS STREET BRADFORD ON AVON, BA15 1DH, UNITED KINGDOM Tel. +44 (0)1225 868699 Fax. +44 (0)1225 868633	CHECKED:	TITLE M6-1/4-28 TUBE ADAPTOR					DWG. No. NA8070
	SCALE: 2:1						

DWG. No.
NA8160

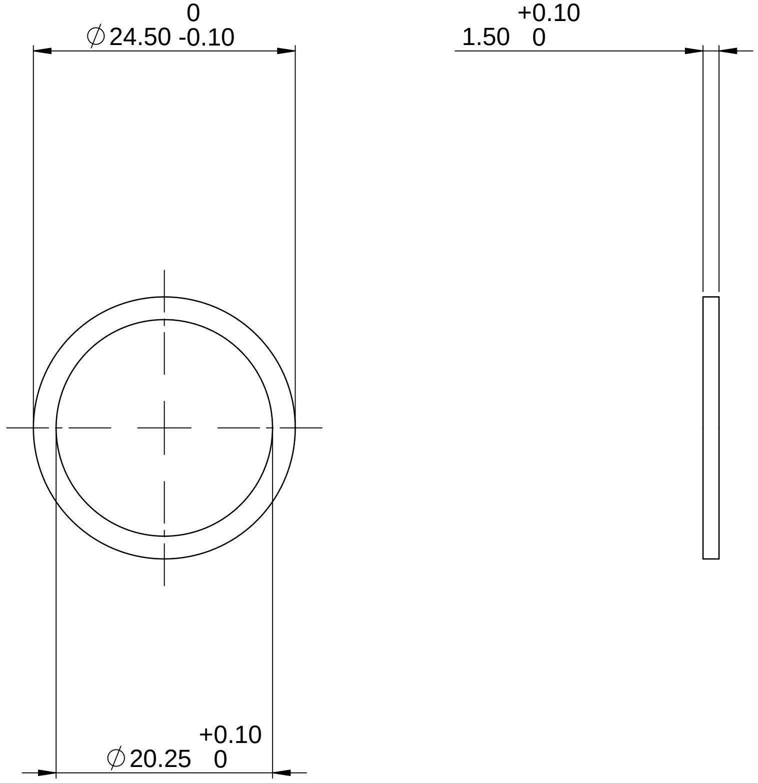

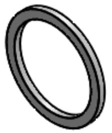

ACTUAL SIZE

TgK Scientific Limited	DRAWN: MSP	MAT'L: PTFE	USED ON NC8000	-DO NOT SCALE- REMOVE ALL BURRS AND SHARP EDGES	13.04.2018	1	ECN835
	DATE: 13.04.2018	FINISH: SELF	TOLERANCE U.OS. ±0.2mm		DATE	ISSUE	MODIFICATION
7 LONGS YARD, ST. MARGARETS STREET BRADFORD ON AVON, BA15 1DH, UNITED KINGDOM Tel. +44 (0)1225 868699 Fax. +44 (0)1225 868633	CHECKED:	TITLE CONDUIT GLAND SEAL					DWG. No. NA8160
	SCALE: 2:1						
